# Bio‐Assisted Synthesis of Reduced Graphene Oxide Nanosheets From Graphene Oxide: Promising and Efficient Cytotoxic and Antidiabetic Potency in In Vitro, Kinetic, and In Silico Models

**DOI:** 10.1155/ijbm/5521416

**Published:** 2026-02-17

**Authors:** Mansi Yadav, Divya Vashishth, Monika Bhardwaj, Jaya Parkash Yadav, Sudhir Kumar Kataria

**Affiliations:** ^1^ Department of Zoology, Ramjas College, University of Delhi, Delhi, 110007, India, du.ac.in; ^2^ Department of Zoology, Maharshi Dayanand University, Rohtak, Haryana, 124001, India, mdurohtak.ac.in; ^3^ Government Girls Senior Secondary School, Rurki, Rohtak, Haryana, 124001, India; ^4^ Department of Genetics, Maharshi Dayanand University, Rohtak, Haryana, 124001, India, mdurohtak.ac.in

**Keywords:** α-amylase, α-glucosidase, *Costus igneus*, glucose uptake assay, MTT assay, reduced graphene oxide

## Abstract

Green chemistry has recently made strides in the sustainable synthesis of next‐generation nanomaterials using reducing agents sourced from plants. Biocompatible conversion of reduced graphene oxide from graphene oxide nanomaterials is of particular interest in medical applications. The leaf extract of *C. igneus* functions as a reducing agent to produce reduced graphene oxide nanosheets from its precursor graphene oxide. A variety of characterization techniques were employed to confirm the formation and stability of reduced graphene oxide nanosheets. The cytotoxicity and glucose uptake potential of nanosheets have been evaluated through in vitro cell‐based assays. The MTT assay revealed a concentration‐dependent cytotoxic effect on Chang liver and U87MG cells, with maximum 84.5% and 76.3% cell viability, respectively. Reduced graphene oxide exhibited a significant rise in glucose uptake, comparable to the metformin drug, with increasing concentration. Enhanced glucose transport mediated by the nanosheets resulted in increased glucose uptake. Effective IC_50_ values of 84.46 μg·mL^−1^ and 93.83 μg·mL^−1^ were obtained in vitro enzyme inhibition studies against α‐amylase and α‐glucosidase, respectively. The enzyme kinetic investigation found noncompetitive inhibition for both enzymes. Molecular docking studies of *C. igneus* leaf derivatives were performed against α‐amylase and α‐glucosidase. Corosolic acid was selected based on favorable docking scores, binding interactions, and low root‐mean‐square deviation and was subjected to molecular dynamics simulations, which confirmed the stability of the resulting complex. This study concludes that reduced graphene oxide nanosheets derived from *C. igneus* leaves represent a novel antidiabetic agent that can reduce blood glucose levels and mitigate the complications associated with Type II diabetes.

## 1. Introduction

The worldwide incidence of Type 2 diabetes mellitus (T2DM) is escalating rapidly, accounting for 90%–95% of all diabetes cases globally. This tendency is primarily influenced by alterations in lifestyle and socioeconomic factors, including diminished physical activity and heightened intake of high‐fat, high‐carbohydrate meals [1]. Hyperglycemia is a fundamental characteristic of T2DM and significantly contributes to the majority of the disease’s pathogenic processes. It transpires when insulin sensitivity diminishes or insulin secretion from pancreatic β‐cells is compromised, which can subsequently inhibit insulin production and restrict glucose uptake in peripheral tissues. An essential approach to manage hyperglycemia entails the carbohydrate digestive enzymes inhibition, i.e., α‐amylase and α‐glucosidase, which are also pivotal in averting diabetes complications. Enzyme inhibitors impede carbohydrate breakdown, thereby diminishing glucose absorption from the small intestine and decreasing postprandial blood glucose levels. Therefore, the treatment and management of hyperglycemia and T2DM depend heavily on the repression of α‐amylase and α‐glucosidase [2, 3]. Currently, numerous commonly prescribed amylolytic enzyme blockers, including acarbose, miglitol, and voglibose, are available; nonetheless, these pharmaceuticals are linked to adverse effects including flatulence, diarrhea, and abdominal discomfort, which may be intolerable for patients. This underscores the urgent necessity for the creation of novel alternatives. Enhanced therapeutic outcomes may be attained with medicines that specifically block α‐amylase and α‐glucosidase, ensuring nontoxicity to target cells while efficiently diminishing postprandial hyperglycemia. Ideally, substances derived from natural sources like medicinal plants would show substantial repression of α‐glucosidase and modest repression of α‐amylase [4]. The integration of plant‐based alternatives in medicine is influenced by various critical aspects, including their therapeutic efficacy, cost‐effectiveness, and the growing inclination toward natural therapies. These reasons significantly contribute to the increasing incorporation of herbal medicines into conventional healthcare systems [5, 6]. Notwithstanding these advantages, obstacles remain, particularly the necessity for comprehensive scientific validation and the standardization of herbal products to ensure their safety and efficacy. Certain plants possess potentially poisonous or carcinogenic compounds, making them inappropriate for medicinal use. Consequently, it is vital to perform thorough investigations into their cytotoxicity to validate their safety and to endorse the ongoing utilization of these therapeutic plants. The utilization of biological components and plant extracts to transform inorganic compounds into nanoparticles over the past 3 decades has attracted a lot of interest [7].

Graphene is a monolayer‐thick honeycomb lattice of highly arranged sp2‐hybridized carbon atoms in six‐membered rings. This material boasts amazing features, including a huge surface area (∼2600 m^2^/g) [8], light weight [9], chemical stability [10], and strong electron mobility (200,000 cm^2^·V^−1^·s^−1^) [11], good mechanical and optical properties [12], good transparency, good impermeability [13], and good photocatalytic activity [14]. The synthesis of graphene has been accomplished by a number of novel synthetic processes. The procedures include chemicals like NaBH_4_ or hydrazine, which are extremely hazardous and can cause side effects in trace amounts. There are a lot of biological and environmental uses for rGO; however, this prevents them from being used. Therefore, there has to be continuous work to develop a method for synthesizing rGO that is safe for the environment and uses a nontoxic reductant [15]. Graphene researchers are currently focusing on this process, which is known as biosynthesis or green synthesis. A literature review indicates that rGO has been synthesized utilizing an extensive array of naturally produced materials, including vitamin C [16], saccharides [17], carrot roots [18], amino acids [19, 20], and polyphenols from tea [21]. In light of these methodologies, a straightforward and practical approach to synthesize rGO with *Costus igneus* leaf extract as the reducing agent was done.

**Figure 1:**
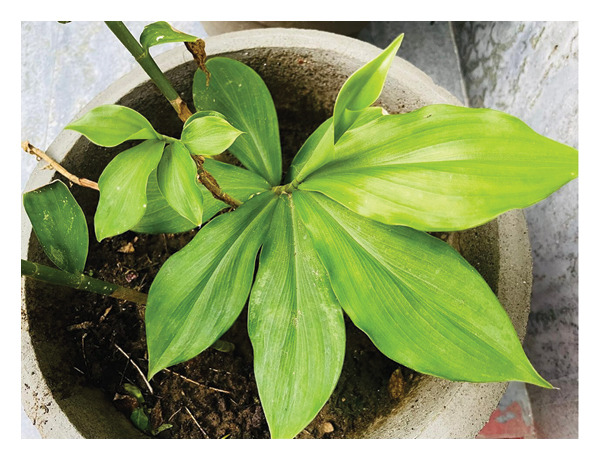
*Costus igneus* plant.

A member of the family Costaceae, *C. igneus* (Figure 1) goes by several names, including fiery *costus*, step ladder, and spiral flag. This plant originally hails from Central and South America. It is a tall, spreading perennial with spirally arranged leaves and the tallest stems measuring around two feet. Its leaves are used by indigenous people in India to control diabetes, and it is widely grown in gardens [22]. The phytochemicals present in plants have been the subject of much research due to their importance in traditional medicine. These phytochemicals include alkaloids, flavonoids, saponins, tannins, terpenoids, glycosides, and phenolic compounds [23, 24].

Nanoscience, characterized by its diminutive scale, capacity to facilitate drug delivery across biomembranes, and biocompatibility, has recently garnered significant interest in antidiabetic research. Notwithstanding the extensive application of this plant in traditional medicine, its biological and pharmacological properties remain largely unknown. This study investigates the antidiabetic efficacy of *C. igneus* leaf extract and its associated rGO nanosheets through in vitro, kinetic, and in silico models.

## 2. Materials and Methods

### 2.1. Acquisition of Plant Material

From Kanha Shanti Vanam, Hyderabad, India, leaves of the *C. igneus* plant were acquired. The plant leaves have been found and confirmed by former Chief Scientist Dr. Sunita Garg and Head of the Raw Material Herbarium and Museum at CSIR‐NIScPR, Delhi, Mr. R.S. Jayasomu.

### 2.2. Elicitation of Phytochemicals

A slightly altered version of the conventional method described for other leaf extracts was used to prepare the *C. igneus* leaf aqueous extract (Aq‐CI) [25, 26]. The leaves were rinsed under a faucet to eradicate any dust or contaminants postharvest. The pristine leaves were desiccated in the shade for 21 days. The desiccated leaves were ground in an electric grinder, and the resultant fine powder was utilized for extract preparation. Ten grams of *C. igneus* powder was placed in a beaker, and one hundred milliliters of Milli‐Q water was added. The maceration process was conducted, during which the mixture was continually mixed for 3 days. The solution was filtered through muslin cloth and subsequently through Whatman filter paper to eliminate residue. For the subsequent analysis, the concentrated filtrate was freeze‐dried after being produced using a rotary evaporator. The resulting concentrated Aq‐CI was then preserved at 4°C. The Aq‐CI was analyzed and revealed the presence of various phytochemicals: Tannins were identified with the FeCl_3_ test, glycosides were identified through the Legal test, alkaloids were identified using Dragendorff’s test, flavonoids were identified by Shinoda’s test, saponins through the vortex method, proteins through the biuret test, phytosterols with concentrated H_2_SO_4_, polyphenols using FeCl_3_ and K_3_[Fe(CN)_6_], carbohydrates via the Molisch test, and amino acids via the ninhydrin test.

### 2.3. Synthesis of Graphene Oxide (GO)

A few tweaks to Hummer’s method were employed to produce GO from graphite powder [27]. By mixing 30 mL of sulfuric acid with 10 g of potassium permanganate for half an hour at 20°C, a solution was created that contained 2 g of graphite flakes and 1 g of sodium nitrate. The mixture was then stirred for 3 h at 25°C. Subsequent to the addition of 150 mL of Milli‐Q water dropwise to the solution at 40°C, 30% hydrogen peroxide was added, and then, the mixture was aggressively stirred using a magnetic stirrer. Centrifugation at 3000 rpm for 10 min (three times) followed by rinsing with Milli‐Q water and ethanol until the pH was almost neutral. The GO powder was subsequently heated in an oven to dry it.

### 2.4. Reduced GO (rGO) Nanosheet Biosynthesis

Ultrasonication was applied to a solution containing 0.04 g of GO powder and 50 mL of Milli‐Q water for half an hour. Mixture of 40 mL of 0.8 mg/mL GO solution and 80 mL of 5% *C. igneus* leaf extract (reducing agent) was refluxed for 48 h at 24°C [28]. After rinsing with Milli‐Q water and ethanol by centrifugation at 10,000 rpm for 10 min (three times) each, the resulting black powder was allowed to dry at room temperature. The preparation technique for GO and *C. igneus*–derived rGO (CIrGO) is delineated in Figure 2.

Figure 2Schematic representation of the green synthesis of rGO using *Costus igneus* leaf extract. (a) Preparation of graphene oxide (GO) using a modified Hummers’ method: Graphite is mixed with NaNO_3_ and H_2_SO_4_, followed by KMnO_4_ oxidation, reaction termination with H_2_O_2_, and subsequent washing and drying to obtain GO. (b) Green reduction of GO: Aqueous *Costus igneus* leaf extract is used as a natural reducing agent. GO is refluxed, followed by centrifugation and drying to obtain CIrGO.

**Figure:**
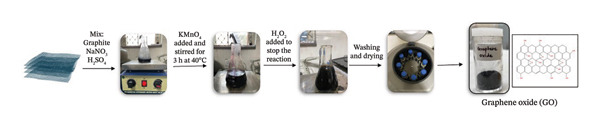
(a)

**Figure:**
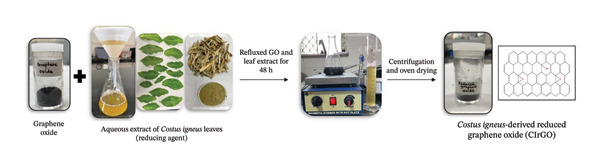
(b)

### 2.5. Structural and Morphological Characterization

The SHIMADZU spectrophotometer (Linco) was used to find the UV–vis spectra, which allowed us to determine the structural parameters of Aq‐CI, GO, and CIrGO. The spectral range of the instrument falls between 190 and 900 nm, and it has a precision of ±0.3 nm. Using Fourier‐transform infrared (FTIR) spectroscopy (Nicolet iS50 equipment from THERMO Electron Scientific Instruments LLC FTIR), the surface functional groups of Aq‐CI, GO, and CIrGO were analyzed. Spectra were obtained with an FTIR spectrophotometer having a spectral resolution of 4 cm^−1^, a step size of 1 s, and an infrared wavelength range of 4000–500 cm^−1^. A PANalytical PRO fitted with a radiation source of CuKα (*λ* = 1.5418 Å) piqued X‐ray diffraction (XRD) were examined. Diffraction intensities were logged at each 2*θ* angle ranging from 5° to 80°. The diffraction patterns were generated with a step size of 0.02°, and the scan rate was 2°/min. A JEOL 2100F transmission electron microscope (TEM) was employed to investigate the underlying structures of the gathered sheets operating at 200 kV, and SEM‐EDX (Zeiss EVO40, Carl Zeiss microscopy, coupled with Team Pegasus EDS‐EBSD, combining Octane Plus and Hikari Pro) showed the surface pictures and elemental composition percentages of GO and CIrGO. Spectra of Raman were recorded by means of a Varian FT‐Raman micro‐Raman spectrometer. In the range of 200–3500 cm^−1^, the spectra were recorded. To determine whether GO and CIrGO were stable in colloidal solutions and to evaluate their hydrodynamic particle size distribution, a Malvern PANalytical Zetasizer potential analyzer was used.

### 2.6. Cell Culture

Human glioblastoma U87MG cells and human Chang liver cells, sourced from the National Centre for Cell Sciences in Pune, Maharashtra, were cultured in a mixture of DMEM (HiMedia) augmented with L‐glutamine, FBS, streptomycin, and penicillin and maintained in a 5% CO_2_ incubator at 37°C. Chang liver and U87MG cells are the commonly used human cell lines in biomedical research to study various metabolic activities, insulin response, and cancer metabolism.

### 2.7. Cytotoxicity Assessment

Using a cell viability experiment, the cytotoxicity of metformin, Aq‐CI extract, and CIrGO nanosheet was assessed. A total of 96‐well plates were utilized to culture Chang liver cells and U87MG cells, with 2.5 × 10^4^ cells per well. After that, the cells were placed in a CO_2_ incubator and left there for 24 h. The cells were treated to different concentrations of metformin, CIrGO nanosheets, and Aq‐CI extract (12.5–200 μg mL^−1^) in triplicate the next day to assess their cytotoxic effects. A vehicle control was established using DMSO. 3‐(4,5‐Dimethylthiazol‐2‐yl)‐2,5‐diphenyl tetrazolium bromide (MTT) dye was added and incubated for 4 h at 37°C in the dark [21], and100 μL DMSO (dimethyl sulfoxide) was added to the mixture, forming formazan crystals. The absorbance at 560 nm was measured with a BioTek microplate reader. To determine the percentage of viable cells, the following formula has been used:

(1) cell viability=absorbance sampleabsorbance control ×100.

### 2.8. In Vitro Investigation of Antidiabetic Efficacy

#### 2.8.1. Glucose Uptake Assay

Using a modified glucose oxidase/peroxidase method, the effects of the plant extract on glucose utilization were evaluated in the Chang liver and U87MG cells [29]. Following their 3 × 10^4^ cells/mL inoculation, the cells adhered to a 96‐well plate overnight. The cells were treated to different quantities of Aq‐CI extract and CIrGO nanosheets (12.5–200 μg·mL^−1^) throughout 24 h. After adding 25 μL of incubation buffer per the media instructions, cells were washed with PBS and the medium was discarded. Mixing 0.1% BSA‐containing PBS until glucose reached 8 mM yielded this buffer. The presence of metformin, at a concentration that may vary between 12.5 and 200 μg·mL^−1^, served as a positive control. Following 4 h of incubation, the plates underwent treatment with 200 μL of glucose oxidase/peroxidase reagent. The absorbance at 510 nm was measured using a BioTek microplate reader following 15 min of room temperature incubation for the reaction. Using the red‐hued quinoamine dye complex, a colorimetric glucose oxidase/peroxidase test assessed the glucose concentration in the culture medium at 510 nm over time. Controls were cell‐free wells with incubation buffer and nutrient medium. A correlation was determined and shown between the amount of glucose that was absorbed in millimoles,

(2) % glucose uptake=absorbance control−absorbance sampleabsorbance control×100.

To ensure that fluctuations in glucose uptake were not caused by changes in cell viability, the MTT experiment was performed. In the MTT assay, a soluble tetrazolium salt is converted into an insoluble formazan product. The first material is soluble in water and has a yellow hue. Purple formazan crystals have been dispersed in DMSO. Viable cells are steadily proportionate to formazan concentration.

#### 2.8.2. Inhibition Assay of Amylolytic Enzymes

##### 2.8.2.1. α‐Amylase Inhibition Assay

To inhibit pancreatic α‐amylase activity, samples were tested in a mixture of 100 μL of a suspension (2 U/mL) in PBS, 100 μL of each Aq‐CI extract, CIrGO, and acarbose (a positive control) at concentrations from 12.5 to 200 μg·mL^−1^. After a 10‐min shaking bath incubation at 37°C, 1% starch solution was added to the mixture to act as a substrate, and the incubation was proceeded for an additional 10 min. Samples were boiled in water at boiling point for 10 min after being mixed with dinitrosalicylic (DNS) acid color reagent, which contained 2 M sodium hydroxide, 5.3 M sodium potassium tartrate in 96 mM 3,5‐DNS acid in order to halt the reaction. Determined the inhibitory capacity at 540 nm after bringing it to room temperature using the following formula [30]:

(3) % inhibition=absorbance control−absorbance sampleabsorbance control×100,

where the average values of samples (Aq‐CI extract and CIrGO) or acarbose with enzyme are denoted by absorbance (sample), and the buffer with enzyme is denoted by sbsorbance (control).

##### 2.8.2.2. α‐Glucosidase Inhibition Assay

The inhibition of α‐glucosidase was evaluated using a recent work with minor modifications [31]. After mixing the samples that contained CIrGO and Aq‐CI extract with α‐glucosidase enzyme (phosphate buffer, 0.1 M, pH 6.9), the mixture was left to incubate for 10 min. Acarbose served as a positive control. 10 mM of 4‐nitrophenyl α‐D‐glucopyranoside was added. Two milliliters of 0.1 M Na_2_CO_3_ was added after 20 min of incubation to halt the reaction. The inhibition rate was determined by measuring absorbance at 405 nm and applying the following formula:

(4) % inhibition=absorbance control−absorbance sampleabsorbance control×100.

#### 2.8.3. Kinetic Study of Amylolytic Enzymes

##### 2.8.3.1. Kinetic Behavior of α‐Amylase With Inhibitors

The Michaelis–Menten and Lineweaver–Burk equations were employed to elucidate the mechanism by which alpha‐amylase is inhibited by CIrGO nanosheet and acarbose. Utilizing the IC_50_ values of the samples to examine enzyme kinetics. For the substrate, soluble starch was utilized at concentrations of 0.1%, 0.25%, 0.5%, 1%, and 2% w/v. The enzyme kinetics were evaluated in the absence and presence of CIrGO nanosheet and Aq‐CI extracts. A 30 min preincubation at 37°C was performed using 100 μL of an alpha‐amylase enzyme solution at a concentration of 2 U/mL for the purpose of summarization. Subsequently, differing volumes of starch solution (750 μL) were introduced and allowed to incubate at 37°C for a duration of 10 min. The implementation of the DNS reagent brought the process to a standstill. Lineweaver–Burk plots represent the relationship between the inverse of velocities (1/V) and substrate concentrations (1/mM^−1^), and absorbance variations at 405 nm were measured spectrophotometrically [32]. This enabled us to ascertain the type of enzyme inhibition. Both the intercept and slope values from the double inverse plots were utilized to get the kinetic constants Km and Vmax, which in turn led to the measurement of the reaction velocities (v). The concentration of reducing sugars was quantified utilizing a maltose standard curve, expressed in millimoles of maltose equivalent.

##### 2.8.3.2. Kinetic Behavior of α‐Glucosidase With Inhibitors

The kinetic behavior of α‐glucosidase was examined when inhibitors were present.

The α‐glucosidase enzyme (1 U/mL) was suspended at 37°C for 5 min before using inhibitors (CIrGO nanosheets and acarbose, individually). A 100 mM phosphate buffer was used to dissolve the enzyme. Various amounts of sucrose were added after the enzyme‐inhibitor mixture had been preincubated at 37°C for 30 min. Data were evaluated to ascertain a category of inhibition (competitive, uncompetitive, noncompetitive, or mixed) using Lineweaver–Burk plots, which were derived from the results obtained using Michaelis–Menten kinetics. Plotting the reciprocal values of reaction velocities (*V*_*o*_) and substrate concentrations (S) and fitting them to the Lineweaver–Burk equation allowed us to determine the maximum reaction velocity (*V*_max_) and the Michaelis constant (*K*_*m*_) [32].

The Lineweaver–Burk equation (double reciprocal plot) is given as

(5) Vo=Vmax SKm+S,

(6) 1Vo=Km +SVmaxS=KmVmax1S+1Vmax.

### 2.9. In Silico Analysis of Antidiabetic Efficacy

#### 2.9.1. Ligand Selection and Protein Structure Acquisition

Scopus, Web of Science, PubMed, and Google Scholar were utilized to search for articles pertaining to *C. igneus* leaves and extract their chemical makeup. Consequently, PubChem databases were used to create a database of bioactive chemicals discovered in the leaves of *C. igneus*. These compounds were identified using induced‐fit docking techniques with the Ligand Preparation Wizard of Maestro 11.1 [33] combined with an OPLS3 force field. Refining the structures involved bringing the protein’s pH to a neutral level and removing water molecules. The α‐amylase and α‐glucosidase crystal structures were retrieved from the Protein Data Bank with the respective accession numbers of 1DHK and 4J5T. To come up with the optimal receptor, Chimera (Amber Force Field) in conjunction with SWISS‐PDB Viewer’s GROMACS 96 43B1 algorithm was used. Thiol and hydroxyl groups, amide groups of asparagine and glutamine, histidine’s imidazole ring, protonation states of histidine, aspartic acid, and glutamic acid were among the reformation products [34].

#### 2.9.2. ADME/T Analysis and Drug‐Likeliness Predictions

The toxicity was evaluated using pkCSM, and the ligand physicochemical characteristics were predicted using QikProp and SwissADME web server. Various criteria were employed to assess the compounds, including oral absorption rate, QlogP, and Lipinski’s rule of five [35].

#### 2.9.3. Grid Generation and Molecular Docking

Previous studies [36, 37] employed similar modeling methods to the one used in this research, which involved the α‐amylase and α‐glucosidase enzymes (PDB codes: 1DHK and 4J5T, respectively). The protein structure was created and refined using the Schrödinger software suite. For crystallographic analysis, water molecules were removed, along with any heteroatoms or contaminants. Bond orders were assigned based on the presence or absence of hydrogens. Several issues, including errors in formal charges, side chains, and backbone chains, were addressed and corrected. The structure’s resonance, ionized states, ring arrangements, and tautomers were examined utilizing the LigPrep tool. A standard precision docking approach, employing the default OPLS 2005 Force Field, was applied to calculate docking scores and assess ligand‐binding affinities in detail.

#### 2.9.4. Molecular Dynamics Modeling

The stability of the lead compounds in conjunction with the 1DHK and 4J5T proteins was assessed through molecular dynamics (MD) simulations, based on methodologies outlined in previous studies [38, 39]. The Desmond tool was employed for the simulations. All computational tasks were carried out using 4GB NVIDIA Quadro, running on Ubuntu 18.04. The protein–ligand complex was generated in Glide software and subsequently imported to Schrödinger for further manipulation. The complex was centered in an orthorhombic box. Counter ions neutralized charges, and water molecules solvated the system. The Desmond system simulated physiological conditions with 0.15 M NaCl. Energy minimization was performed using the OPLS‐3e force field to resolve any electronic issues within the protein structures. At 298 K and 1 bar pressure, the NPT array performed a 100‐ns simulation run with 1000 steps. The barostat algorithm‐controlled pressure and temperature. Analysis of MD trajectories and prediction of ligand‐binding layout in complexes was done using Desmond’s modeling interface model.

### 2.10. Statistical Analysis

Data were analyzed using GraphPad Prism. The results from all biological studies, triplicates shown as Mean ± SEM. The significance was verified by one‐way ANOVA and Tukey’s post hoc test. OriginPro created structural characterization graphical representations.

## 3. Result

### 3.1. Biosynthesis of CIrGO

A green approach was established to fabricate CIrGO nanosheets via its generic counterpart, GO, using *C. igneus* leaf extract as a reducing agent. The leaf extract can be easily and cheaply prepared. The production of CIrGO was confirmed by all spectroscopic investigations, and the results are detailed in this article.

#### 3.1.1. Phytochemical Screening

Phytochemicals were detected in both the synthesized CIrGO and the Aq‐CI during the preliminary qualitative screening. The phytochemicals found in the *C. igneus* extract included alkaloids, carbs, phenol, protein, saponin, and terpenoids; in contrast, the CIrGO exhibited phenol, protein, and terpenoids as its phytochemical components. A variety of plant biomolecules, including tannins, alkaloids, proteins, and enzymes, stabilize and reduce GO. In this study, alkaloids, carbohydrates, phenols, proteins, saponins, and terpenoids were all detected in the *C. igneus* extract. Translocation of metabolites such as phenols, terpenoids, and proteins from plant residues to rGO nanosheet during fabrication was also shown. These compounds likely reduced and stabilized synthesis. Studies on the screening of phytoconstituents of medicinal plants employed for rGO synthesis found that these phytochemicals capped and steadied the green synthesis [40–43].

#### 3.1.2. Structural Characterization and Morphology

##### 3.1.2.1. UV–Visible Spectra Analysis

**Figure 3:**
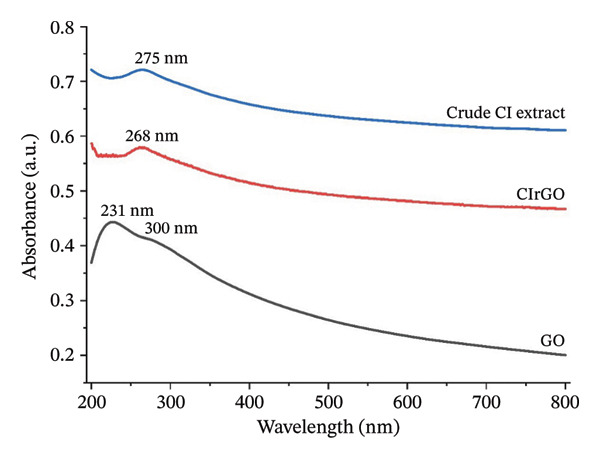
UV–vis spectrum of crude, GO, and CIrGO aqueous suspension.

Analyzing the change in the UV–visible absorption spectra of CIrGO over time allowed us to study the GO reduction process (Figure 3). A high absorption peak at 231 nm was observed in the aromatic C–C bonds (п–п transition) in GO, as revealed by the UV analysis. A weak shoulder at 300 nm was observed due to the n–п transitions in the carbon–oxygen bonds. With the completion of the reduction process, a new peak appeared and the characteristic peak shifted from 231 to 268 nm (Figure 3), confirming the restoration of electronic conjugation in the graphene sheets. The extent of the red shift observed can be used to evaluate the effectiveness of the reducing agent [44]. To illustrate the efficacy of the polyphenols found in the *C. igneus* extract as reducing agents, we observed that CIrGO with an absorption maximum at 268 nm, surpassing 254 nm observed when hydrazine [45] and phenyl hydrazine [46] reduced graphene, respectively. One possible explanation for the characteristic broad shoulder is that graphene nanosheets have alkaloids and polyphenols bonded to their surfaces, which inhibit graphene aggregation. Aq‐CI exhibits notable UV–vis absorption peaks, generally around the CIrGO peak at approximately 275 nm [47].

##### 3.1.2.2. FTIR Study

**Figure 4:**
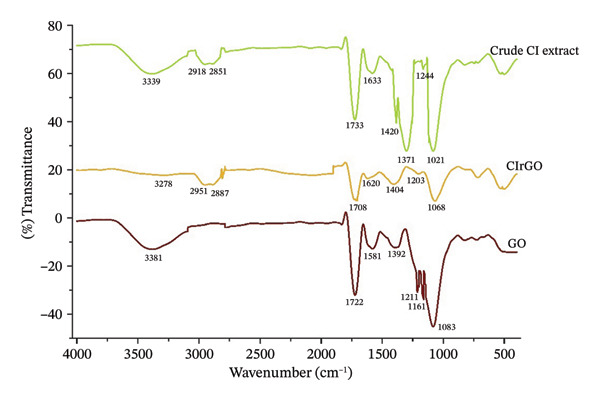
FTIR spectra of crude extract, GO, and CIrGO samples.

Figure 4 shows the results of FTIR spectroscopy tests conducted on the GO and CIrGO samples, which were used to determine the level of reduction. O–H stretching vibrations seen in GO as a wide band in the 3100‐3400 cm^−1^ range. The carbonyl group’s C=O stretch causes an absorption band at around 1722 cm^−1^, whereas vibrations in the epoxide ring and the O–H bending give rise to a band at about 1581 cm^−1^. GO’s surface carboxylic (COOH) groups narrow the absorption resonance approximately 1083 cm^−1^ by causing the carbon–oxygen stretching resonance peak. A third‐order alcohol (C–OH) molecule might be responsible for the absorption band that centers at 1392 cm^−1^. GO’s oxygen functionalities, the O–H bonds, C=O groups, carbon oxide, and intermediate C–OH bands, significantly reduced in the FTIR spectra of CIrGO, as much as they were in graphene made from plant extracts. The spectra that were detected provide more evidence of this deoxygenation or decrease in the oxygen concentration. Furthermore, the FTIR bands observed for the crude *C. igneus* extract appearing at 3339, 2918, 2851, 1733, 1633, 1420, 1244, and 1021 cm^−1^ showed good agreement with those found in the CIrGO, supporting the involvement of phytochemicals in the reduction and stabilization of the graphene sheets [48].

##### 3.1.2.3. Raman Analysis

**Figure 5:**
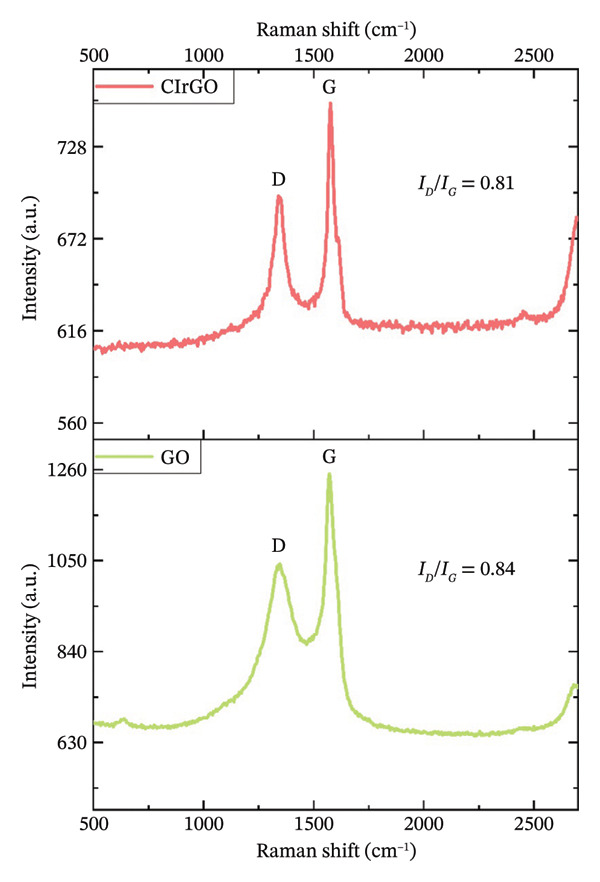
Raman peak of GO and CIrGO nanosheets.

GO and CIrGO’s D (disordered mode) and G (in‐plane vibration of sp^2^ bonded carbon atoms) bands were seen by Raman spectroscopy (Figure 5). The Raman absorption spectrum of GO and CIrGO revealed clear D and G bands at 1347 and 1339 cm^−1^ and 1571 and 1575 cm^−1^, respectively, confirming their presence. The ID/IG ratio, which is the intensity ratio of the D band to the G band, was computed for GO and CIrGO to understand the reduction process, specifically oxygen removal from graphene. The increased adsorption of oxidized polyphenols on the graphene surface increases surface sp^3^ domains and the ID/IG ratio following reduction. Initially, GO had an ID/IG ratio of 0.84. The ID/IG ratio for CIrGO was 0.81 after reduction, similar to the CIrGO intensity values of earlier research [47, 49].

##### 3.1.2.4. XRD Spectral Analysis

**Figure 6:**
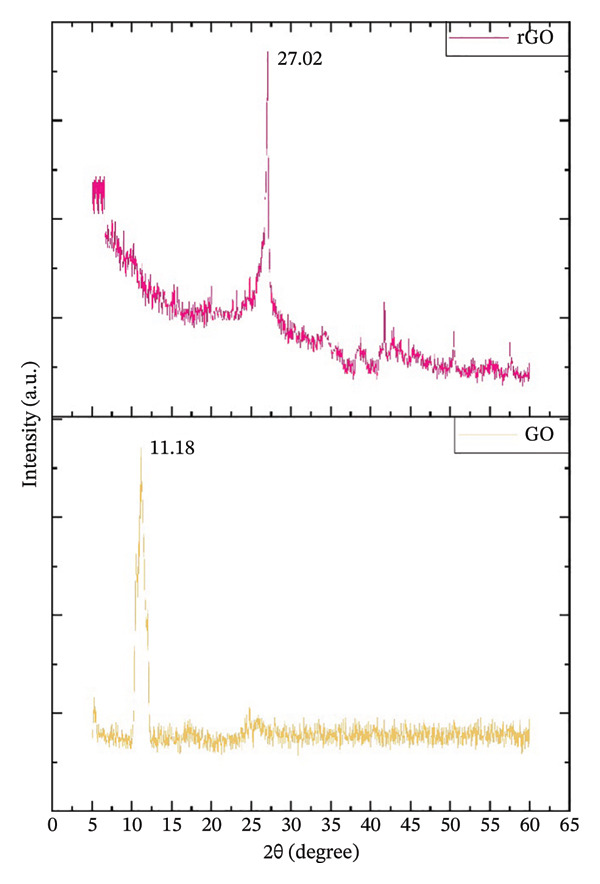
X‐ray diffraction of GO and CIrGO nanosheets.

GO and CIrGO XRD diffractograms are illustrated in Figure 6. Oxidation of graphite layers revealed a single diffraction signal at 11.18°, indicating water intercalation and carboxylic bonds with an intermediate d‐spacing of 0.76 nm. CIrGO showed a large dispersion peak at 2*θ* = 27.02° after reduction, with a 0.33‐nm interlayer d‐spacing. The Bragg reflection planes for GO were identified as (002), while for CIrGO, they were (111). The observed diffraction patterns were consistent with those of GO and rGO synthesized using the leaf extract of *C. igneus*. The full elimination of the oxygen group presence in GO following reduction was confirmed by the moderately lower d‐spacing value and the disappearance of the peak at 11.18° [48, 49]. The following formula can be used to find the d‐spacing:

(7) d=nλ2sinθ,

where *n* is the integer (assuming to be 1), *λ* is thewavelength of the incident X‐rays, and 2*θ* is the diffraction angle.

##### 3.1.2.5. Dynamic Light Scattering and Zeta Potential

The zeta potential of CIrGO and GO is shown in Figures 7(a) and 7(b). Surface charges of −24.8 and −22.9 mV, respectively, were recorded for GO and CIrGO. It was clear from those negative numbers that GO and rGO sheets were stable. GO may have a high negative zeta potential due to its carboxyl structural groups. A value of −22.9 mV is obtained following GO reduction with *C. igneus* leaves. This is because GO surfaces have had their carboxyl groups removed. Figures 7(c), 7(d) depict dynamic light scattering evaluations of GO and CIrGO. The dispersion size of CIrGO in an aqueous solution was found to be 79.82 nm, while GO measured 80.89 nm, indicating that CIrGO is slightly smaller. According to previous investigations on rGO [48], a reduction in the particle dimension was associated with the absence of long‐range graphitic order. Cavitation at the rGO nanosheet surface using ultrasonication weakens the influence of van der Waals forces, limiting clumping. This stabilizes rGO in aqueous solutions, resulting in well‐dispersed, smaller fragments.

Figure 7Zeta potential of GO (a) and CIrGO (b) and dynamic light scattering spectra of GO (c) and CIrGO nanosheets (d).

**Figure:**
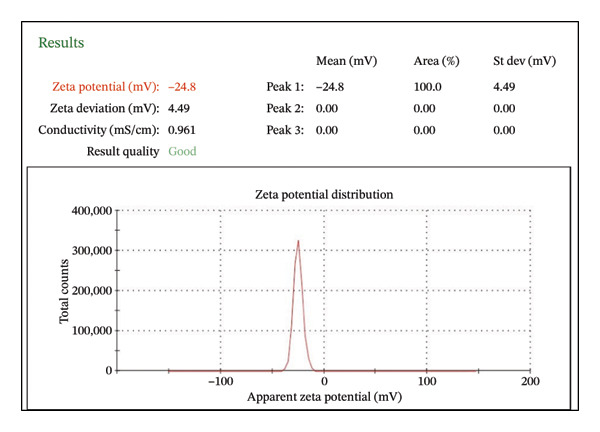
(a)

**Figure:**
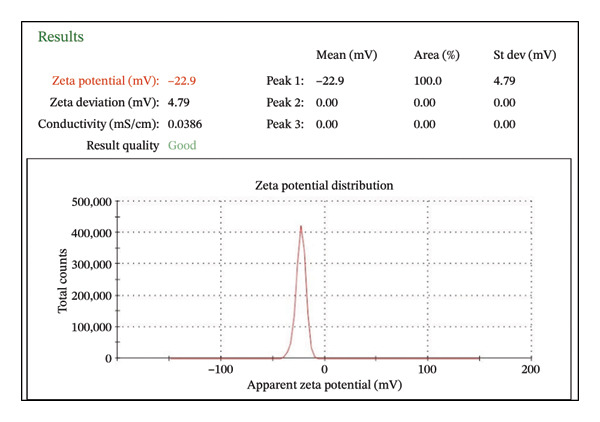
(b)

**Figure:**
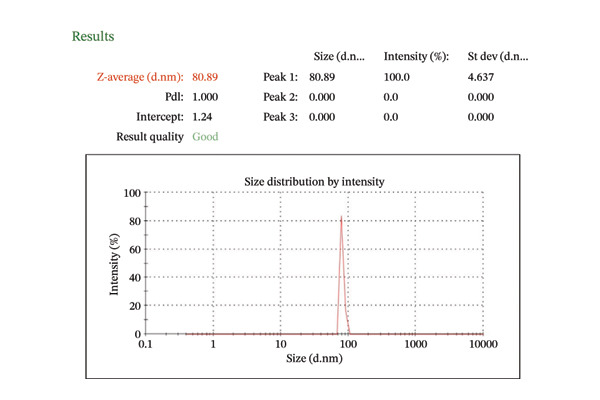
(c)

**Figure:**
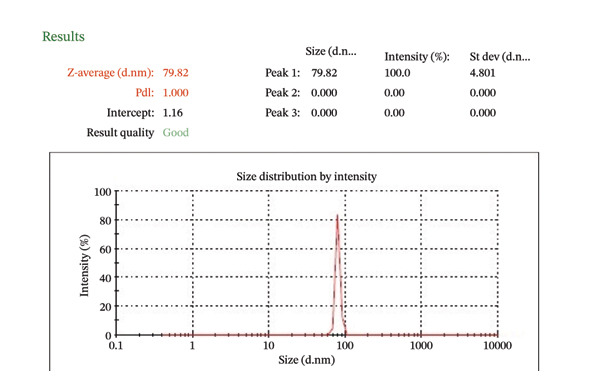
(d)

##### 3.1.2.6. Morphological Analysis

Figure 8 displays the chemical makeup and shape of rGO produced from the *C. igneus* leaf extract. Scanning electron micrographs of GO (Figure 8(a)) and CIrGO (Figure 8(b)) revealed thin nanosheet structures. At the same time, substantial signals for elemental C (61.94%) and O (23.98%) were observed in an EDAX spectrum that was acquired to validate the chemical composition of CIrGO. Table 1 shows the carbon‐ and oxygen‐containing groups in CIrGO EDAX data. Figure 8(c) shows the outcomes of the CIrGO morphology study by TEM. The CIrGO appeared as thin, edge‐fragmented sheets upon initial inspection. An arrangement of the thinly layered CIrGO nanosheet is also used to border the boundaries [47].

Figure 8SEM image of GO (a) and CIrGO (b) and TEM image of GO (c) and CIrGO nanosheets (d).

**Figure:**
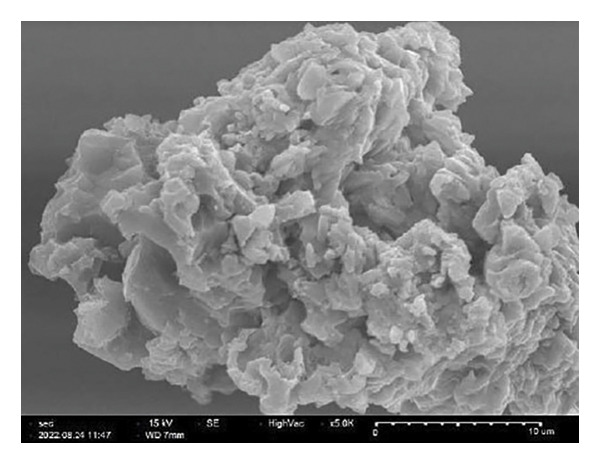
(a)

**Figure:**
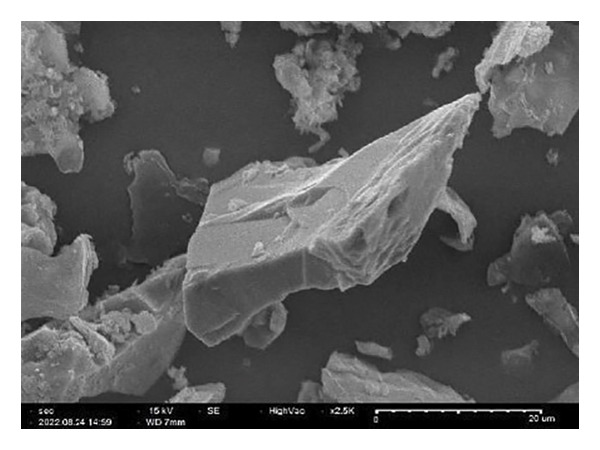
(b)

**Figure:**
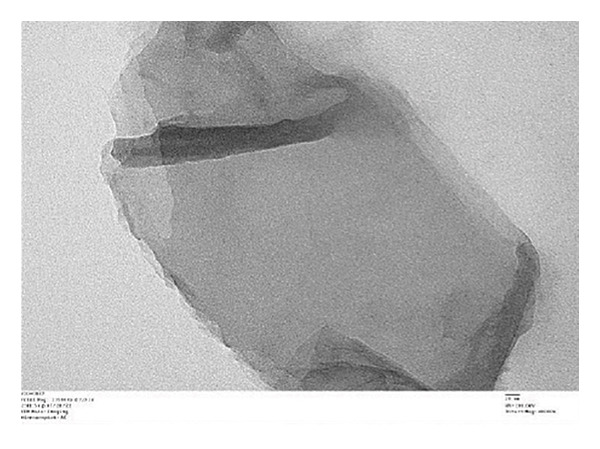
(c)

**Figure:**
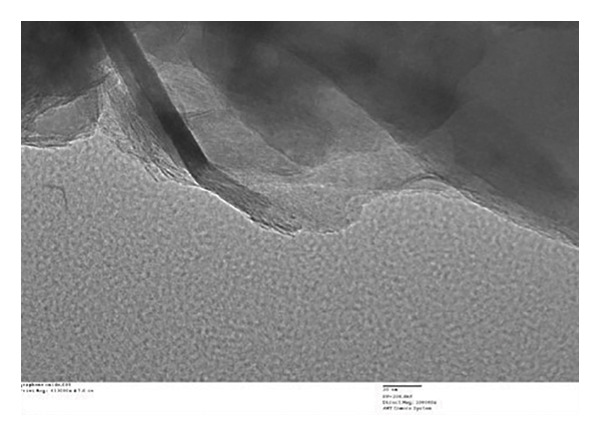
(d)

**Table 1:** EDAX elemental composition of GO and CIrGO samples, showing carbon, oxygen percentages, and C/O ratio.

Sample	C%	O%	C/O
GO	57.34	32.5	1.76
CIrGO	61.94	23.98	2.58

#### 3.1.3. Plausible Reduction Mechanism

**Figure 9:**
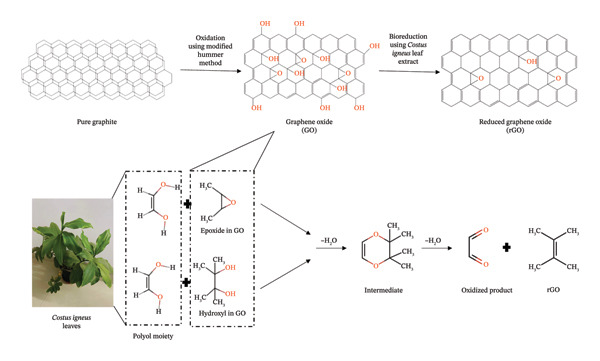
An approach for the reduction of graphene oxide: Polyol‐rich phytochemicals react with epoxide and hydroxyl groups in GO, forming intermediates and removing oxygen functionalities, leading to the restoration of the graphitic rGO structure.

The transition in hue from brownish yellow (GO) to black (CIrGO) served as the most consistent signal of the successful transformation of GO to CIrGO. Aq‐CI contains a variety of phytochemicals that may act as reducing agents, including glycosides, sugars, alkaloids, saponins, flavonoids, phytosterols, tannins, phenolic compounds, proteins, and amino acids [25, 26]. Each of these components’ testing methods verified their presence in the extract. The green production of rGO has been made possible by the identification and comprehensive characterization of individual molecules whose reduction pathways were excellent stabilizing agents. These substances include sugars, proteins, amino acids, and polyphenols [50–52]. Figure 9 shows the potential method by which the *C. igneus* extract reduces GO. Epoxide, hydroxyl, and carbonyl are just a few of the oxygen‐containing functional groups found on the GO surface. It is possible to convert the epoxide and carbonyl groups to hydroxyl groups under basic circumstances. The Aq‐CI contains several polyphenols with extremely acidic hydrogen atoms that readily dissociate into their anionic forms. Nucleophilic substitution interactions between anionic polyphenols and GO’s hydroxyl group yield an intermediate [52]. The removal of a water molecule transforms the intermediate into rGO, referred to as CIrGO.

### 3.2. Cytotoxicity Assay

The toxicity of the produced Aq‐CI extract and CIrGO at different concentrations was assessed against two cell lines (Chang liver and U87MG) with the MTT test. Various concentrations from 12.5 to 400 μg·mL^−1^ of the Aq‐CI extract, CIrGO, and the positive control metformin were utilized. Assessing the compound’s toxicity is the initial phase in the therapeutic development process for a specific ailment. The findings indicated that the amalgamated CIrGO exhibited reduced toxicity, with cell mortality happening in a dose‐sensitive fashion. As the amount of CIrGO escalated, the toxicity level correspondingly increased, as depicted in Figure 10. Chang liver cells showed a dose‐dependent reaction to the evaluated substances. At a dosage of 12.5 μg·mL^−1^, viability of cells was measured at 92.6% for the Aq‐CI extract, 84.5% for CIrGO, and 95.12% for metformin, establishing the baseline for comparison. Treatment with 25 μg ·mL^−1^ led to a marginal reduction in cell viability to 82.79%, 7.13%, and 88.62% for Aq‐CI extract, CIrGO, and metformin, respectively, signifying negligible cytotoxicity. With increasing concentration, cell viability progressively diminished, exhibiting notable cytotoxic effects at 400 μg· mL^−1^ (33.19% for the Aq‐CI extract, 28.26% for CIrGO, and 38.13% for metformin), with total cell loss likely occurring at higher concentrations. The results demonstrate a pronounced concentration‐dependent cytotoxic effect of the examined substances. The inhibitory concentration (IC_50_) of CIrGO was found to be 235.72 μg·mL^−1^, while IC_50_ for the Aq‐CI extract was 303.89 μg·mL^−1^. In comparison, the positive control, metformin, exhibited an IC_50_ of 327.01 μg·mL^−1^. At higher concentrations, CIrGO may generate reactive oxygen species, leading to damage to cellular components and ultimately causing cell death.

Figure 10Cell viability assays were conducted using MTT. (a) The viability of Chang liver cells in response to CIrGO showed a dose‐dependent decline as the concentration increased. (b) U87MG cell viability also decreased significantly at higher concentrations of CIrGO. The bars denote the mean ± SEM (^∗∗^signifies a significant level compared to control (*p* ≤ 0.001) and ^∗∗∗^signifies a level of significance compared to control (*p* ≤ 0.0001), relative to untreated cells, *n* = 3).

**Figure:**
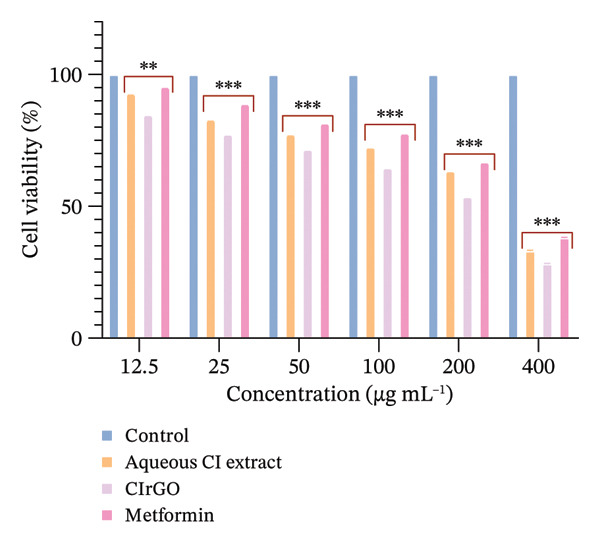
(a)

**Figure:**
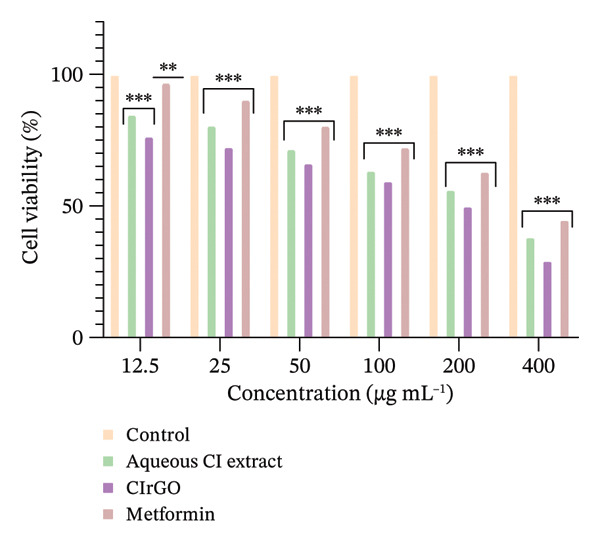
(b)

The response of U87MG cells to CIrGO exhibited a dose‐dependent effect. Beginning with a baseline viability of 76.425% at 12.5 μg ·mL^−1^, a minor decrease to 72.43% is seen at 25 μg· mL^−1^. At 400 μg· mL^−1^, viability drastically declined to 29.4%, indicating considerable cytotoxicity. Elevated doses resulted in total cell mortality, demonstrating the significant anticancer efficacy of CIrGO. The IC_50_ of CIrGO was measured at 199.73 μg· mL^−1^, whereas the Aq‐CI extract exhibited an IC_50_ of 276.34 μg· mL^−1^, in contrast to the positive control metformin, which had an IC_50_ of 346.74 μg· mL^−1^ (Figure 11).

Figure 11IC_50_ values of the tested samples on (a) Chang liver cells and (b) U87MG cells. Values were calculated from concentration–response curves obtained from cell viability measurements. Data are presented as mean ± SEM (*n* = 3); ^∗∗∗^denotes the statistical significance compared with untreated control cells (*p* ≤ 0.0001).

**Figure:**
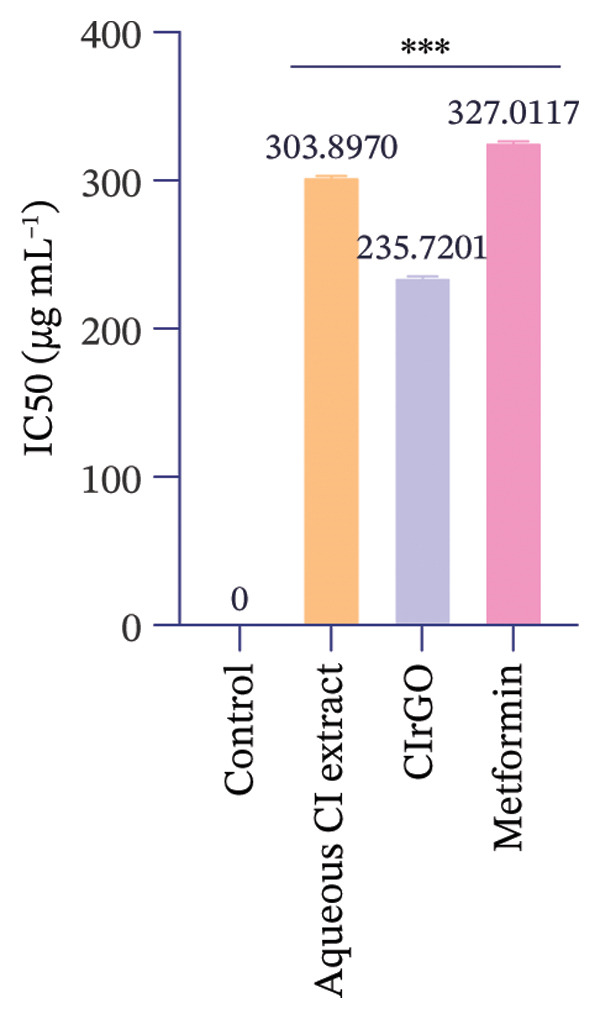
(a)

**Figure:**
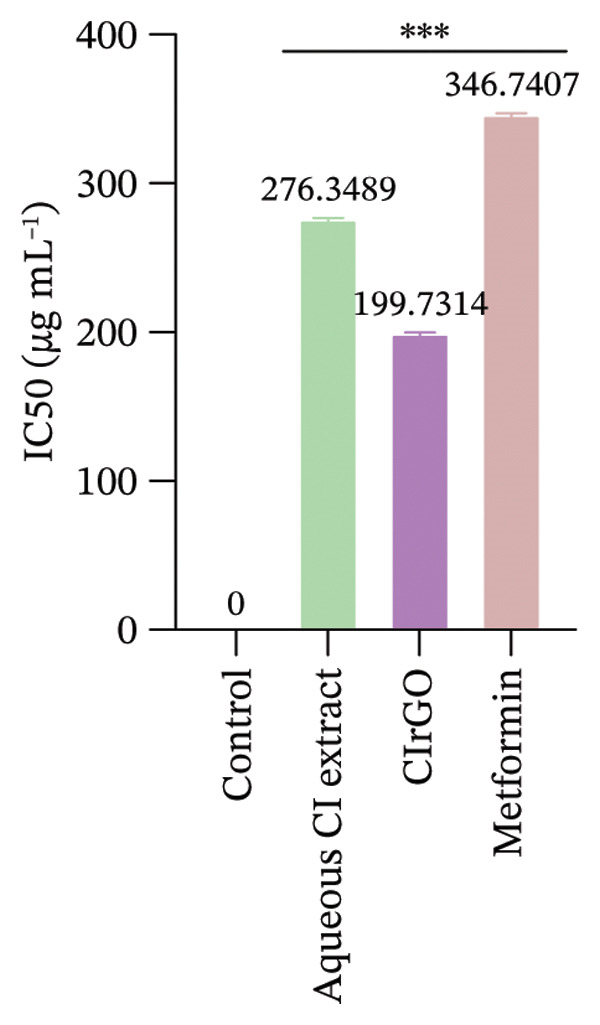
(b)

These findings closely correspond with other research that demonstrated analogous dose‐specific fatal repercussions of plant extracts on cells [53]. Furthermore, another study demonstrated that elevated levels of rGO diminish cell viability by eliciting oxidative damage, cell death, and rupture of the cell membrane. Higher doses of CIrGO treatments led to enhanced cytotoxicity in cancer cells, indicating that CIrGO may augment the generation of free radicals in these cells. CIrGO’s capacity to induce apoptosis, as demonstrated in these assays, aligns with our findings of the substantial cytotoxic impact on Chang liver and U87MG cells.

### 3.3. Glucose Uptake Assay

A lineage of proteins known as glucose transporters (GLUTs) facilitates the translocation of glucose across the exterior of cells. This rapid glucose transport pathway is crucial for the postprandial retention of insulin‐dependent glucose in adipose and muscle tissues, facilitating the oversight of blood glucose levels. At different doses, the graph shows how the GO nanosheets and water‐based extract of *C. igneus* (CIrGO) affected glucose absorption in Chang liver and U87MG cells. At higher tested values, CIrGO improved glucose absorption relative to the control, according to the results (Figure 12). The level of glucose absorption increased from 13.01% to 81.39% as the concentration of CIrGO in Chang liver cells increased from 12.5 to 200 μg· mL^−1^. A typical antidiabetic medicine, metformin, was used as a reference to assess the glucose absorption capability of CIrGO. The findings demonstrated that at a dosage of 50 μg ·mL^−1^, CIrGO displayed a glucose uptake capacity of 34.42%, which is comparable to metformin’s absorption rate of 38.56%. A 28.86% absorption rate is shown by the Aq‐CI extract. After successful clinical trials, Aq‐CI extract may replace metformin, the standard antidiabetic medicine, due to its similar absorption activity. After being administered to U87MG cells, CIrGO’s glucose absorption ability was also compared to that of metformin. The findings showed that at a dosage of 50 μg· mL^−1^, CIrGO had glucose uptake capacity of 22.74%, which is quite similar to metformin’s 30.31%. On the other hand, the Aq‐CI extract showed an uptake of 18.05%, which increased with stronger concentrations. The uptake activity of Chang liver cells was 81.39% when treated with CIrGO, which was significantly higher than U87MG cells’ 78.54%, and significantly different from the control (*p* < 0.0001). It has been found that the type of cell used in the experiment affects how well cells absorb glucose [54]. Yeast cells and other eukaryotic cells have quite different glucose uptake mechanisms. The rate of glucose metabolism and its intracellular concentration have a substantial impact on glucose absorption. Increased glucose absorption by the cell is promoted by a drop in the concentration of internal glucose caused by its transformation into other metabolites. These results suggest that enhanced glucose metabolism and improved diffusion may underlie the extract‐mediated glucose absorption by glioma cells and hepatocytes. Previous research indicates that insulin attaches to the tautomeric insulin receptor subunit on the myocyte cell membrane, initiating a cascade of events that facilitates the translocation of the GLUT 4 target to the membrane, thereby allowing glucose absorption into the cytosol [55, 56]. The primary mechanism by which polyphenols exert hypoglycemic effects is their ability to inhibit carbohydrate utilization in the intestine, modulate enzymatic glucose uptake, enhance β‐cell functionality and insulin efficacy, stimulate insulin secretion, and exhibit anti‐inflammatory and antioxidant capabilities.

Figure 12Glucose uptake assay: (a) Chang liver cells in response to CIrGO exhibited a dose‐dependent increase with escalating doses. (b) U87MG exhibited a notable enhancement at elevated concentrations of CIrGO. The bars denote the mean ± SEM (^∗∗^signifies a significant level compared to control (*p* ≤ 0.001) and ^∗∗∗^signifies a significance level compared to control (*p* ≤ 0.0001), *n* = 3).

**Figure:**
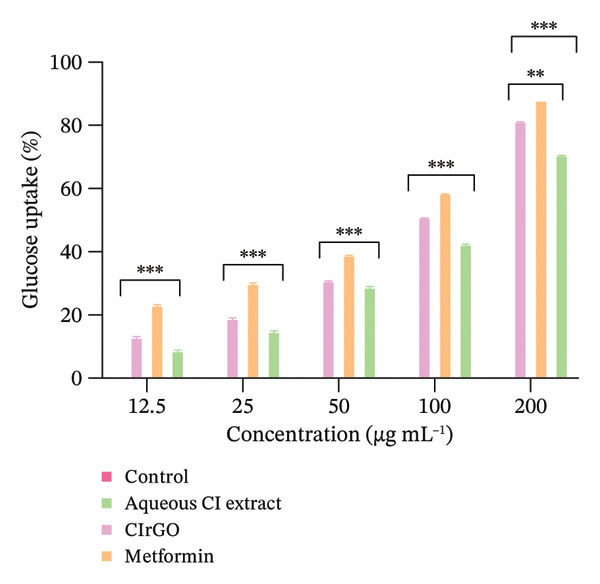
(a)

**Figure:**
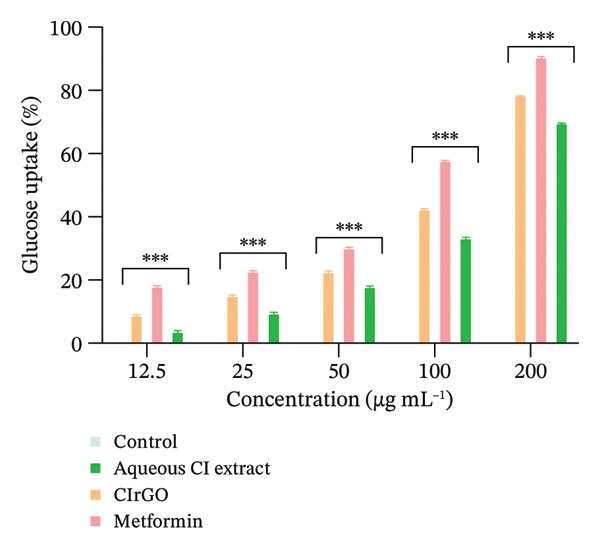
(b)

### 3.4. Amylolytic Enzymes Inhibition and Their Kinetic Study

#### 3.4.1. Inhibition Assay of α‐Amylase

Enzymes that break down carbohydrates, like α‐amylase in the pancreas and α‐glucosidase in the intestine, convert oligosaccharides and disaccharides into monosaccharides. This conversion leads to a significant increase in glucose production, which hampers its uptake by cells [57]. Inhibition of these enzymes leads to a reduced level of blood sugar in diabetic persons. Following incubation with different concentrations, the results demonstrated that both of these enzymes were markedly inhibited, with inhibition exhibiting a concentration‐dependent relationship. The phenolic chemicals found in abundance in the Aq‐CI extract served as stabilizing elements in the production of CIrGO [58]. Possible explanation for reduced enzyme activity is interactions between carbohydrate‐hydrolyzing enzyme’s amino acids and phytochemicals in the biosynthesized CIrGO. Molecular docking and interaction analysis were described later in the study to validate this relationship. At doses from 12.5 to 200 μg·mL^−1^, CIrGO showed a notable inhibition of α‐amylase. Compared to the control, the inhibitory effects of CIrGO increased dramatically (*p* < 0.0001) as the dosage increased. Inhibition at 100 and 200 μg·mL^−1^showed a notable increase. In comparison with the positive control acarbose, which had an IC_50_ of 65.46 μg·mL^−1^, the CIrGO exhibited an IC_50_ of 84.46 μg·mL^−1^ (Figure 13). A larger quantity of polyphenols is responsible for the improved inhibitory activities displayed by Aq‐CI, which had an IC_50_ of 92.62 μg·mL^−1^. Although there has been little research on the effects of naturally occurring rGO on alpha‐amylase inhibition, it has been demonstrated that the *C. igneus* plant’s leaves can considerably lower alpha‐amylase activity.

Figure 13Findings of inhibition of the α‐amylase investigation displayed, with data points illustrating the results of three autonomous experimental studies. The facts are presented as mean ± SEM. Values deemed substantially different (^∗∗^p < 0.001, ^∗∗∗^p < 0.0001) in comparison with the control at the same tested dosages.

**Figure:**
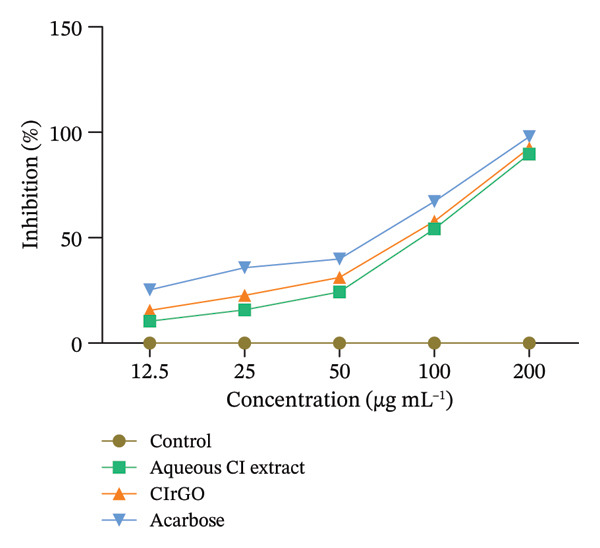
(a)

**Figure:**
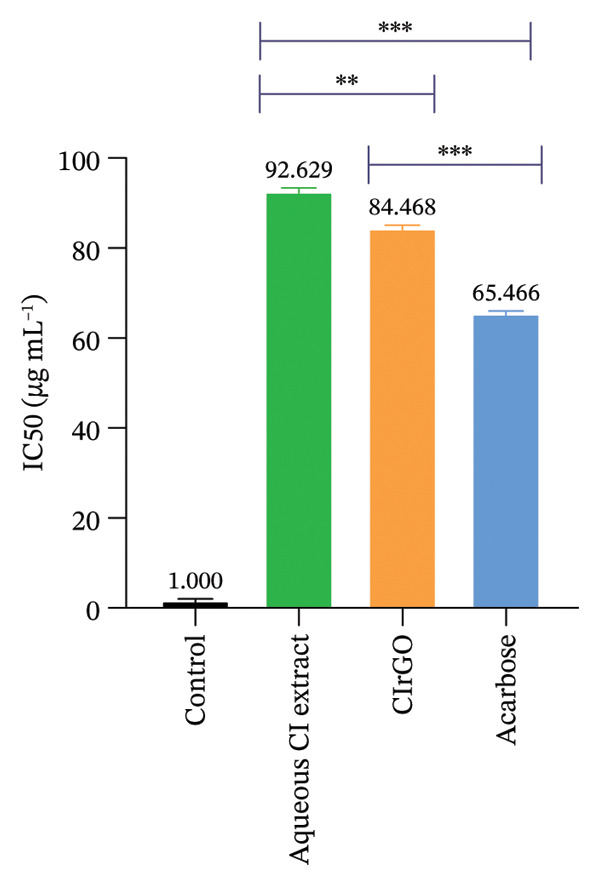
(b)

##### 3.4.1.1. Kinetics of Enzyme Inhibition

The kinetics of enzyme inhibition were examined using the Michaelis–Menten and Lineweaver–Burk plots for extracts at dosages aligned with their IC_50_ values. The reaction rate [V] (μmol sec^−1^) was quantified at different substrate levels, utilizing a graph calibrated with maltose. A graphical depiction (Figure 14) of the kinetic constant (*K*_*m*_ and *V*_max_) was created by graphing the inverse of the level of substrate [S] against the reaction rate [V]. The Vmax diminished, although Km remained mostly unchanged, signifying noncompetitive inhibition of α‐amylase by the antagonists CIrGO and acarbose, as detailed in Table 2 [59].

Figure 14Michaelis–Menten (a) and Lineweaver–Burk plot (b) kinetic investigation of α‐amylase inhibition with and without an inhibitor.

**Figure:**
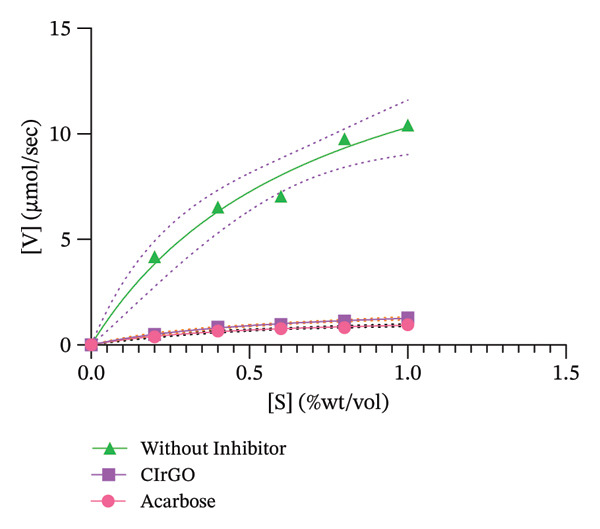
(a)

**Figure:**
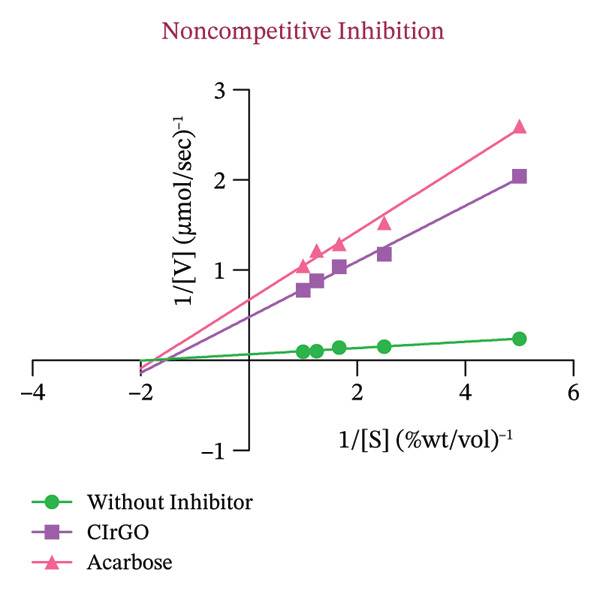
(b)

**Table 2:** Kinetic constants, *K*_*m*_ and *V*_max_, of the α‐amylase enzyme in the presence and absence of an inhibitor.

Inhibitors	IC_50_ (μg·mL^−1^)	Km (mg mL^−1^)	*V*_max_ (μmol sec^−1^)	Inhibition mode
Without inhibitor	—	0.0023	14.9476	Noncompetitive
CIrGO	84.46	0.1478	2.08724	
Acarbose	65.46	0.2541	1.49276	

#### 3.4.2. α‐Glucosidase Inhibition Assay

In comparison with the standard drug acarbose (92.00% inhibition) and Aq‐CI extract (86.09% inhibition), CIrGO functions as a complete inhibitor, showing a maximal inhibition of approximately 89.55% at the highest concentration (200 μg·mL^−1^) (Figure 15). At the prescribed dosages of 200, 100, 50, 25, and 12.5 μg·mL^−1^, CIrGO showed inhibition rates of 89.55 ± 0.03%, 52.43 ± 0.38%, 31.73 ± 0.57%, 20.23 ± 0.67%, and 11.87 ± 0.7%, respectively. At the given doses, acarbose exhibited inhibitory activities of 92.00 ± 0.01%, 64.97 ± 0.26%, 45.53 ± 0.44%, 32.77 ± 0.56%, and 18.66 ± 0.69%, respectively.

Figure 15Results of the α‐glucosidase inhibition study displayed with counters reflecting findings from three distinct investigations. The facts are presented as mean ± SEM. Values are significantly different (^∗∗∗^ *p* < 0.0001) in contrast to the control at equivalent concentrations.

**Figure:**
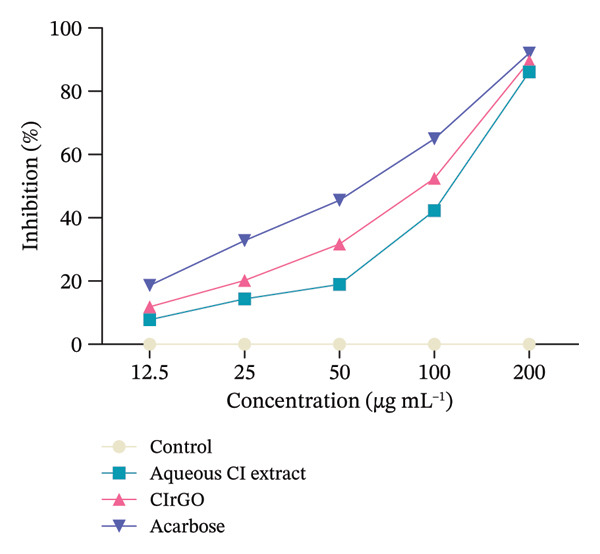
(a)

**Figure:**
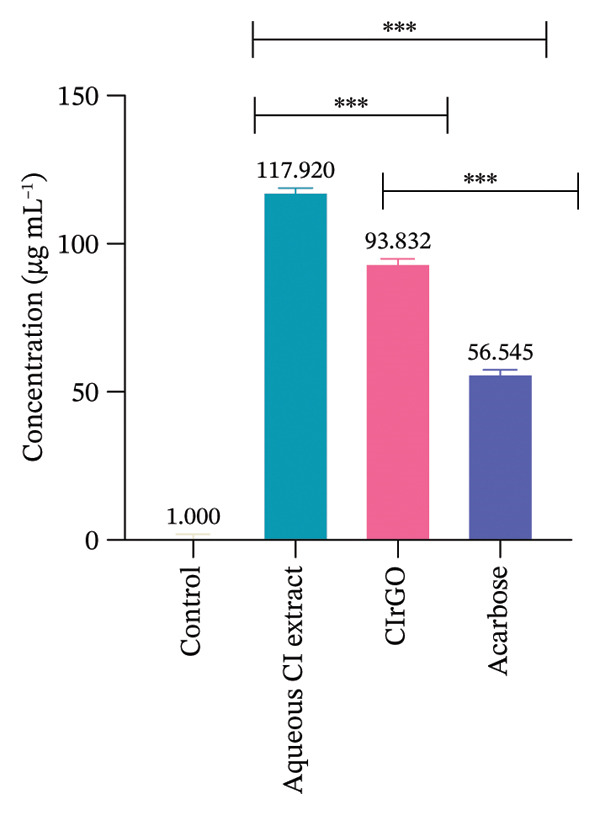
(b)

##### 3.4.2.1. Enzyme Inhibition Kinetics

The Lineweaver–Burk plot revealed the CIrGO’s kinetic inhibitory mechanism. Figure 16 displays the plot that was produced using the Lineweaver–Burk equation. According to the results, there was a singularity in the second quadrant where two reciprocal graphs with linear trajectories met. The graphs clearly illustrated the pattern of inhibition that is not competitive [60]. The α‐glucosidase enzyme was inhibited by CIrGO and acarbose, which allowed the Km and Vmax values to be determined via the Lineweaver–Burk plot (Table 3). Vmax was decreased, and Km was mostly unaltered due to the noncompetitive inhibition of α‐glucosidase by the inhibitors CIrGO and acarbose.

Figure 16Michaelis–Menten (a) and Lineweaver–Burk plot (b) kinetic analysis of the mode of inhibition of α‐glucosidase with and without an inhibitor.

**Figure:**
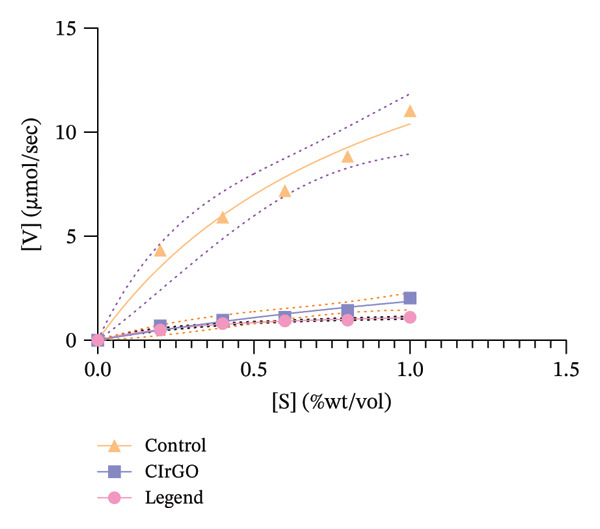
(a)

**Figure:**
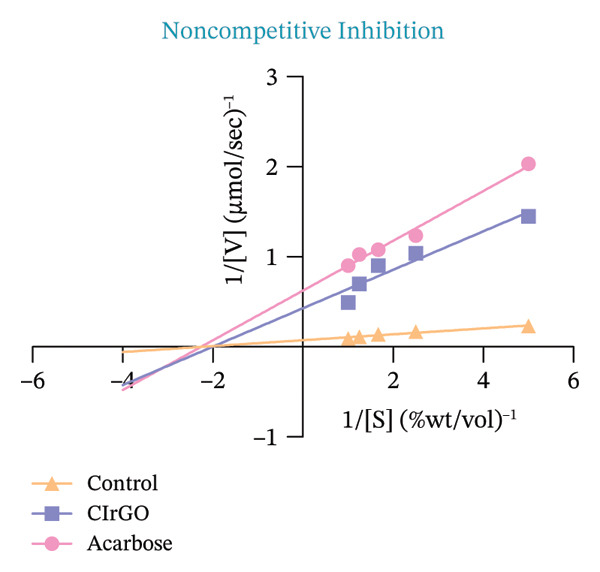
(b)

**Table 3:** Kinetic constants, Km and *V*_max_, of the α‐glucosidase enzyme in the presence and absence of an inhibitor.

Inhibitors	IC_50_ (μg·mL^−1^)	Km (mg mL^−1^)	*V*_max_ (μmol sec^−1^)	Inhibition mode
Without inhibitor	—	0.0024	13.581	Noncompetitive
CIrGO	93.83	0.0091	2.3342	
Acarbose	56.54	0.1723	1.6007	

### 3.5. Plausible Mechanism of Enzyme Inhibition and Glucose Uptake Activity

Nanoparticles have not been extensively studied in relation to α‐amylase and α‐glucosidase inhibition, but what little is known suggests that

i. these particles can bind to the enzymes and form conjugates with the amine or carboxyl groups at their N‐ or C‐termini, respectively, and
ii. this binding process can alter the native protein structure.

The CIrGO binding may have altered the structure of these enzymes, as shown by the noncompetitive inhibitory technique of CIrGO in this study. Phytoconstituents, particularly polyphenols, found plentiful in *C. igneus* leaves. The antioxidant activity of the phytoconstituents enhanced due to their superior hydrogen‐donating capacity. Furthermore, it has been demonstrated that phytoconstituents derived from several plants can block amylolytic enzymes [57, 61]. Therefore, it is highly probable that the organic phytoconstituent components attached to the CIrGO surface significantly contribute to the enhanced effectiveness of the reduced carbon component, resulting in a synergistic conferring of biological function. When it comes to antidiabetic medication therapy strategies in vivo, CIrGO might be a strong competitor. The dynamics of different cells during glucose uptake and amylolytic enzyme inhibition in the presence and absence of a CIrGO nanosheet is depicted in Figure 17.

Figure 17The process of amylolytic enzymes inhibition, and the glucose uptake activity in hepatocytes (Chang liver) and glioma cells (U87MG), shown in the illustration (a) in the absence of CIrGO nanosheets (b) in the presence of CIrGO nanosheets: CIrGO modulates intestinal carbohydrate digestion and glucose transporters and enhances glucose uptake in the liver and brain, contributing to improved glucose homeostasis.

**Figure:**
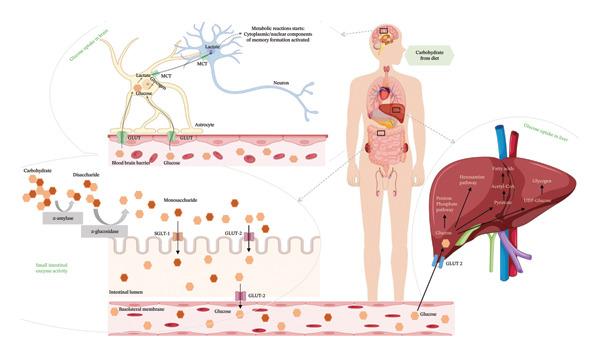
(a)

**Figure:**
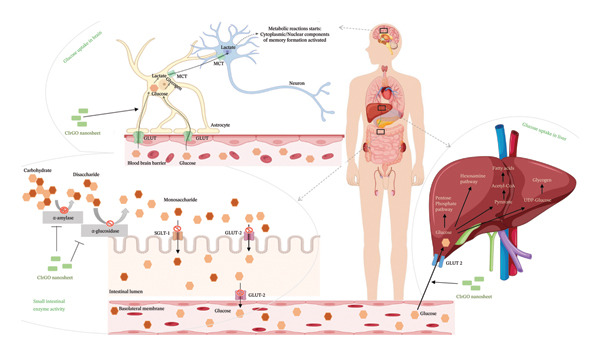
(b)

### 3.6. Computational Analysis of Amylolytic Enzymes

#### 3.6.1. Identification of the Binding Site and Ligand Parameterization

The selected protein structures for assessing relative affinity were α‐amylase (PDB‐ID: 1DHK) and α‐glucosidase (PDB‐ID: 4J5T). The acarbose molecule (41774) was selected as the reference ligand for comparison because of its established efficacy in inhibiting glycoside hydrolases (103). The selected ligands were quercetin (5280343), oleic acid (445639), diosgenin (99474), corosolic acid (6918774), cianidanol (9064), and β‐sitosterol (222284). Prior to conducting molecular docking, α‐amylase (PDB‐ID: 1DHK) and α‐glucosidase (PDB‐ID: 4J5T) structures were constructed and confirmed.

Active site residues of 1DHK (Figure 18(a)), depicted in turquoise, shown as HIS101, GLU149, TYR151, ASP197, LYS200, HIS201, GLU233, and GLU240, underlined in red (stick) and named [62]. The residues Trp391, Asp392, Arg428, Gly566, Asp568, Trp710, and Glu771 were identified in the catalytic region as the ligand‐binding site in 4J5T (Figure 18(b)). The single‐ring ligands that bind to Site B establish polar interactions with the Cwht1p residues Glu361, Glu443, Arg428, Glu429, Phe444, Val446, Gln447, and Asn448 in 4J5T (turquoise) emphasized in red (stick) and annotated [63].

Figure 18Active site residues (a) α‐amylase (PDB‐ID: 1DHK) and (b) α‐glucosidase (PDB‐ID: 4J5T).

**Figure:**
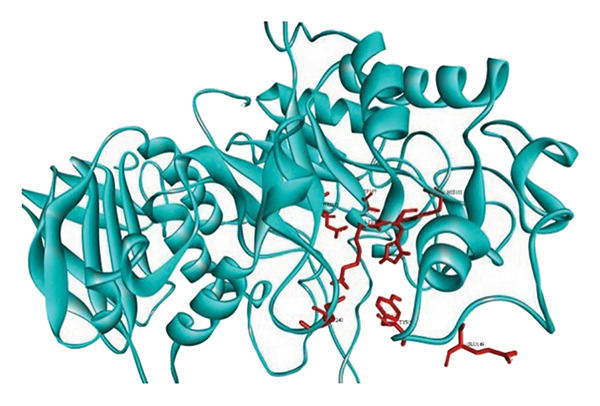
(a)

**Figure:**
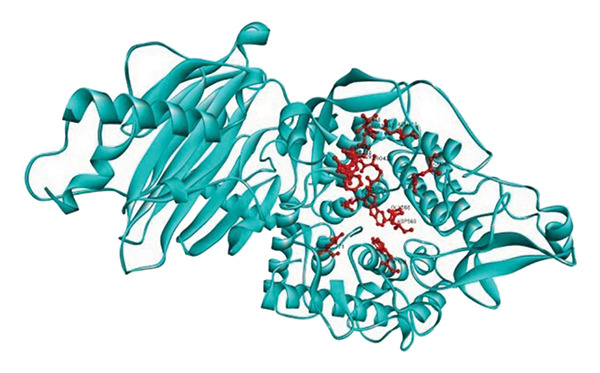
(b)

#### 3.6.2. Absorption, Distribution, Metabolism, Excretion, and Toxicity (ADMET) Analysis

An optimum pharmacokinetic, pharmacologically active, and physicochemically stable chemical molecule is required for use as a drug candidate in pharmaceutical research. New drug development studies have reduced time, money, and effort by using computer‐assisted calculations of the ADME features of therapeutic candidates. Based on the data shown in Table 4, the physicochemical features, pharmacokinetic parameters, and drug‐like qualities of substances were determined in this investigation using the Swiss ADME online web tool.

**Table 4:** ADME/T profile of major phytoconstituents from the *Costus igneus* extract, summarizing absorption, distribution, metabolism, excretion, and toxicity parameters.

Compound	Quercetin	Oleic acid	Diosgenin	Corosolic acid	Cianidanol	β‐Sitosterol	Reference: acarbose
Molecular weight (g mol^−1^)	302.24	282.46	414.62	472.70	290.27	414.71	645.60
Rotatable bonds	1	15	0	1	1	6	9
H‐bond acceptors	7	2	3	4	6	1	19
H‐bond donors	5	1	1	3	5	1	14
TPSA (Å)	131.36	37.30	38.69	77.76	110.38	20.23	321.17
Lipinski violations	0	1	1	1	0	1	3
iLOGP	1.63	4.01	4.49	3.35	1.33	5.05	1.43
CYP1A2 inhibitor	Yes	Yes	No	Yes	Yes	Yes	No
CYP2C9 inhibitor	No	Yes	Yes	Yes	No	Yes	Yes
CYP2D6 inhibitor	Yes	No	Yes	No	Yes	Yes	No
CYP2C19 inhibitor	No	No	Yes	Yes	No	No	Yes
CYP3A4 inhibitor	Yes	No	No	Yes	No	No	Yes
Synthetic accessibility	3.23	3.07	6.94	6.34	3.50	6.30	7.34

**Figure 19:**
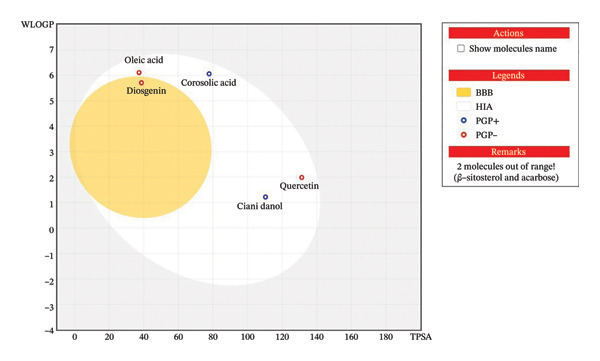
BOILED‐Egg plot showing BBB permeation (yellow), gastrointestinal absorption (white), and P‐gp behavior of key phytochemicals; two molecules fall outside the optimal range.

All the derivatives adhered to Lipinski’s rule of 5, as shown by hydrogen bond donors, hydrogen bond acceptors, number of rotatable bonds, and the value of log Po/w (iLOGP, lipophilicity). Because of its poor absorption in the intestines, the reference molecule acarbose is an outlier. The correlation between a molecule’s hydrogen bonding and its topological polar surface area (TPSA) makes it a good bioavailability predictor. Except for quercetin and acarbose, all the compounds displayed a TPSA that fell within the ideal range of 99–117.4 Å. As a result, these compounds displayed drug‐like behavior when taking ADME‐anticipated factors into account. Drug metabolism relies on cytochrome P450 (CYP) enzymes, making its inhibition an important target in research, development, and patient care. Several enzymes, including CYP1A2, CYP2C9, CYP2D6, CYP2C19, and CYP3A4, were inhibited by all the drugs [64]. A predictive model based on the lipophilicity (log P) and polarity (TPSA) of substances has been developed and used to assess the possibility of GI absorption and BBB permeability. This model is known as the BOILED‐Egg plot, which has three distinct parts to the design. According to Figure 18, molecules with a high GI absorption capacity are shown by the white area (albumin) of the cooked egg plot, whereas molecules with a probable permeability across the BBB are shown by the yellow area. For compounds with little brain penetration and poor gastrointestinal absorption, the gray area is reserved. When given orally, quercetin, oleic acid, corosolic acid, and cianidanol are more likely to show bioavailability since they are placed in the white zone, which indicates a considerable potential for gastrointestinal absorption. Indicating BBB permeability was diosgenin, which was in the yellow zone. The specified range of these levels was exceeded by two compounds, β‐sitosterol and acarbose. Predicting glycoprotein permeability (PGP) is another function of the BOILED‐Egg plot. The red dots on the graph (Figure 19) show chemicals that are not expected to be pumped out of cells by P‐glycoprotein (Pgp). This means that these compounds may have higher absorption qualities [65]. The bioavailability score of each chemical was 0.55. Therefore, the in silico data indicated that these compounds could be a good choice for creating new orally active medications, and it also confirmed that all the compounds had the ability to be bioavailable when taken orally.

The ADMET data (Table 4) provide a comprehensive assessment, acting as a crucial resource for directing future development and research of the evaluated drugs for possible therapeutic applications. In addition to the individual evaluations of each chemical, the comprehensive data in the ADMET table provide new perspectives on the toxicological and pharmacokinetic profiles of the synthesized metal compounds. It is possible to see trends and patterns in the collective characteristics of all molecules that would not be obvious when examining each compound alone. Recognizing similarities and differences among the chemicals is a crucial part. For instance, certain compounds may have similar solubility or blood–brain barrier penetration, whereas others may have distinctive features. Strategies for improving medication development or targeting specific biological pathways might be formulated by understanding these common qualities and distinctions. Furthermore, the combined results provide insight into possible adverse interactions or synergistic effects that could occur when multiple medications are taken simultaneously. By meticulously examining the CYP2D6 breakdown, toxicity to the liver, and adherence to plasma proteins of all pharmaceuticals, investigators can improve the safety as well as efficacy of combination therapy. This methodology will facilitate the prediction and prevention of probable drug–drug interactions. The extensive findings underscore the necessity of considering several elements in pharmaceutical research and development. Drug acceptability and safety are affected by characteristics such as metabolism and hepatotoxicity, whereas bioavailability and CNS targeting depend on features including blood–brain barrier permeability and solubility [66]. By consolidating diverse criteria into a cohesive analysis, researchers can attain a more profound comprehension of the pharmacological attributes and possible hazards of substances, facilitating more enhanced decision‐making in drug development. The findings offer an extensive summary of the toxicological and pharmacokinetic characteristics of these nanosheets, representing a significant progress in enhancing their therapeutic effectiveness and minimizing potential risks in healthcare settings. This aspect represents the novel and significant contribution of the findings.

#### 3.6.3. Induced‐Fit Docking

To find out how ligands attached to α‐amylase (PDB: 1DHK) and α‐glucosidase (PDB: 4J5T) conform, molecular docking was used. To ascertain the optimal complex, various metrics were employed, including the value of root‐mean‐square deviation (RMSD), the least binding affinity, and the quantity of interfacing residues. To determine the distance between the atoms of the complex’s constituents, the RMSD value was utilized. The lowest RMSD would indicate the most stable binding conformation. One biomolecule’s binding affinity to its ligand or binding partner is the strength of the interaction between the two biomolecules. For an association to be stable and strong, the binding affinity should have a small negative value [67]. When it comes to finding the optimal position, residues involved in binding were crucial. One method for screening potential ligands is by evaluating their ability to bind to the residues of the active site. The data supporting the docking results are provided in the Supporting file.

##### 3.6.3.1. Elucidating Molecular Docking Interactions of Compounds With α‐Amylase

Quercetin (5280343): Docking of 1DHK was conducted with quercetin utilizing AutoDockTools. The docking contacts occur at residues HIS101, LYS200, ASP197, and LYS200. The interactions transpire at the active site or within the pocket housing the active site residue. Four hydrogen bonds were found, indicating stable interactions, with −7.35 kcal/mol binding energy (Supporting Table 1, Figure S1).

Oleic acid (445639): Interactions occur at residues LYS200 and ILE235. Two hydrogen bonds were found, contributing to stable interactions, with −5.03 kcal/mol binding energy (Supporting Table 2, Figure S2).

Diosgenin (99474): Interactions occur at residues GLU233 and ASN298. One of the interactions (GLU233) occurs at the active site or within the pocket housing the active site residue. The residue, ASN298, is located inside the pocket’s range. Two hydrogen bonds were detected, indicating stable interactions, despite a binding energy of −9.08 kcal/mol (Supporting Table 3, Figure S3).

Corosolic acid (6918774): The docking findings indicated that the contact occurs at residue VAL163. The interaction is not taking place at the active site or within the pocket housing the active site residue. Upon inspection, we discovered that VAL163 is in close proximity to the active site residues. Consequently, a single hydrogen bond demonstrates an inferior binding energy of −9.42 kcal/mol relative to others (Supporting Table 4, Figure S4).

Cianidanol (9064): Interactions occur at residues ASP 300, TYR 151, LYS 200, and GLU 233. Four interactions are taking place at the active site or within the pocket housing the active site residue. Upon inspection, we discovered that interactions with TYR151, LYS200, and GLU233 are located in the pocket, while one interaction with ASP300 is near the active site residue, exhibiting favorable binding energy. Consequently, the presence of hydrogen bonds and a favorable binding energy of −7.08 kcal/mol suggests that cianidanol may exhibit activity (Supporting Table 5, Figure S5).

β‐Sitosterol (222284): The interaction occurs at residue LYS200. One hydrogen bond is detected, contributing to a stable contact, despite a binding energy of −9.03 kcal/mol (Supporting Table 6, Figure S6).

Acarbose (41774): Interactions occur at residues GLU233, TY151, GLU240, and ILE235. The interactions transpire at the active site or within the pocket housing the active site residue. Five hydrogen bonds were discovered, indicating stable interactions, despite a binding energy of −4.49 kcal/mol. Therefore, it may be stated that “reference” may demonstrate the anticipated activity with the protein target 1DHK, albeit the stability of contacts requires verification (Supporting Table 7, Figure S7).

##### 3.6.3.2. Elucidating Molecular Docking Interactions of Compounds With α‐Glucosidase

Quercetin (5280343): The docking of 4J5T with quercetin was conducted with AutoDockTools. The interactions occur at residues GLN447, ILE362, LEU364, PHE444, and ARG428. Three out of five interactions occur at the active site or within the pocket housing the active site residue. Five hydrogen bonds were found, indicating stable interactions, with −8.58 kcal/mol binding energy (Supporting Table 8, Figure S8).

Oleic acid (445639): Interactions occur at residues TRP425 and ASN448. One of the interactions occurs at the active site or within the pocket housing the active site residue. Two hydrogen bonds were found, indicating stable interactions, with a low binding energy of −6.27 kcal/mol (Supporting Table 9, Figure S9).

Diosgenin (99474): Interaction at residue ILE362. The contact is taking place outside the active site or pocket. The residue ILE362 is next to the active site pocket. Despite the binding energy being −8.41 kcal/mol, the interaction occurs outside the active site pocket. Consequently, we can conclude that diosgenin may demonstrate activity, albeit with modest efficacy, thus rendering it an inadequate choice against this target (Supporting Table 10, Figure S10).

Corosolic acid (6918774): The interaction occurs at residue ASP568, either in the active site or within the pocket housing the active site residue. The system achieved a stable binding energy minimum of −9.7 kcal/mol (Supporting Table 11, Figure S11).

Cianidanol (9064): Interactions occur at residues GLU361, GLN447, ASN448, and ILE362. Four interactions are taking place at the active site or within the pocket housing the active site residue. Upon inspection, we discovered that one interaction with ILE362 is in close proximity to the active site residue, exhibiting favorable binding energy. Consequently, the presence of hydrogen bonds and a favorable binding energy of −8.71 kcal/mol suggest that cianidanol may exhibit activity (Supporting Table 12, Figure S12).

β‐Sitosterol (222284): The interaction occurs at residue GLU771. The interaction takes place at the active site or within the pocket housing the active site residue. One hydrogen bond is detected, contributing to a stable contact, with −8.93 kcal/mol binding energy (Supporting Table 13, Figure S13).

Acarbose (41774): Interactions occur at residues GLU771, ASN448, GLU429, ILE362, GLU361, GLN447, ARG428, and GLU429. Eight out of nine interactions occur at the active site or within the pocket housing the active site residue. Nine hydrogen bonds were found, indicating stable interactions, with −7.43 kcal/mol binding energy (Supporting Table 14, Figure S14).

Quercetin, oleic acid, diosgenin, corosolic acid, cianidanol, and β‐sitosterol derivatives were docked against α‐amylase and α‐glucosidase. Corosolic acid was selected for exhibiting inferior binding energy, i.e., −9.42 kcal/mol when compared to acarbose (−4.49 kcal/mol) for α‐amylase. In opposition to α‐glucosidase once more, corosolic acid has the lowest binding affinity, i.e., −9.72 kcal/mol as compared to the acarbose control (−7.43 kcal/mol). Corosolic acid interacts with α‐amylase at site Val163 (Figure 20(a)), while the control, acarbose, interacts with GLU233, TYR151, ILE235, and GLU240 (Figure 20(c)). α‐Glucosidase is associated with the ASP568 residue (Figure 20(b)), while acarbose is linked to the GLU771, ASN448, GLU429, ILE362, GLN447, and ARG428 residues (Figure 20(d)). The ideal binding conformation with α‐glucosidase and α‐amylase with 2D and 3D interactions of ligand–receptor, as compared to acarbose, is mentioned in the Supporting file. For the simulation investigation, corosolic acid was selected due to its low binding energy and good docking position in comparison with reference acarbose.

Figure 20Using AutoDockTools, complex showing interaction between 1DHK with (a) corosolic acid and (c) acarbose; complex showing interaction between 4J5T with (b) corosolic acid and (d) acarbose.

**Figure:**
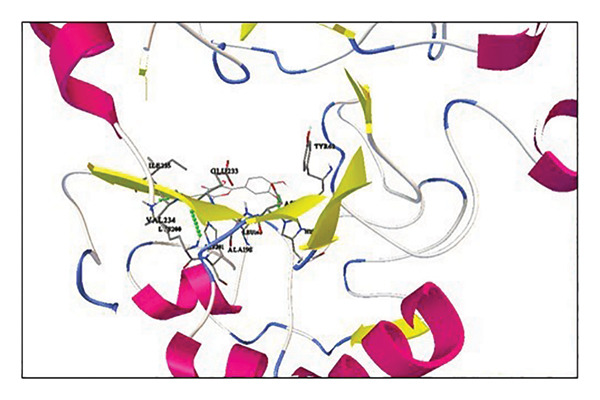
(a)

**Figure:**
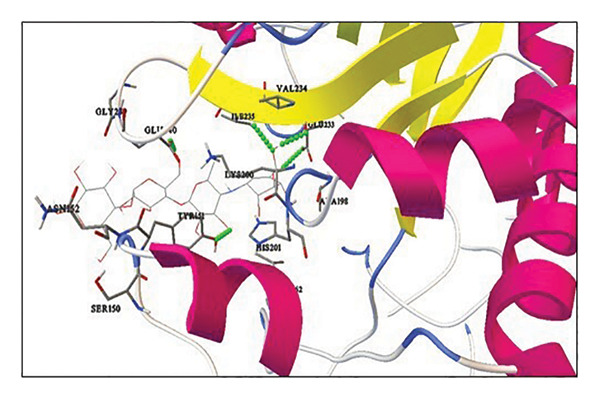
(b)

**Figure:**
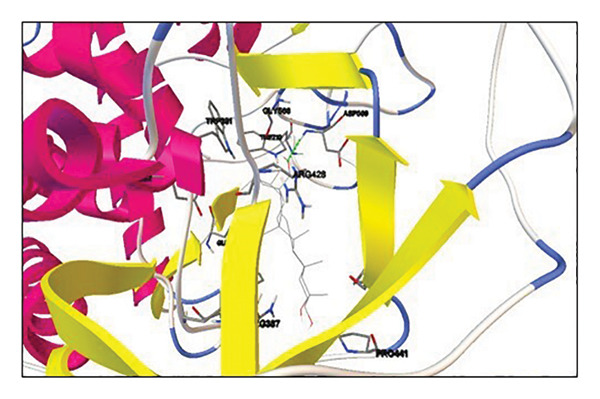
(c)

**Figure:**
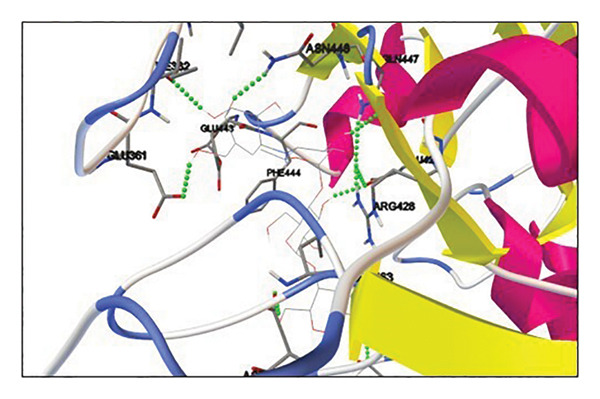
(d)

#### 3.6.4. Simulation Modeling

##### 3.6.4.1. Delving Into the Simulated Dynamic Interplay of 1DHK and Complex Corosolic Acid

Using GROMACS, the COMPLEX corosolic acid‐1DHK was simulated. When it comes to MD simulation, GROMACS is lightning fast. It can work with force fields from GROMOS, OPLS, AMBER, and ENCAD, but it does not have one built‐in. The tool is quite adaptable, and it is easy to personalize the analysis. Roughly one hundred analysis and utility programs are part of the bundle. According to the General Public License, GROMACS is free and open to anybody to use. The dynamics studies utilized CHARMM36 force field. Potential energy (Epot) is one of the key metrics to evaluate the efficacy of energy minimization (109). System size and the amount of water molecules determine the minimum value of Epot, which should be negative and in the order of 10^5^. For COMPLEX corosolic acid‐1DHK, the Epot was measured to be −1.03737 × 10^−6^ kJ/mol. The energy‐minimizing and potential‐energy‐stable COMPLEX corosolic acid‐1DHK is shown in the plot (Figure 21(a)). A solid foundation for the subsequent simulations is guaranteed by energy minimization. Attaining equilibration for the system was possible after it was energy reduced and appraised positively for potential energy. Within 1 nanosecond, the system was equilibrated with respect to both pressure and temperature. Temperature equilibration was accomplished by the COMPLEX corosolic acid‐1DHK in 1000 ps or 1 nanosecond. The stabilization of the system’s temperature is achieved through temperature equilibration. The system also achieved pressure equilibration in less than one thousandth of a second. The system is well‐equilibrated after the energy minimization and temperature and pressure balancing processes. This was accomplished using GROMACS for the final MD simulation [68]. A 100‐ns simulation was conducted on the system, and its trajectory was subsequently utilized for the investigation of RMSD, RMSF, and radius of gyration.

Figure 21The graphs illustrate (a) potential energy plot, (b) root‐mean‐square deviation plot, (c) radius of gyration plot, (d) root‐mean‐square fluctuation plot of complex corosolic acid‐1DHK.

**Figure:**
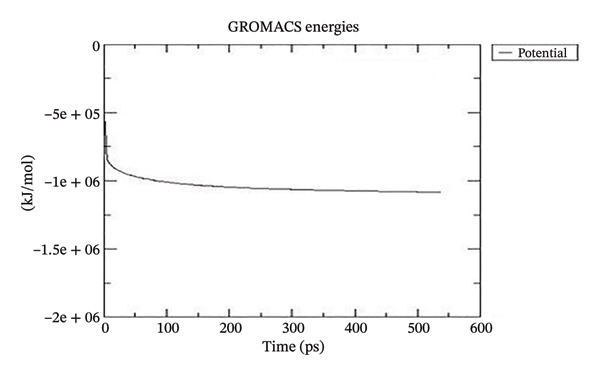
(a)

**Figure:**
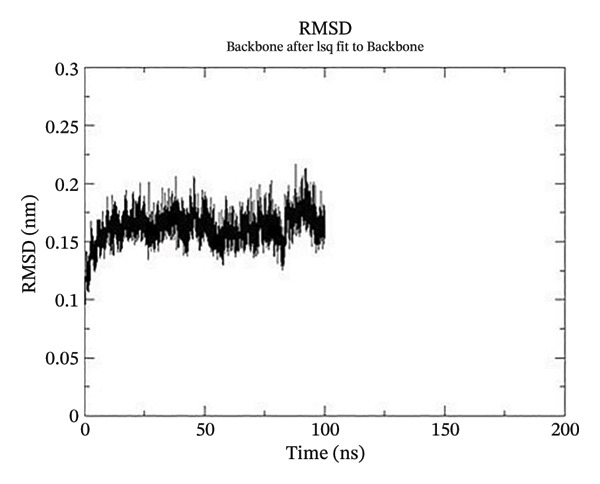
(b)

**Figure:**
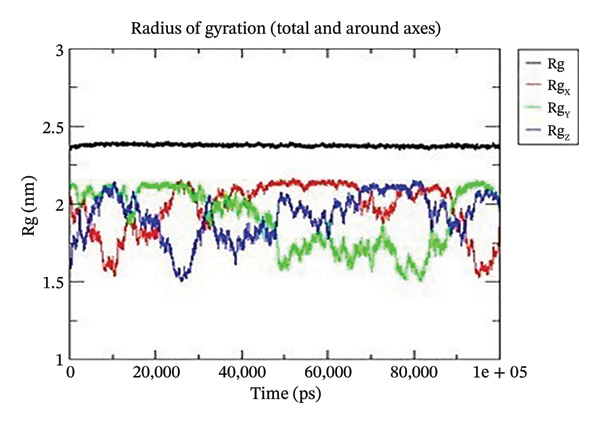
(c)

**Figure:**
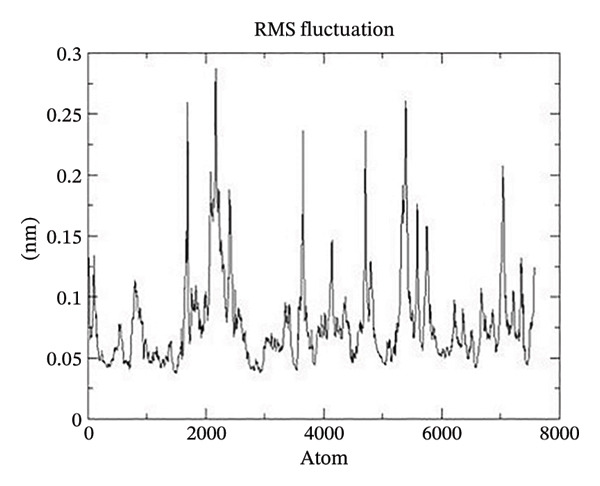
(d)

A built‐in utility called rms is included in GROMACS for calculating RMSD. Based on the data, it was concluded that the COMPLEX corosolic acid‐1DHK protein exhibits an RMSD of less than 0.2 nm for the whole 100‐ns period (Figure 21(b)). The protein (in complex) is exhibiting very little structural variation, suggesting that it is quite stable, as RMSD displays the protein’s variation when all factors are included. Protein compactness can be assessed using GROMACS’s built‐in feature for calculating the radius of gyration. A robust protein conformation guarantees that its radius of gyration remains comparatively steady. Protein gyration radius in COMPLEX corosolic acid‐1DHK is quite constant, according to the graph (Figure 21(c)). Colors red, blue, and green represent the radii of rotation in the *x*, *y*, and *z* dimensions, respectively. These values are used to construct 3D structures, as illustrated in black. Throughout the experiment, the protein managed to keep its compactness, as shown by the stable graph (black color).

Analysis of the docked complex using RMS fluctuation (RMSF) demonstrated its stability. No significant change to the protein’s structure is indicated by the observed range of RMSF, which is less than 0.3 nm (Figure 21(d)). The stability of the complex is indicated by the aforementioned investigation.

##### 3.6.4.2. Delving Into the Simulated Dynamic Interplay of 4J5T and Complex Corosolic Acid

For COMPLEX corosolic acid‐4J5T, the calculated Epot was −1.92737 × 10‐6 kJ/mol. The energy‐minimizing and potential‐energy‐stable COMPLEX corosolic acid‐4J5T is shown in the plot (Figure 22(a)).

Figure 22The graphs illustrate (a) potential energy plot, (b) root‐mean‐square deviation plot, (c) radius of gyration plot, (d) root‐mean‐square fluctuation plot of COMPLEX corosolic acid‐4J5T.

**Figure:**
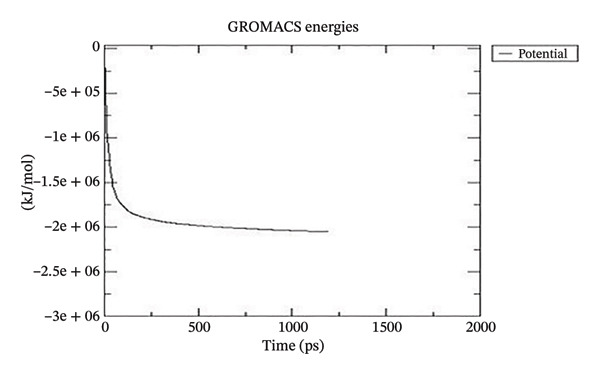
(a)

**Figure:**
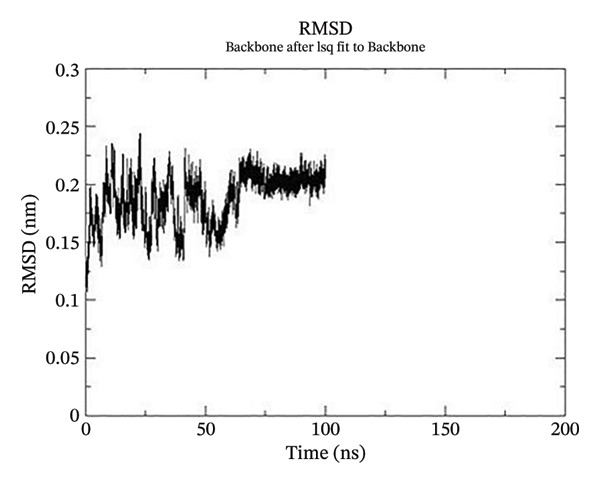
(b)

**Figure:**
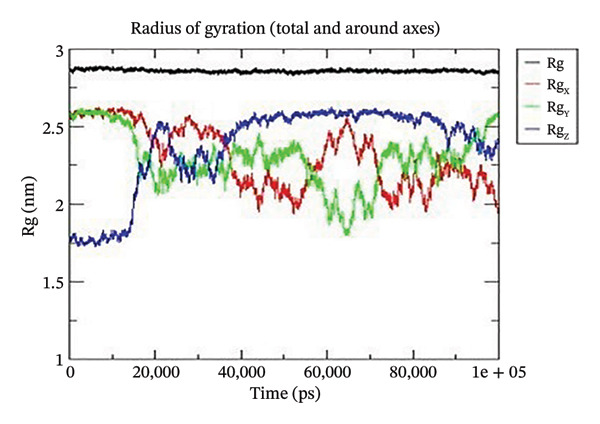
(c)

**Figure:**
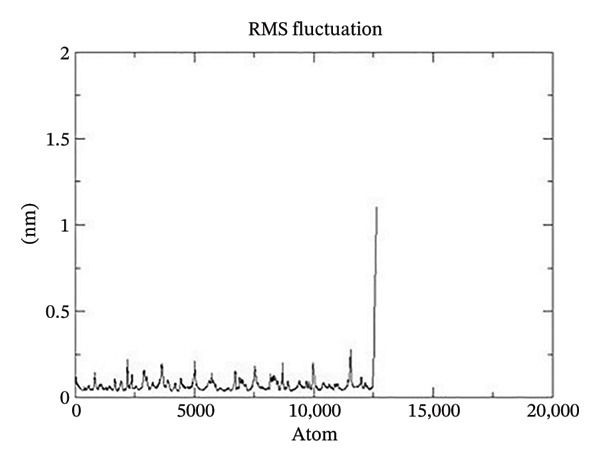
(d)

Analysis of Figure 22(b) indicates that over a period of 100 ns, the protein in the COMPLEX corosolic acid‐4J5T demonstrates a minimal RMSD, fluctuating between 0.20 and 0.25 nm. This RMSD reflects the protein’s variation, implying that the protein (in complex) undergoes negligible structural alteration, thereby indicating its remarkable stability.

The protein in COMPLEX corosolic acid‐4J5T has a reasonably steady radius of gyration, according to the graph analysis (Figure 22(c)). As illustrated in black, the radii of gyration in the *x*, *y*, and *z* dimensions are represented by red, blue, and green, respectively. These dimensions are used in the 3D structure. It is demonstrated that the protein may preserve its compactness during the simulation by the stable graph, which is black in color.

The stability of the docked complex was demonstrated by the analysis of RMSF. As seen in Figure 22(d), the RMSF range is less than 0.5 nm, indicating that there has been no notable alteration to the protein structure. All the preceding analysis points to a stable complex.

## 4. Discussion

Nanotechnology has emerged as a significant technological advancement with widespread applications across various sectors, including agriculture, food production, medicine, and biomedical engineering. Renowned for their minuscule size, versatility, and compatibility with a variety of materials such as optical, textile, magnetic, electronic, mechanical, and chemical substances, nanoparticles offer promising possibilities for therapeutic applications [5, 7]. These nanoparticles exhibit diverse properties, including antibacterial, antioxidant, antidiabetic, and anticancer effects. Graphene, selected for green synthesis, is lightweight and chemically stable and exhibits excellent electron conductivity [9–15]. Graphene is synthesized through various innovative methods. Natural graphite is oxidized to graphite oxide, which is subsequently exfoliated by sonication to yield GO. The reduction process is indicated by a color change from brownish–yellow to black [69]. The study and utilization of nanoparticles are deeply rooted in the integration of physical, chemical, and biological scientific disciplines [70]. rGO has garnered significant interest due to its exceptional thermal conductivity, electrical properties, high Young’s modulus, mechanical robustness, optical transparency, and relevance in theoretical studies [15]. Owing to these properties, rGO is extensively employed across a wide spectrum of technological applications and is typically synthesized via the chemical reduction of GO. Conventional reducing agents, such as hydrazine, though effective, pose considerable environmental and health risks due to their toxic nature. Consequently, there is a growing research focus on identifying environmentally benign alternatives for the reduction of GO [71]. The leaves of *C. igneus*, a plant from the *Costaceae* family, contain various phytochemicals such as glycosides, carbohydrates, alkaloids, saponins, flavonoids, tannins, phytosterols, proteins, phenolic compounds, and amino acids that serve as effective reducing agents [16–20, 25]. *C. igneus*, also known as fiery costus or the “insulin plant,” is of particular interest due to its potential therapeutic uses, particularly in the treatment of diabetes [26]. The successful synthesis of graphene nanosheet from this plant has been confirmed through various analytical techniques, including XRD, Raman spectroscopy, UV–vis, and FTIR spectroscopy. The surface of GO contains various oxygen‐rich functional groups, including epoxide, hydroxyl, and carbonyl. Under basic conditions, epoxide and carbonyl groups can be converted into hydroxyl groups. *C. igneus* leaf extract is abundant in polyphenols, which possess highly acidic hydrogen atoms that readily ionize into their anionic forms. These anionic polyphenols engage in nucleophilic substitution with GO’s hydroxyl groups, leading to the formation of an intermediate. The subsequent removal of a water molecule facilitates the transformation of this intermediate into rGO [47–49]. The biosynthesis of nanoparticles, particularly from plant extracts, has attracted significant attention in biomedical research due to their biocompatibility and broad range of applications. Many conventional oral antidiabetic medications struggle to maintain stable blood glucose control. In contrast, extracts from medicinal plants have demonstrated efficacy in reducing blood glucose levels, making them valuable alternatives for managing diabetes. This study investigates the antidiabetic potential of the *C. igneus* leaf extract and its rGO nanosheet using in vitro, kinetic, and in silico models. The glucose oxidase‐peroxidase method was employed to measure glucose absorption in Chang liver and U87MG cells. The results demonstrated a significant increase in glucose uptake in both cell types treated with the plant extract and nanosheets compared to the positive control, metformin. At higher concentrations, nanosheets enhanced glucose absorption in both cell lines; i.e., the uptake activity of Chang liver cells was 81.39% when treated with CIrGO, which was significantly higher than U87MG cells’ 78.54%, and significantly different from the control (*p* < 0.0001). These findings suggest that the type of cell used in glucose absorption assays influences the results. Additionally, the rate of glucose metabolism and the internal glucose concentration within cells play critical roles in determining glucose uptake. The enhanced glucose uptake in hepatocytes and glioma cells may be attributed to facilitated diffusion and enhanced glucose metabolism [54]. The hypoglycemic effects of polyphenols are primarily attributed to their ability to inhibit carbohydrate absorption in the intestine, modulate glucose absorption by modifying enzyme activity, enhance β‐cell function and insulin action, promote insulin secretion, and provide antioxidant and anti‐inflammatory benefits. These findings were consistent with studies investigating the metabolic effects of CIrGO derivatives. Angiopep‐2‐modified doxorubicin‐loaded GO (ANG‐Dox‐GO) in U87MG cells, showing enhanced cellular uptake and antiproliferative effects. Although glucose uptake was not directly measured, the increased metabolic activity suggests that such nanomaterials could influence glucose metabolism. Similarly, another study involving hyaluronic acid–modified rGO for targeted therapy in U87 glioblastoma cells demonstrated that rGO nanocomposites facilitated enhanced intracellular uptake, which could imply alterations in glucose utilization. Although direct studies on glucose uptake in Chang liver cells treated with rGO are limited, there are relevant studies on HepG2 liver cells that provide insights into the potential effects of rGO on cellular metabolism [72]. Fe_3_O_4_‐rGO nanocomposites demonstrate cytotoxicity in HepG2 cells by triggering oxidative stress and apoptosis, which may subsequently disrupt glucose metabolism through indirect pathways. These findings suggest that rGO derivatives could play a role in altering glucose metabolism, even though direct evidence from glucose uptake assays in liver cells is scarce [73]. The digestion of oligosaccharides and disaccharides into monosaccharides by enzymes like pancreatic α‐amylase and intestinal α‐glucosidase leads to an increase in blood glucose levels. Inhibiting these enzymes can, therefore, reduce blood glucose levels in diabetic patients [57]. The study found that both the aqueous *C. igneus* extract and its nanosheets inhibited α‐amylase and α‐glucosidase in a concentration‐dependent manner, with significant inhibition compared to the positive control, acarbose. Although acarbose is effective in controlling blood glucose levels, it can cause side effects, such as abdominal discomfort and flatulence. This has led to the exploration of natural alternatives with fewer side effects. CIrGO demonstrated a dose‐dependent inhibitory effect against both enzymes, with an IC_50_ of 84.46 μg·mL^−1^, compared to acarbose’s IC_50_ of 65.46 μg·mL^−1^. The aqueous *C. igneus* extract exhibited a slightly higher IC_50_ of 92.62 μg·mL^−1^. At 50 μg·mL^−1^, CIrGO inhibited α‐glucosidase by 89%, while acarbose showed 92.05% inhibition. Kinetic analysis revealed a noncompetitive inhibition pattern for both α‐amylase and α‐glucosidase, with both CIrGO and acarbose causing a decrease in Vmax while maintaining nearly the same *K*_*m*_. Despite the limited number of studies examining the impact of biologically synthesized rGO on α‐amylase and α‐glucosidase inhibition, this research demonstrates that *C. igneus* leaf extract and its rGO nanosheet effectively inhibit this enzyme. Further studies have explored the inhibitory effects of rGO‐based nanocomposites on α‐glucosidase activity. A study involving palladium‐decorated rGO (Pd‐rGO) demonstrated significant α‐glucosidase inhibition, highlighting the potential of rGO derivatives in managing postprandial hyperglycemia [74]. Additionally, research on rGO has revealed significant changes in membrane potential and gene expression following treatment. This suggests that rGO may alter cellular functions related to ion transport and metabolic processes, which could indirectly influence the activity of amylolytic enzymes. However, direct evidence of rGO’s effect on α‐amylase and α‐glucosidase remains limited [75]. Cytotoxicity assays confirmed that both the Aq‐CI extract and CIrGO exhibited concentration‐dependent toxicity, with higher concentrations increasing cell toxicity. In Chang liver cells, IC_50_ for CIrGO was 235.72 μg·mL^−1^, and for Aq‐CI, it was 303.89 μg·mL^−1^, compared to metformin’s IC_50_ of 327.01 μg·mL^−1^. In U87MG cells, IC_50_ for CIrGO was 199.73 μg·mL^−1^, and for the Aq‐CI extract, it was 276.34 μg·mL^−1^, compared to metformin’s IC_50_ of 346.74 μg·mL^−1^. These results align with previous studies [21, 53] that report dose‐dependent cytotoxic effects of plant extracts on cells. Furthermore, studies have shown that rGO can reduce cancer cell viability by inducing oxidative stress, apoptosis, and damage to cell membranes. The higher cytotoxicity observed in U87MG cells suggests that CIrGO selectively induces apoptosis in cancer cells more effectively than in normal cells. This selectivity may stem from the enhanced generation of ROS in malignant cells, leading to oxidative stress, disruption of cellular membranes, and programmed cell death. These findings align with earlier reports indicating that elevated concentrations of rGO induce ROS‐mediated cytotoxicity and apoptosis in cancer models [53]. Furthermore, the presence of phytochemicals adsorbed onto CIrGO surfaces may enhance its bioactivity and cellular uptake. This synergistic combination of CIrGO’s nanostructure and the flavonoid‐rich plant extract contributes to its multifunctionality [76]. Molecular docking studies identified corosolic acid as a promising antidiabetic compound within *C. igneus* leaf extract among various derivatives investigated based on literature search [25, 26, 62, 63]. ADMET profiling indicated that corosolic acid has favorable pharmacokinetic properties, with no adverse effects on absorption, and it adheres to various drug‐likeness criteria. The stability of the corosolic acid–protein complex was confirmed through MD simulations, which showed minimal structural changes, suggesting the stability of the compound. This study highlights corosolic acid as the key bioactive compound contributing to the antidiabetic effects of the *C. igneus* leaf extract.

## 5. Conclusion

The *C. igneus* was successfully utilized to synthesize CIrGO, which was subsequently analyzed using conventional analytical techniques. The synthesized CIrGO was found to be in the form of a thin nanosheet. The EDAX spectrum confirmed the elemental composition with carbon (86%) and oxygen (13%) as the predominant elements. The Aq‐CI contains a variety of phytochemicals, including glycosides, carbohydrates, alkaloids, saponins, flavonoids, tannins, phytosterols, proteins, phenolic compounds, and amino acids, which serve as effective reducing agents during the synthesis process. At higher concentrations, CIrGO exhibited toxicity, with 28.26% cell viability at 400 μg·mL^−1^. Upon incubation with different concentrations, CIrGO significantly inhibited the activities of α‐amylase and α‐glucosidase in a concentration‐dependent manner while enhancing glucose uptake in both Chang liver and U87MG cells. Human glucose regulation is primarily controlled by insulin and glucagon, and dysregulation of these hormones can lead to conditions such as diabetes and hyperglycemia. The study demonstrated that eco‐friendly synthesis of CIrGO and the phytochemicals in the *C. igneus* extract interacted with these hormones to modulate glucose metabolism. The interaction between the graphene nanosheet and the phytochemicals involved in the biosynthesis of CIrGO with the amino acid residues of carbohydrate‐hydrolyzing enzymes likely contributed to the observed reduction in enzyme activity. Further validation through molecular docking and interaction studies supported these findings, revealing how the plant‐derived phytochemicals interacted with the amino acid residues of α‐amylase and α‐glucosidase to regulate glucose metabolism. According to these insights, the produced CIrGO could be a promising nanomedicine for the management of diabetes.

**Nomenclature**
GO: Graphene oxide
rGO: Reduced graphene oxide
CIrGO: *Costus igneus*–derived reduced graphene oxide
T2DM: Type II diabetes mellitus
Aq‐CI: *Costus igneus* leaves aqueous extract
DNS: Dinitrosalicylic acid
ADMET: Absorption, distribution, metabolism, excretion, and toxicity
FTIR: Fourier‐transform infrared
XRD: X‐ray diffraction
TPSA: Topological polar surface area
CYP: Cytochrome P450
BBB: Blood–brain barrier

## Author Contributions

Mansi Yadav: writing–original draft, conceptualization, methodology, and data curation; Divya Vashishth: writing–review and editing; Monika Bhardwaj: writing–review and editing; Jaya Parkash Yadav: conceptualization and supervision; Sudhir Kumar Kataria: conceptualization and supervision.

## Funding

No funding was received for this manuscript.

## Conflicts of Interest

The authors declare no conflicts of interest.

## Supporting Information

Supporting Information Description: This supporting information provides additional information and data supporting the study presented in the main manuscript. It includes additional data and images related to molecular docking analyses of selected bioactive compounds with human pancreatic α‐amylase (PDB‐ID: 1DHK) and α‐glucosidase (PDB‐ID: 4J5T) to elucidate their docking parameters and visualization images of their interactions.

Figure S1 (visualization for the docking of 1DHK with quercetin), Figure S2 (visualization for the docking of 1DHK with oleic acid), Figure S3 (visualization for the docking of 1DHK with diosgenin), Figure S4 (visualization for the docking of 1DHK with corosolic acid), Figure S5 (visualization for the docking of 1DHK with cianidanol), Figure S6 (visualization for the docking of 1DHK with β‐sitosterol), Figure S7 (visualization for the docking of 1DHK with reference acarbose), Figure S8 (visualization for the docking of 4J5T with quercetin), Figure S9 (visualization for the docking of 4J5T with oleic acid), Figure S10 (visualization for the docking of 4J5T with diosgenin), Figure S11 (visualization for the docking of 4J5T with corosolic acid), Figure S12 (visualization for the docking of 4J5T with cianidanol), Figure S13 (visualization for the docking of4J5T with β‐sitosterol), Figure S14 (visualization for the docking of 4J5T with reference acarbose), TableS1 (docking parameters for 1DHK with quercetin), Table S2 (docking parameters for 1DHK with oleic acid), Table S3 (docking parameters for 1DHK with diosgenin), Table S4 (docking parameters for 1DHK with corosolic acid), Table S5 (docking parameters for 1DHK with cianidanol), Table S6 (docking parameters for 1DHK with β‐sitosterol), Table S7 (docking parameters for 1DHK with reference acarbose), Table S8 (docking parameters for 4J5T with quercetin), Table S9 (docking parameters for 4J5T with oleic acid), Table S10 (docking parameters for 4J5T with diosgenin), Table S11 (docking parameters for 4J5T with corosolic acid), Table S12 (docking parameters for 4J5T with cianidanol), Table S13 (docking parameters for 4J5T with β‐sitosterol), and Table S14 (docking parameters for 4J5T with reference acarbose).

## Supporting information


**Supporting Information** Additional supporting information can be found online in the Supporting Information section.

## Data Availability

The data that support the findings of this study are available in the supporting information of this article.
