# Supplementary material for: Bio‐Assisted Synthesis of Reduced Graphene Oxide Nanosheets From Graphene Oxide: Promising and Efficient Cytotoxic and Antidiabetic Potency in In Vitro, Kinetic, and In Silico Models
Source: Int J Biomater. 2026 Feb 17;2026:5521416. doi: 10.1155/ijbm/5521416 (PMC12911526; doi:10.1155/ijbm/5521416)
Supplement: Supplementary file 1 — Supporting Information Additional supporting information can be found online in the Supporting Information section. [file IJBM-2026-5521416-s001.docx]

**SUPPLEMENTARY FILE**

**3.6.3.1 Elucidating molecular docking interactions of compounds with α-amylase**

Table S1. Docking parameters for 1DHK with Quercetin

| Quercetin Efficiency | -0.33 |
| --- | --- |
| Inhibition constant | 4.12uM |
| Vdw_hb_desolv_energy | -8.62 |
| Electrostatic energy | -0.52 |
| Total internal energy | -0.94 |
| Torsional energy | 1.79 |
| Unbound energy | -0.94 |


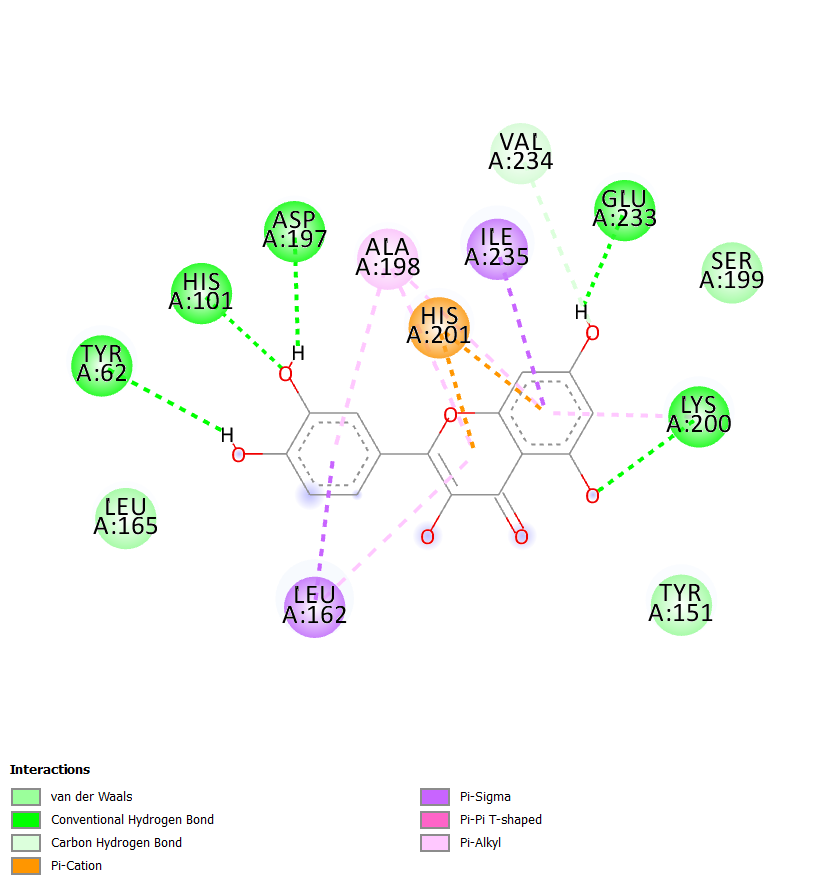


2D interaction image for 1DHK with Quercetin


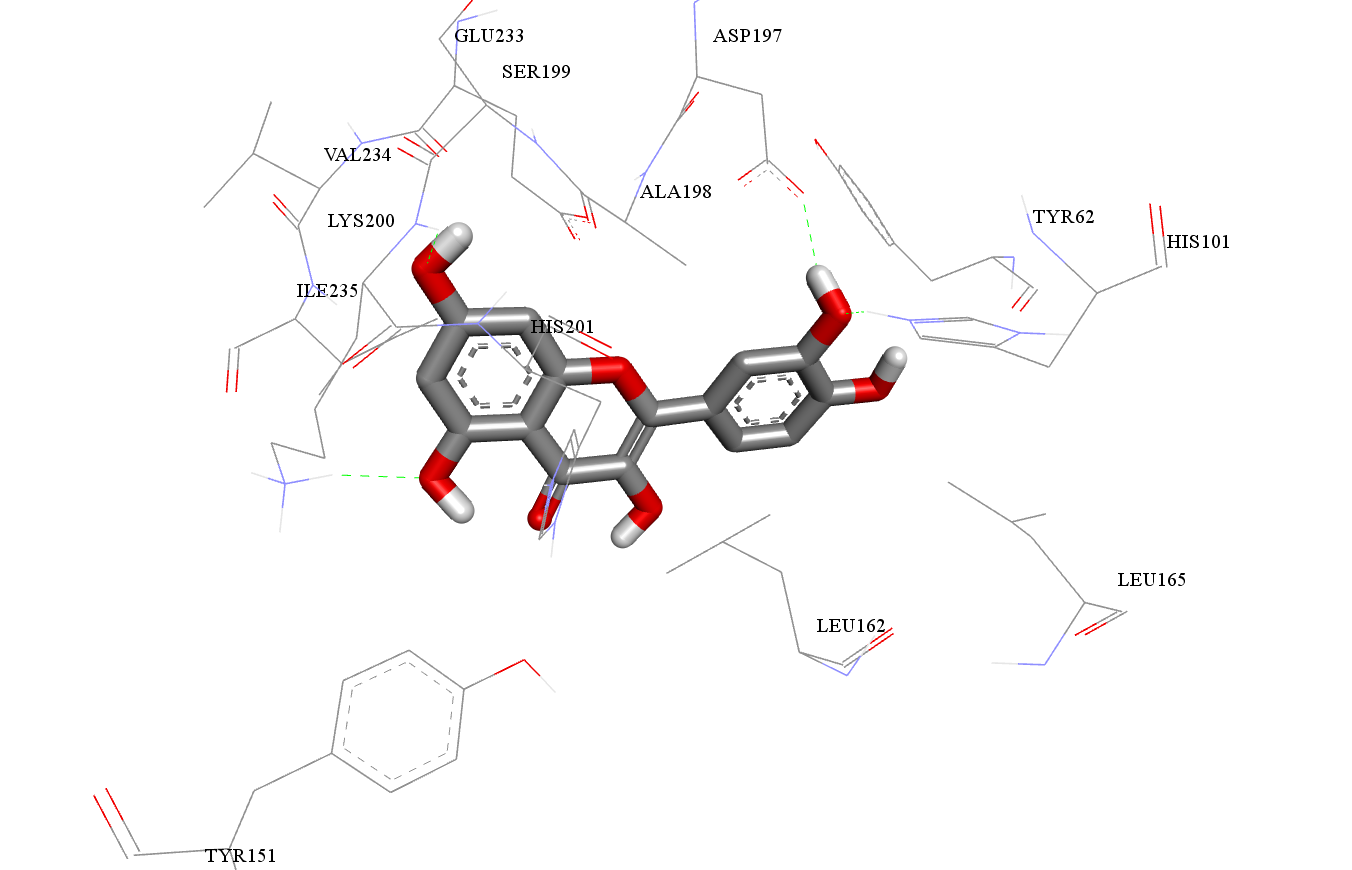


3D interaction image for 1DHK with Quercetin using Discovery studio, dashed green line show the hydrogen bonds.

Fig. S1. Visualization for docking of 1DHK with Quercetin

Table S2. Docking parameters for 1DHK with Oleic acid

| Oleic acid Efficiency | -0.25 |
| --- | --- |
| Inhibition constant | 205.76uM |
| Vdw_hb_desolv_energy | -9.78 |
| Electrostatic energy | -0.03 |
| Total internal energy | -0.86 |
| Torsional energy | 4.77 |
| Unbound energy | -0.86 |


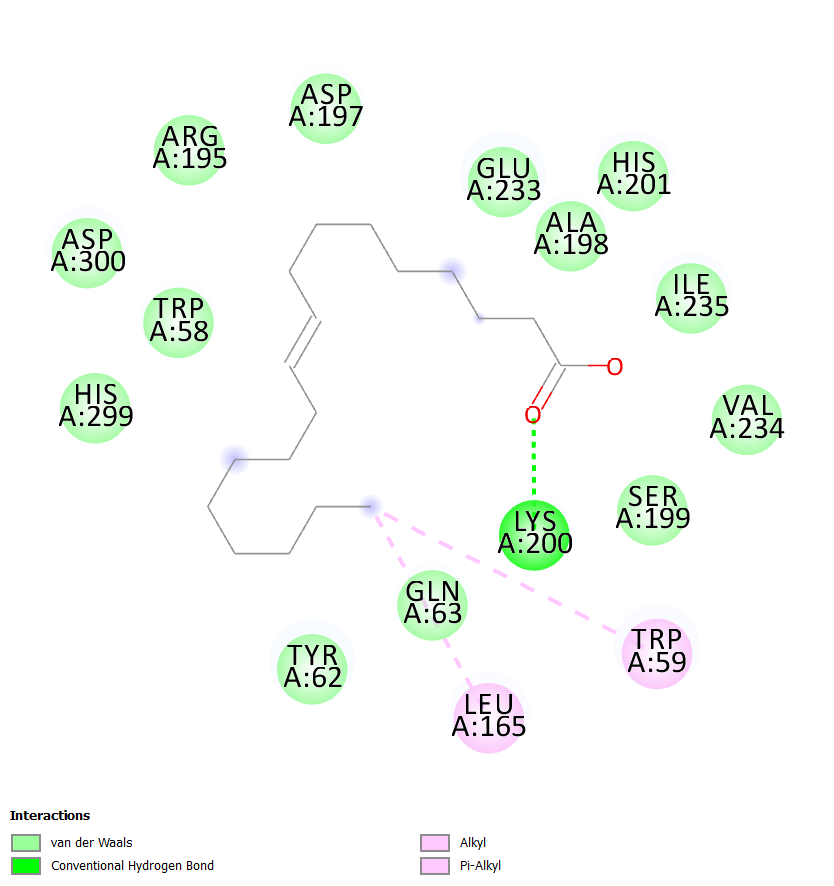


2D interaction image for 1DHK with Oleic acid


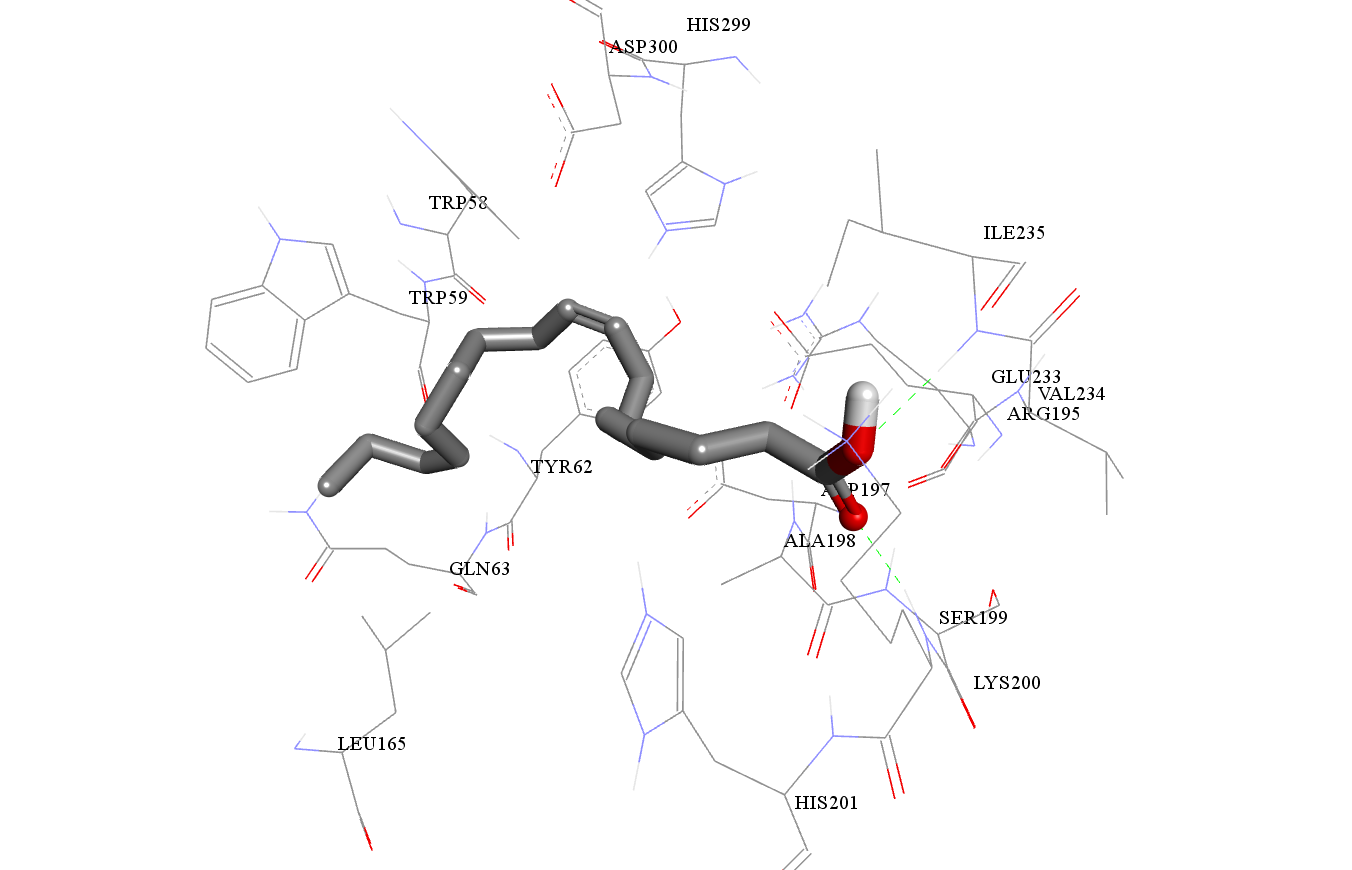


3D interaction image for 1DHK with Oleic acid

Fig. S2. Visualization for docking of 1DHK with Oleic acid

Table S3. Docking parameters for 1DHK with Diosgenin

| Diosgenin Efficiency | -0.31 |
| --- | --- |
| Inhibition constant | 123.44uM |
| Vdw_hb_desolv_energy | -9.42 |
| Electrostatic energy | -0.3 |
| Total internal energy | 0.02 |
| Torsional energy | 0.3 |
| Unbound energy | 0.02 |


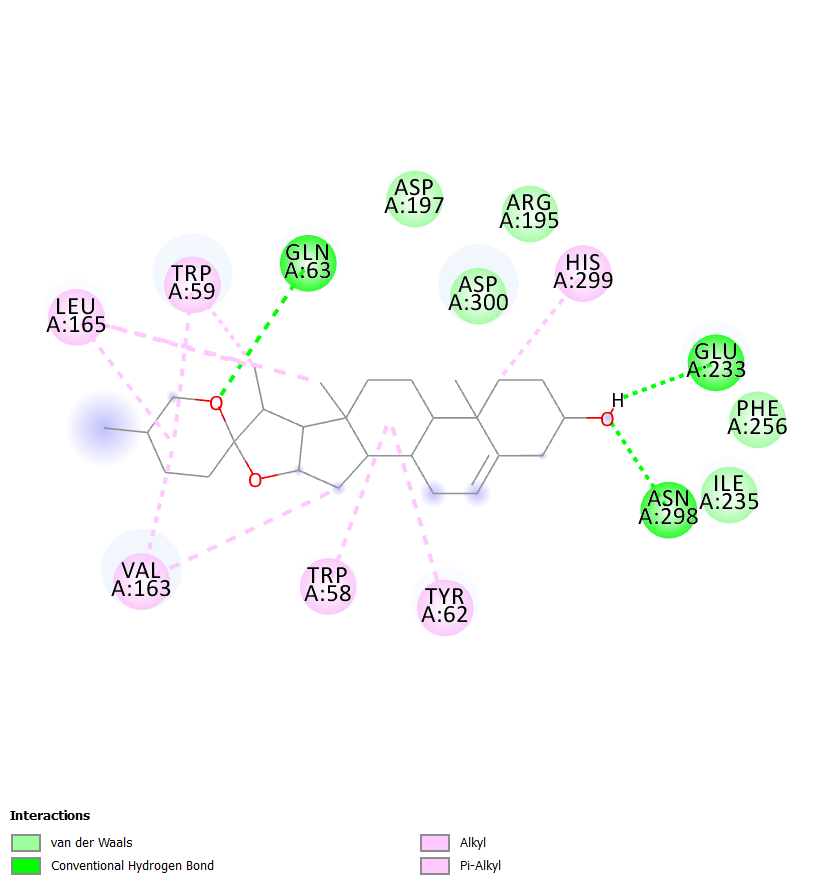


2D interaction image for 1DHK with Diosgenin


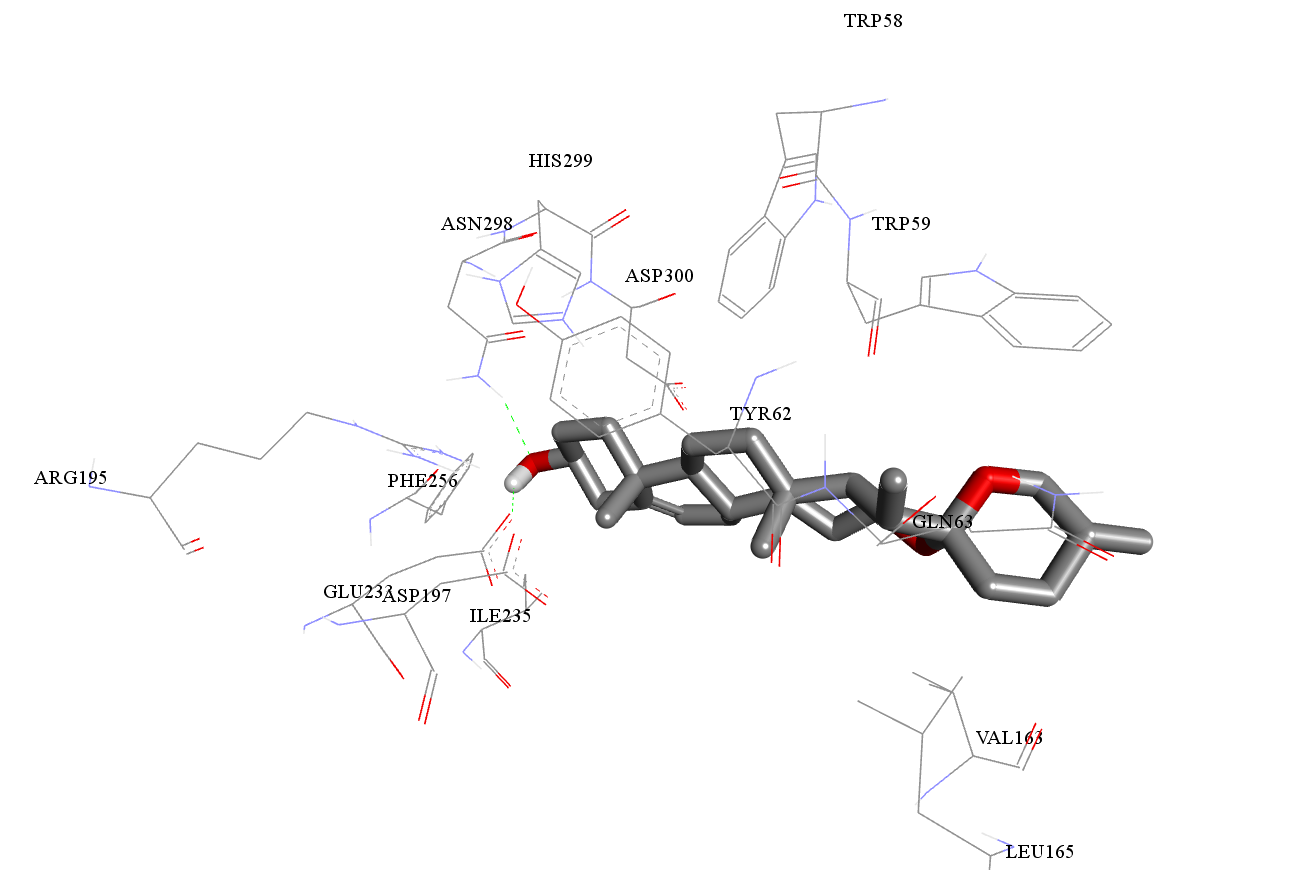


3D interaction image for 1DHK with Diosgenin

Fig. S3. Visualization for docking of 1DHK with Diosgenin

Table S4. Docking parameters for 1DHK with Corosolic acid

| Corosolic acid Efficiency | -0.27 |
| --- | --- |
| Inhibition constant | 222.25uM |
| Vdw_hb_desolv_energy | -9.88 |
| Electrostatic energy | -0.39 |
| Total internal energy | -0.11 |
| Torsional energy | 1.19 |
| Unbound energy | -0.11 |


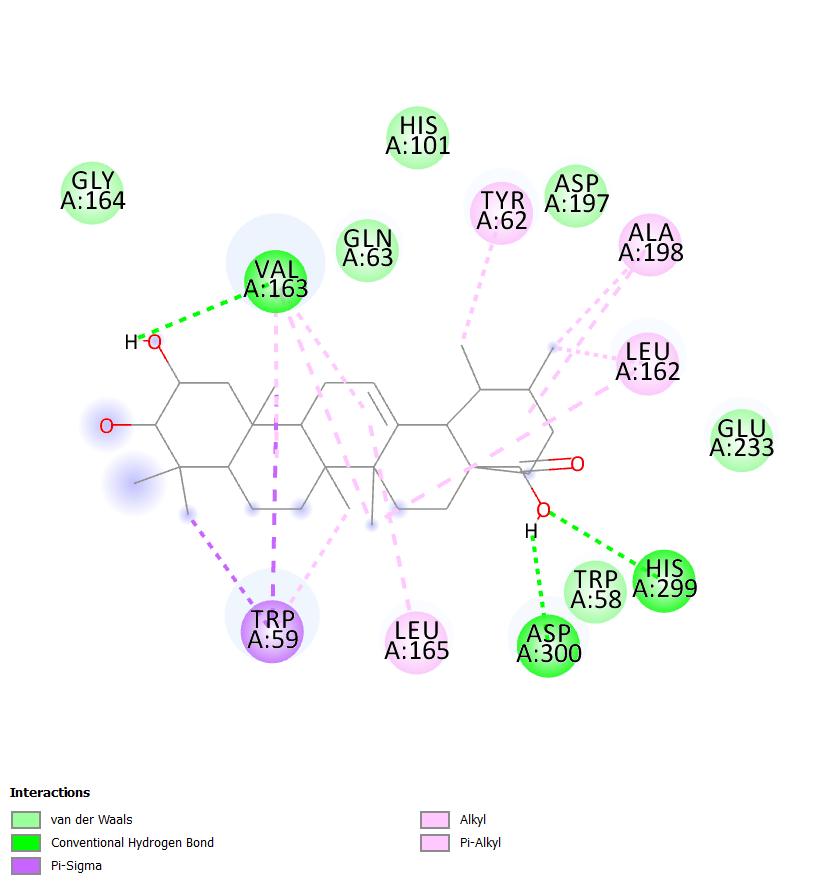


2D interaction image for 1DHK with Corosolic acid


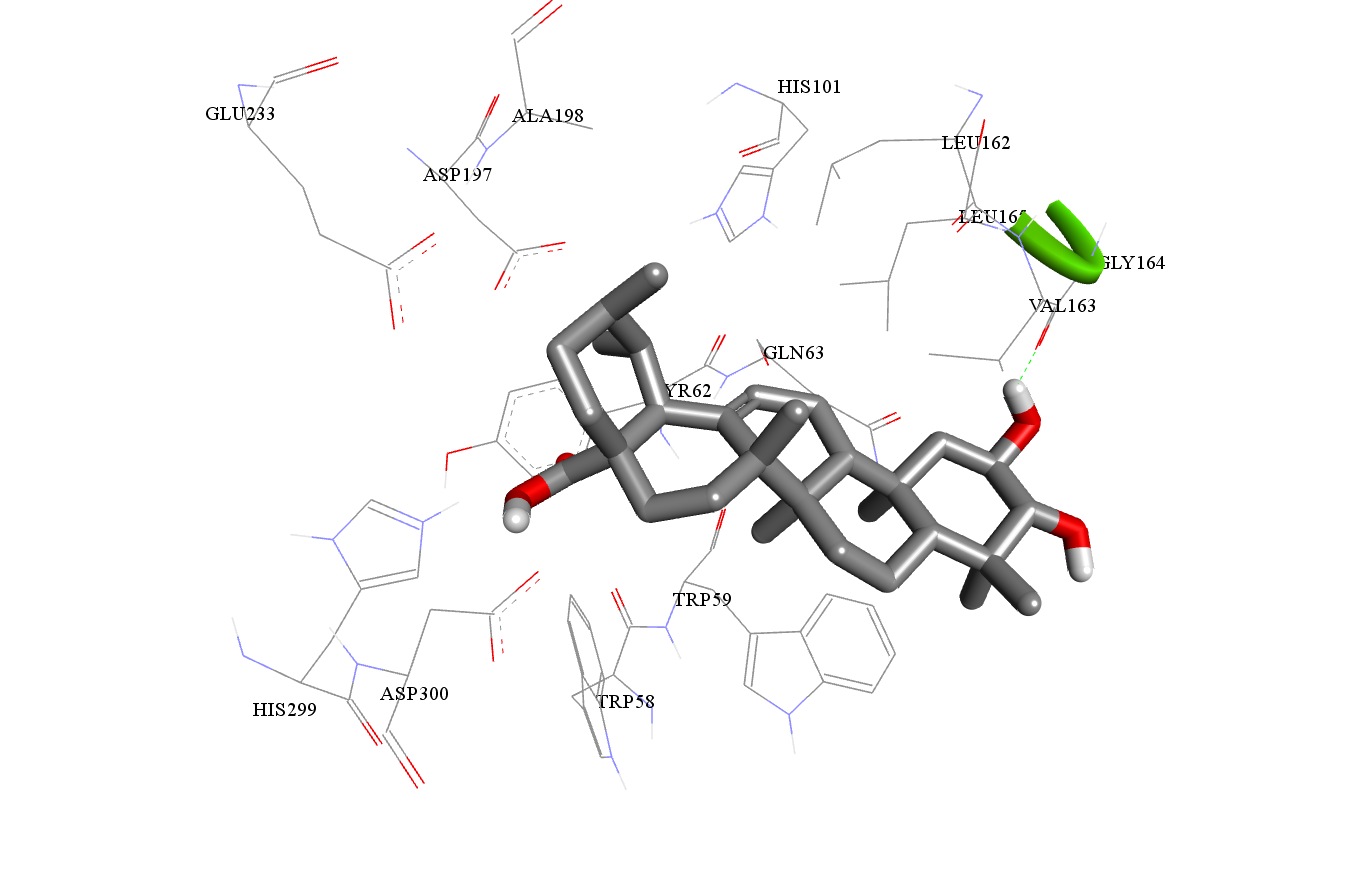


3D interaction image for 1DHK with Corosolic acid

Fig. S4. Visualization for docking of 1DHK with Corosolic acid

Table S5. Docking parameters for 1DHK with Cianidanol

| Cianidanol Efficiency | -0.34 |
| --- | --- |
| Inhibition constant | 6.48uM |
| Vdw_hb_desolv_energy | -8.05 |
| Electrostatic energy | -0.82 |
| Total internal energy | -0.25 |
| Torsional energy | 1.79 |
| Unbound energy | -0.25 |


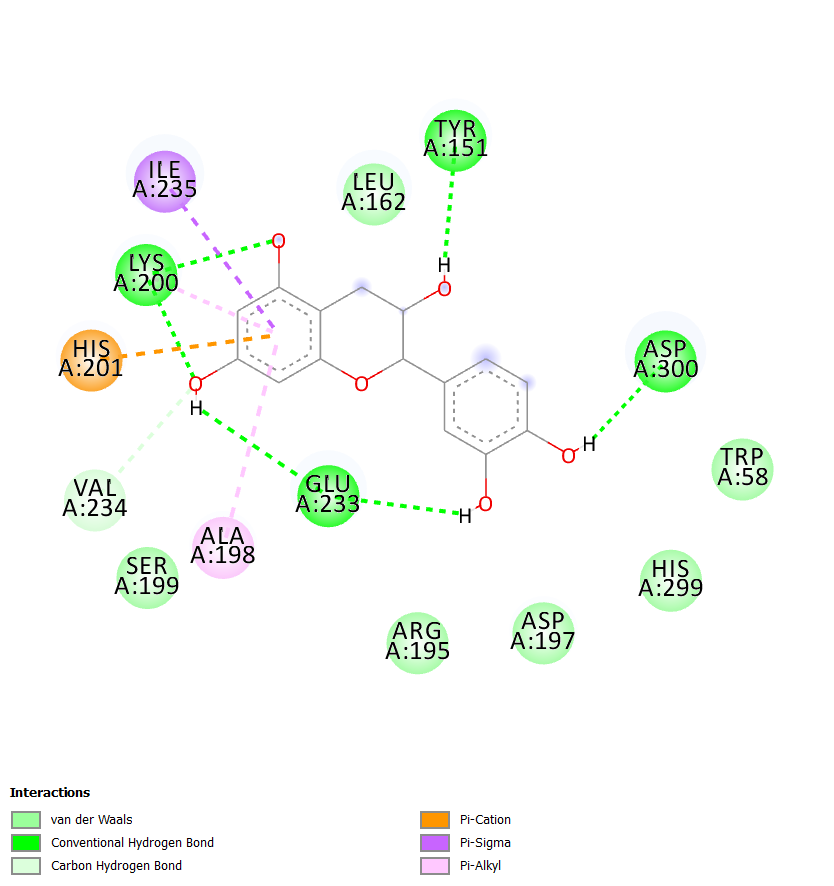


2D interaction image for 1DHK with Cianidanol


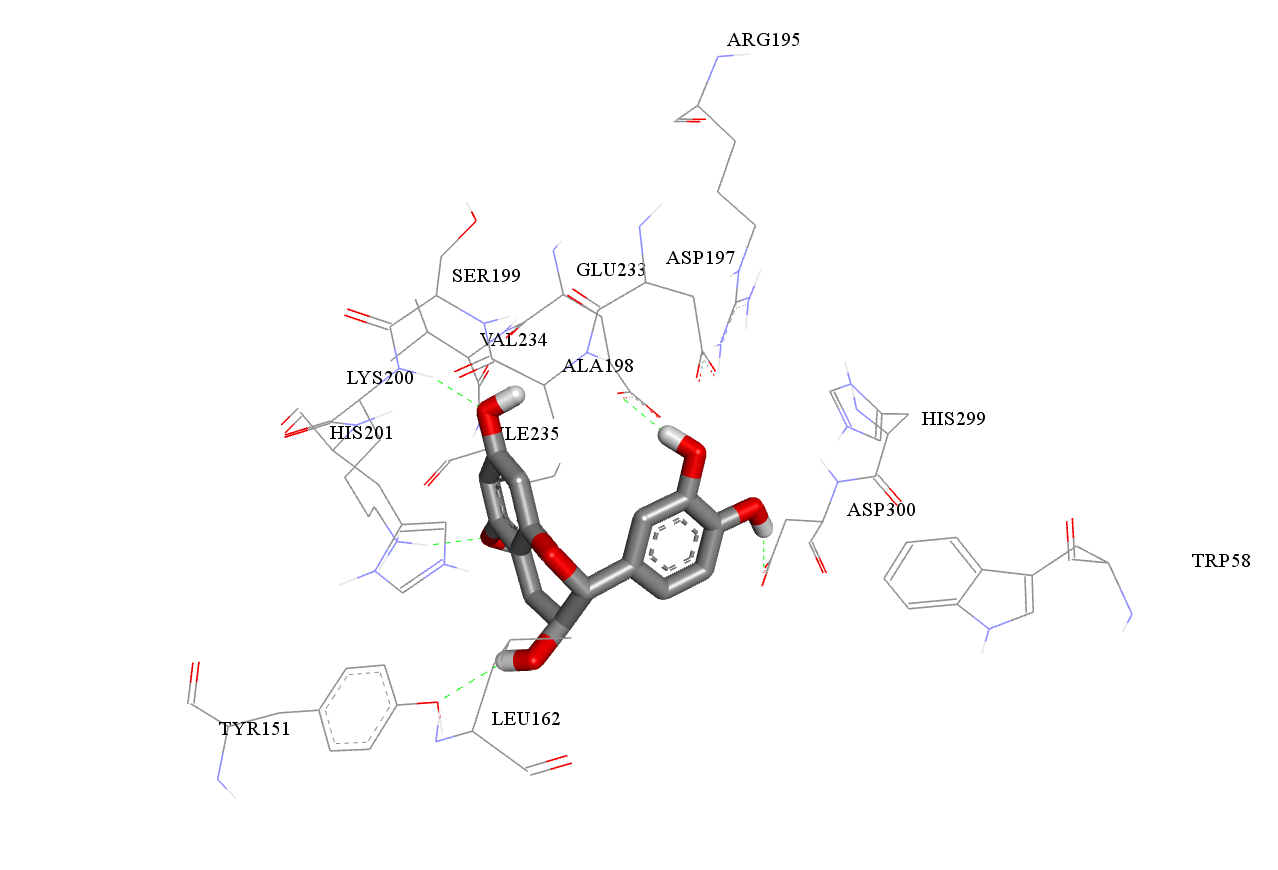


3D interaction image for 1DHK with Cianidanol

Fig. S5. Visualization for docking of 1DHK with Cianidanol

Table S6. Docking parameters for 1DHK with β-sitosterol

| β- Sitosterol Efficiency | -0.3 |
| --- | --- |
| Inhibition constant | 238.83nM |
| Vdw_hb_desolv_energy | -10.86 |
| Electrostatic energy | -0.26 |
| Total internal energy | -0.89 |
| Torsional energy | 2.09 |
| Unbound energy | -0.89 |


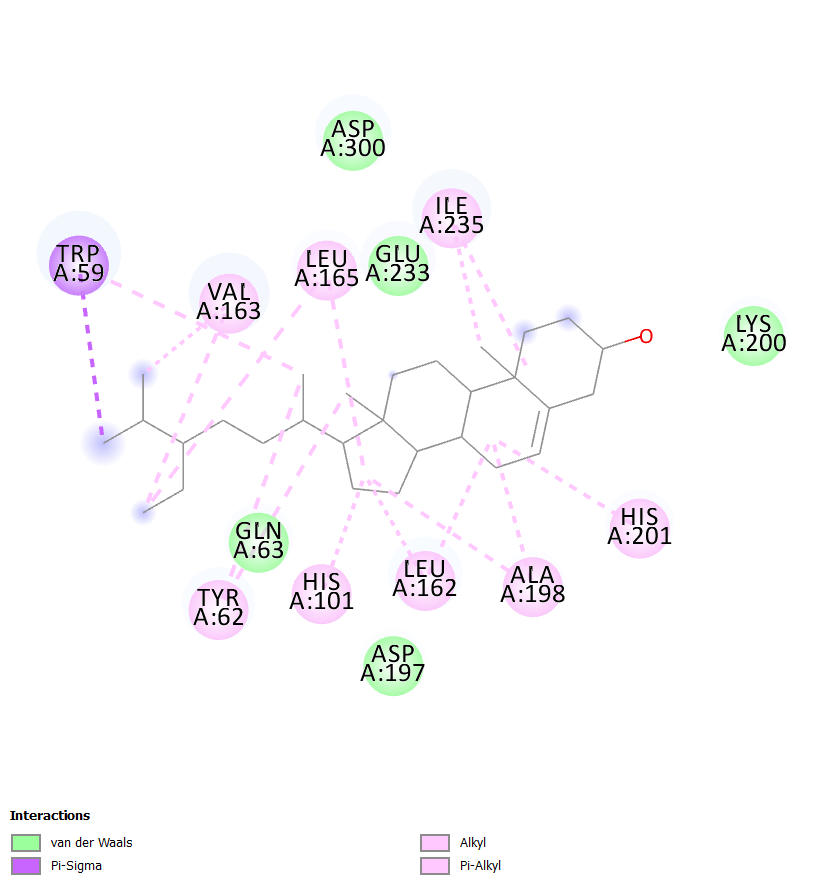


2D interaction image for 1DHK with β-sitosterol


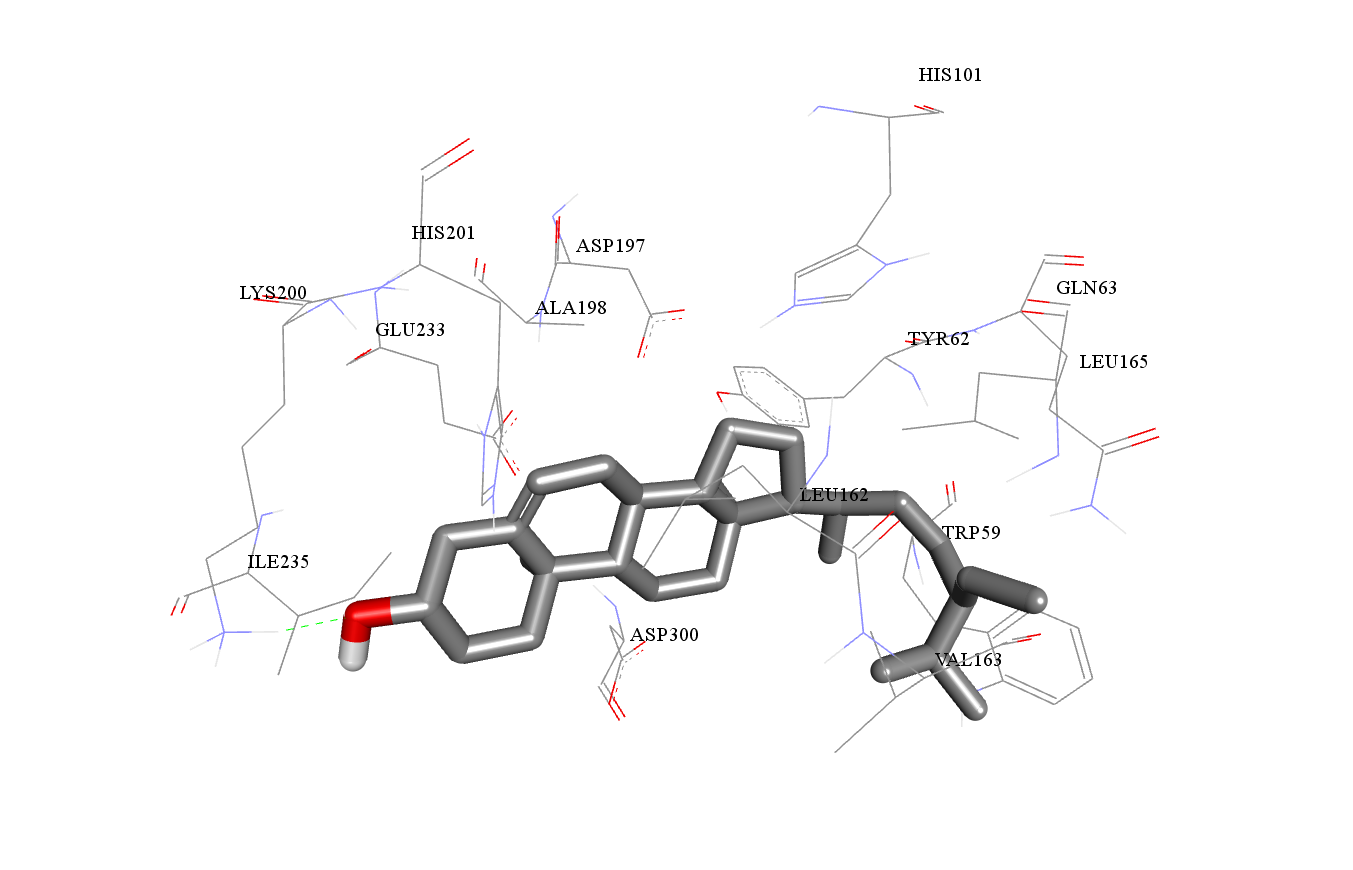


3D interaction image for 1DHK with β-sitosterol

Fig. S6. Visualization for docking of 1DHK with β-sitosterol

Table S7. Docking parameters for 1DHK with ‘reference- Acarbose’

| ‘reference’ Efficiency | -0.1 |
| --- | --- |
| Inhibition constant | 513.96uM |
| Vdw_hb_desolv_energy | -10.59 |
| Electrostatic energy | -0.46 |
| Total internal energy | -4.51 |
| Torsional energy | 6.56 |
| Unbound energy | -4.51 |


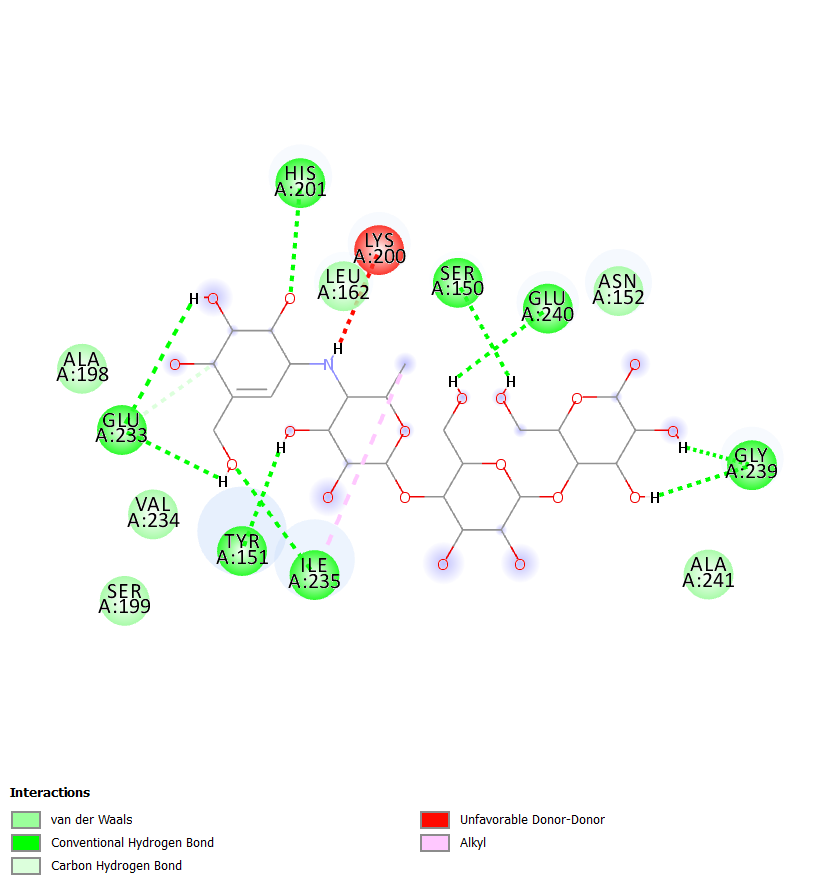


2D interaction image for 1DHK with ‘reference- Acarbose’


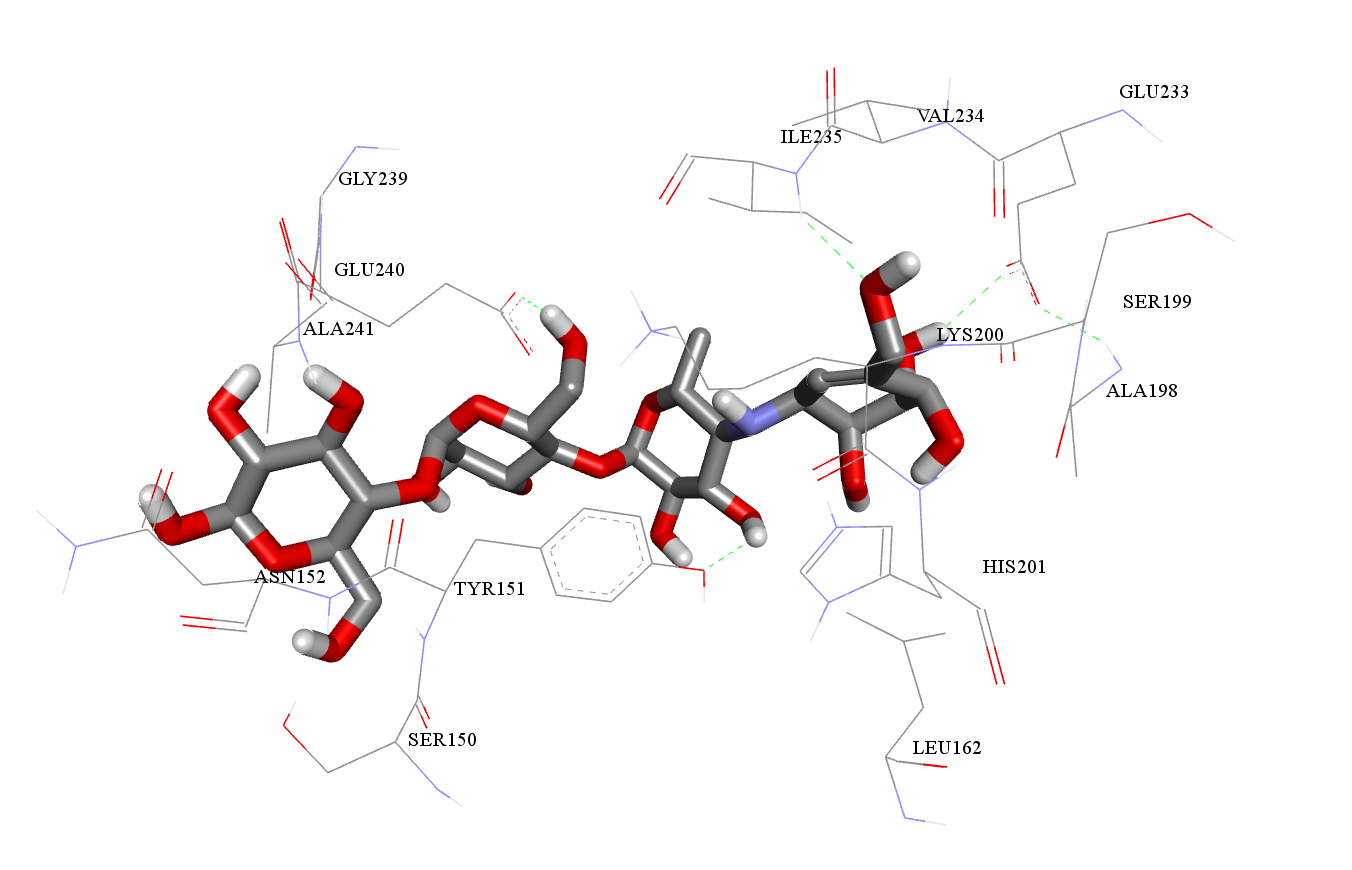


3D interaction image for 1DHK with ‘reference- Acarbose’

Fig. S7. Visualization for docking of 1DHK with ‘reference- Acarbose’

**3.6.3.2 Elucidating molecular docking interactions of compounds with α-glucosidase**

Table S8. Docking parameters for 4J5T with Quercetin

| Quercetin Efficiency | -0.39 |
| --- | --- |
| Inhibition constant | 517.53nM |
| Vdw_hb_desolv_energy | -10.05 |
| Electrostatic energy | -0.31 |
| Total internal energy | -1.54 |
| Torsional energy | 1.79 |
| Unbound energy | -1.54 |


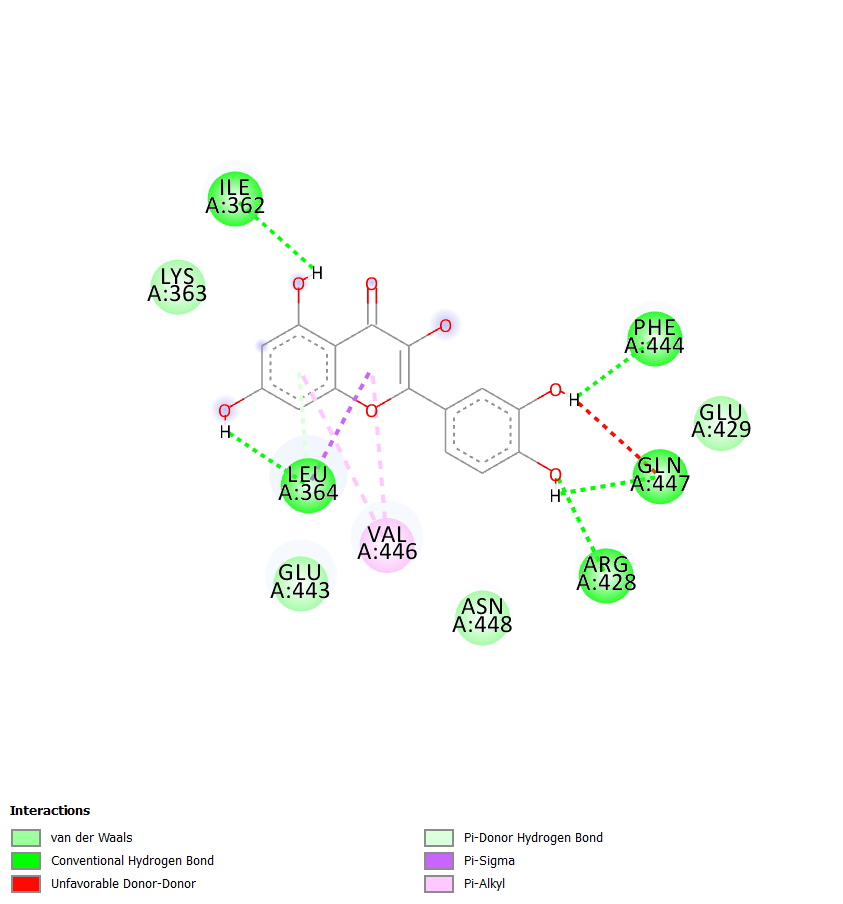


2D interaction image for 4J5T with Quercetin


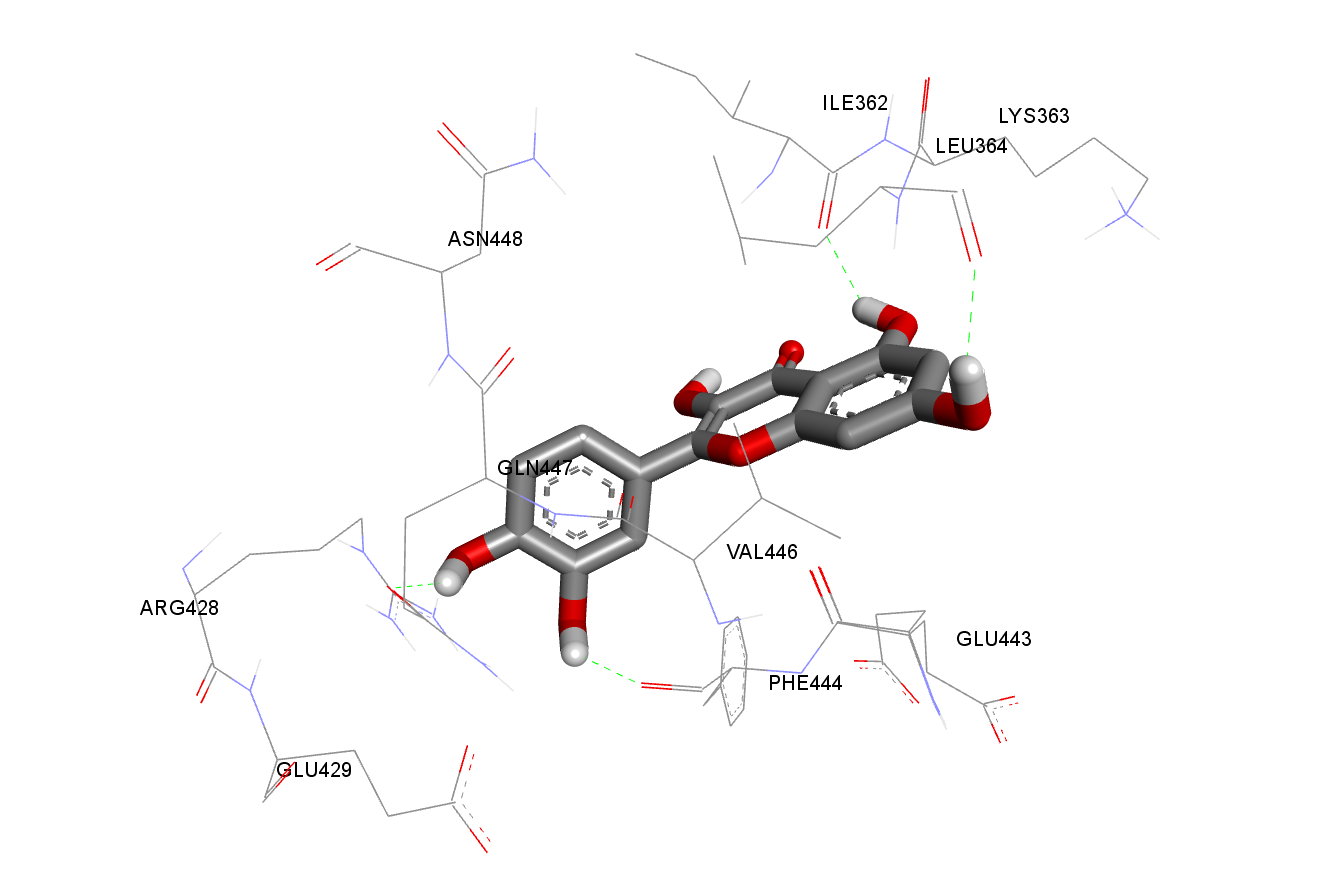


3D interaction image for 4J5T with Quercetin

Fig. S8. Visualization for docking of 4J5T with Quercetin

Table S9. Docking parameters for 4J5T with Oleic acid

| Oleic acid Efficiency | -0.31 |
| --- | --- |
| Inhibition constant | 25.2Um |
| Vdw_hb_desolv_energy | -10.96 |
| Electrostatic energy | -0.08 |
| Total internal energy | -0.85 |
| Torsional energy | 4.77 |
| Unbound energy | -0.85 |


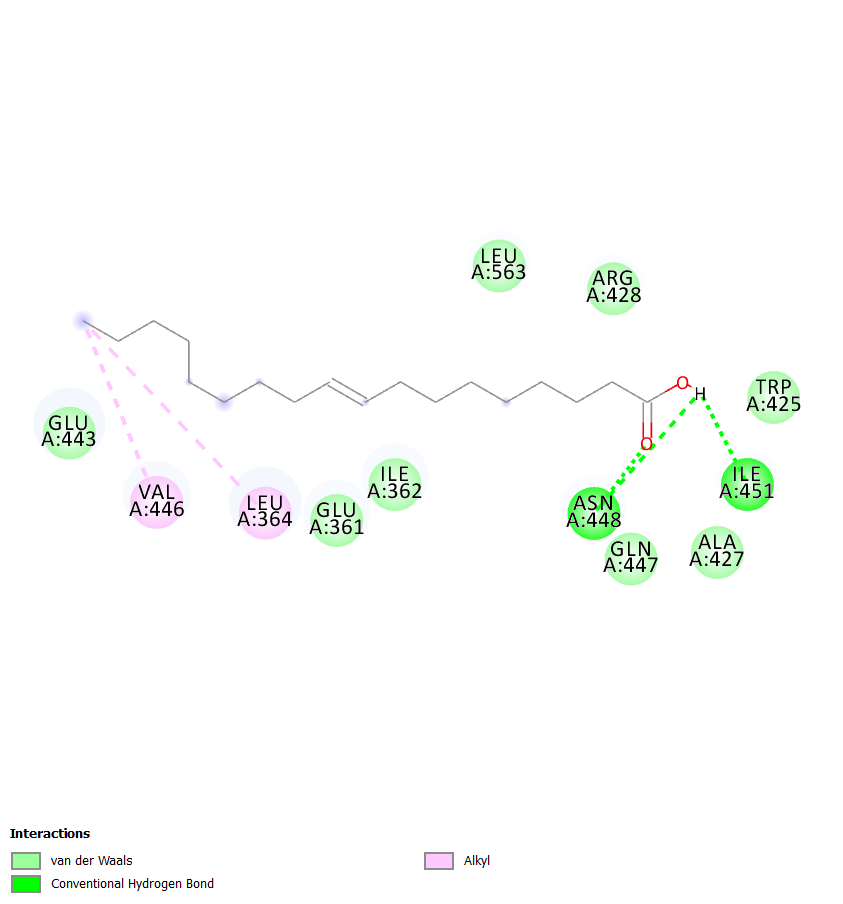


2D interaction image for 4J5T with Oleic acid


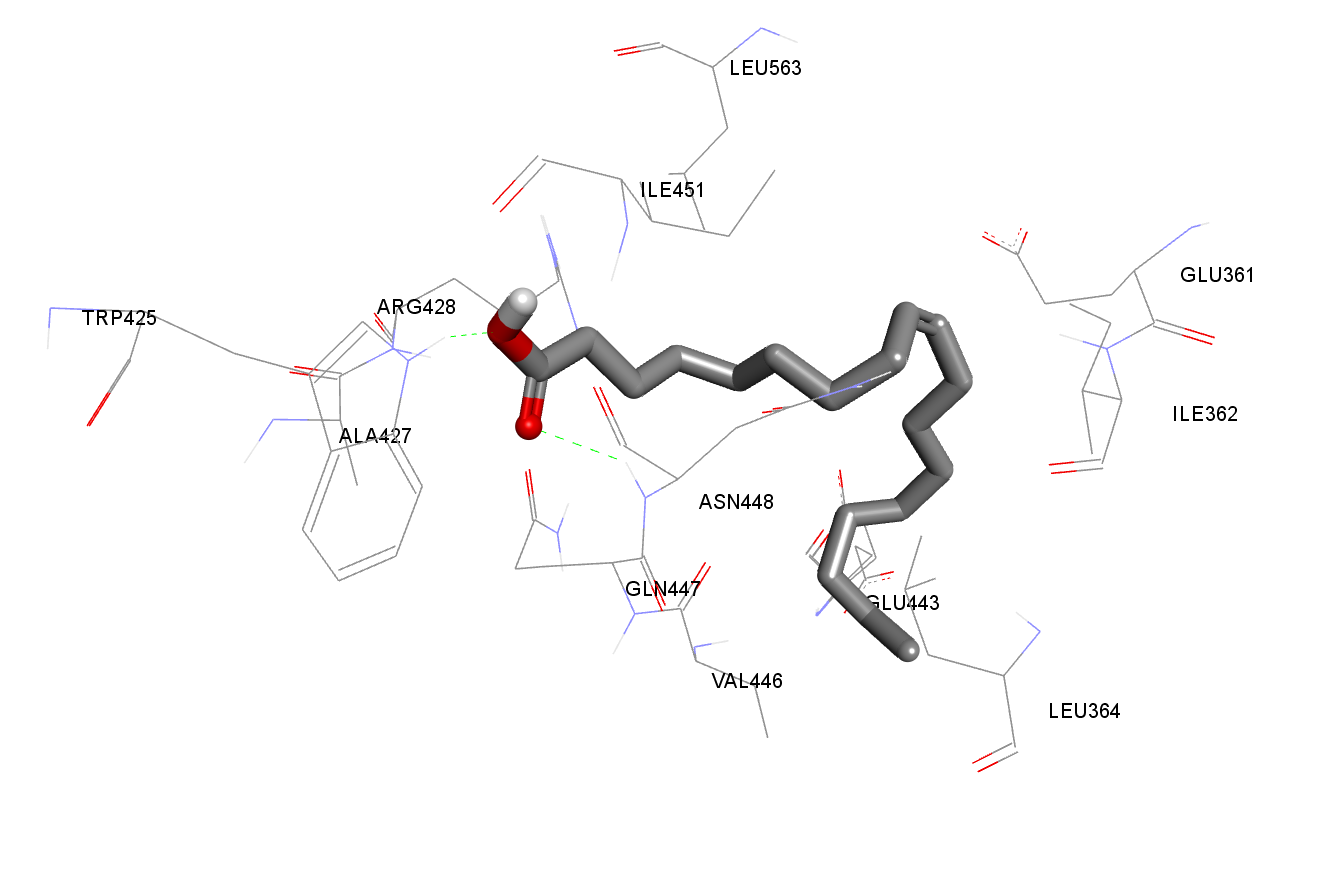


3D interaction image for 4J5T with Oleic acid

Fig. S9. Visualization for docking of 4J5T with Oleic acid

Table S10. Docking parameters for 4J5T with Diosgenin

| Diosgenin Efficiency | -0.28 |
| --- | --- |
| Inhibition constant | 684.57uM |
| Vdw_hb_desolv_energy | -8.59 |
| Electrostatic energy | -0.12 |
| Total internal energy | 0.02 |
| Torsional energy | 0.3 |
| Unbound energy | 0.02 |


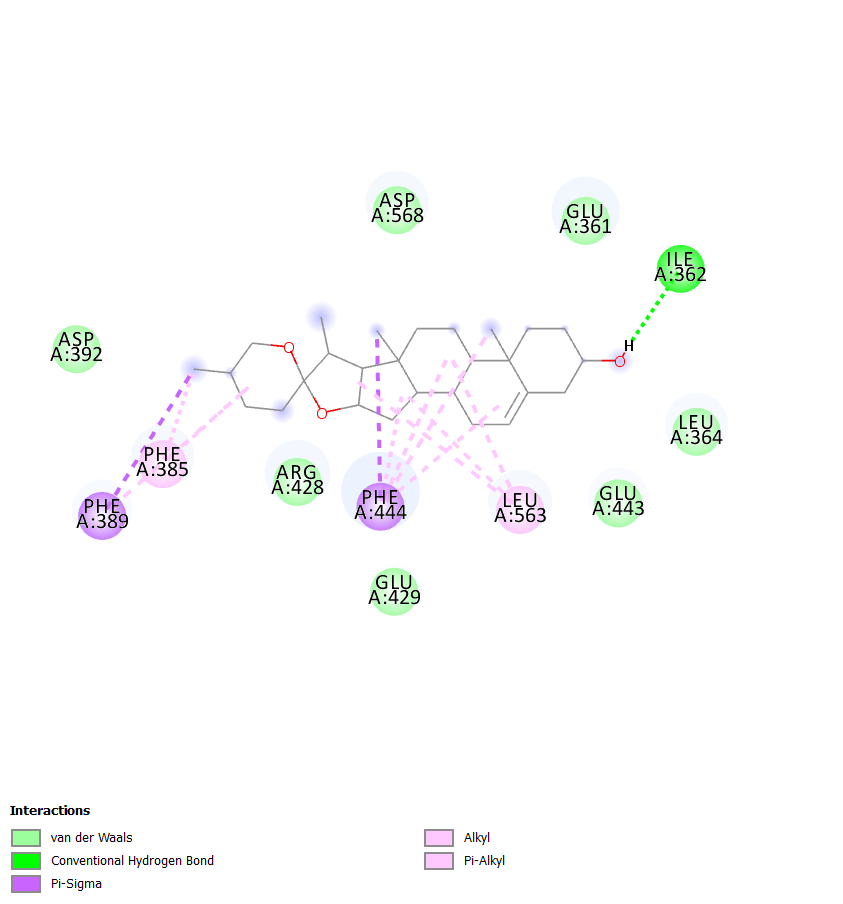


2D interaction image for 4J5T with Diosgenin


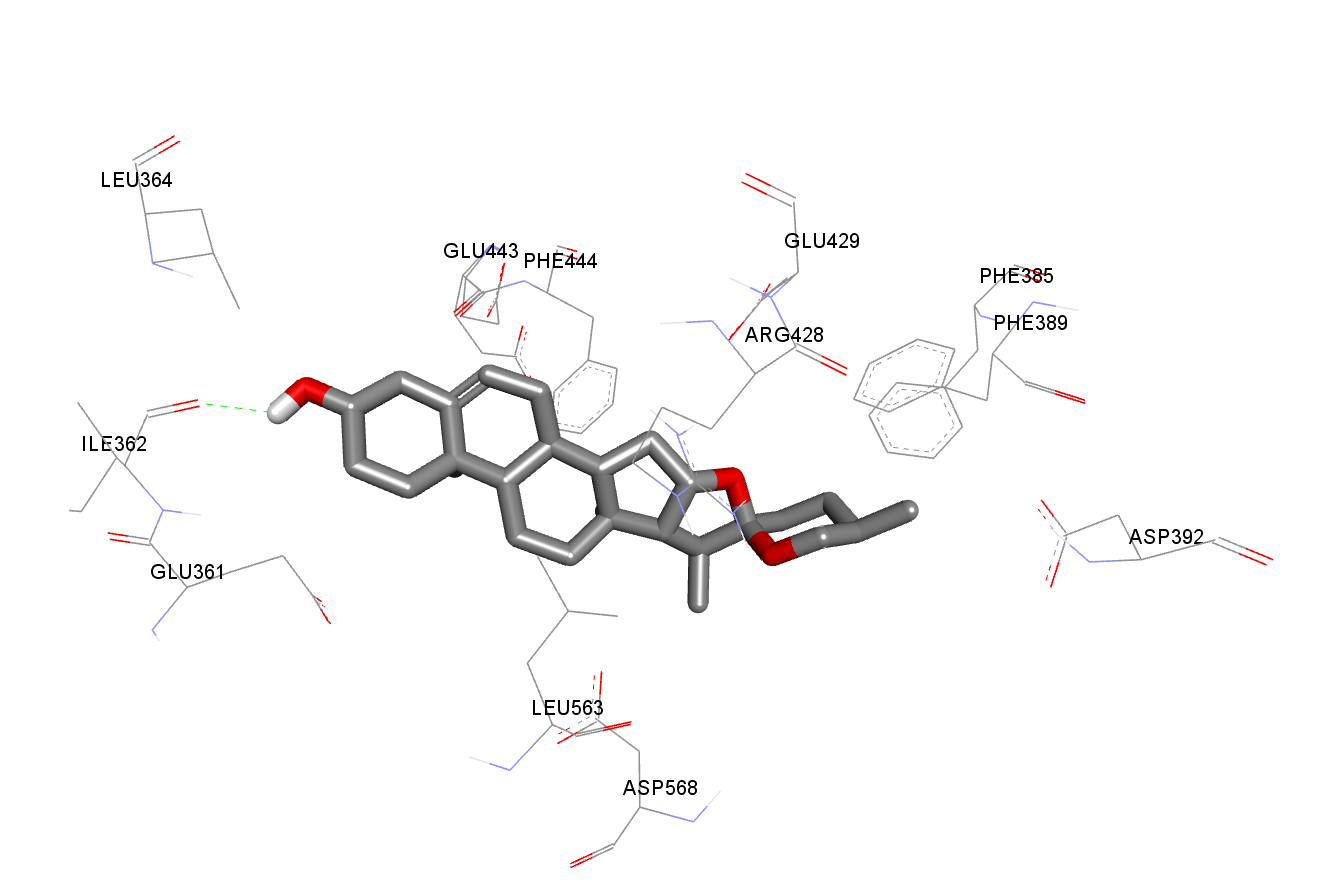


3D interaction image for 4J5T with Diosgenin

Fig. S10. Visualization for docking of 4J5T with Diosgenin

Table S11. Docking parameters for 4J5T with Corosolic acid

| Corosolic acid Efficiency | -0.29 |
| --- | --- |
| Inhibition constant | 74.92uM |
| Vdw_hb_desolv_energy | -10.8 |
| Electrostatic energy | -0.11 |
| Total internal energy | -0.48 |
| Torsional energy | 1.19 |
| Unbound energy | -0.48 |


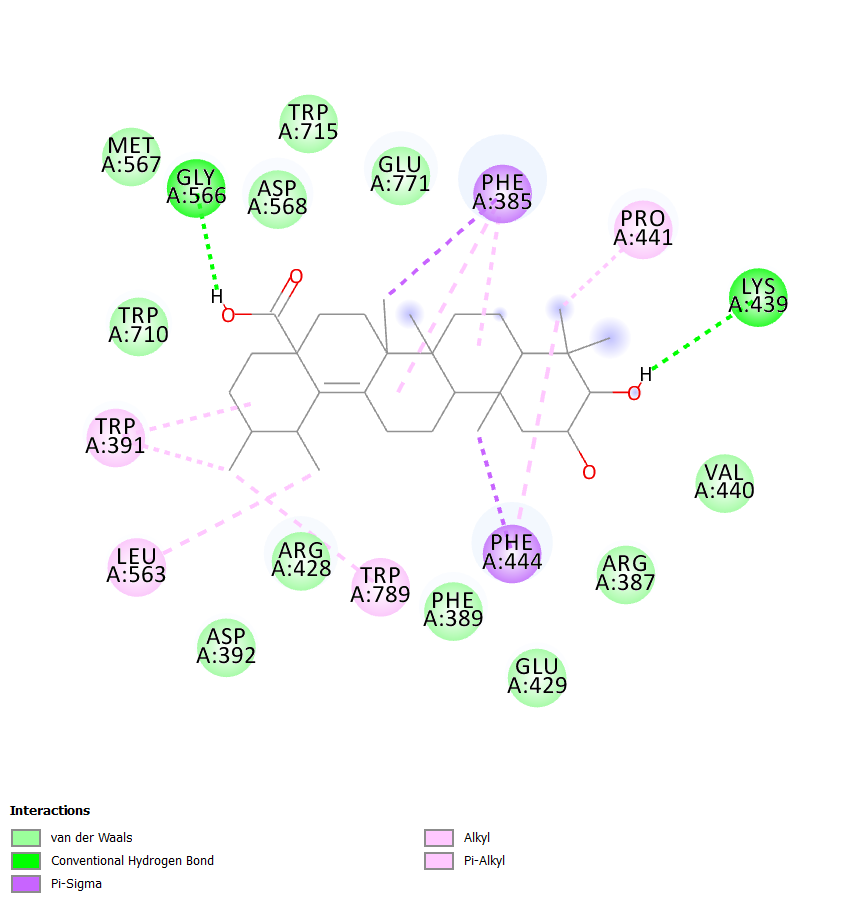


2D interaction image for 4J5T with Corosolic acid


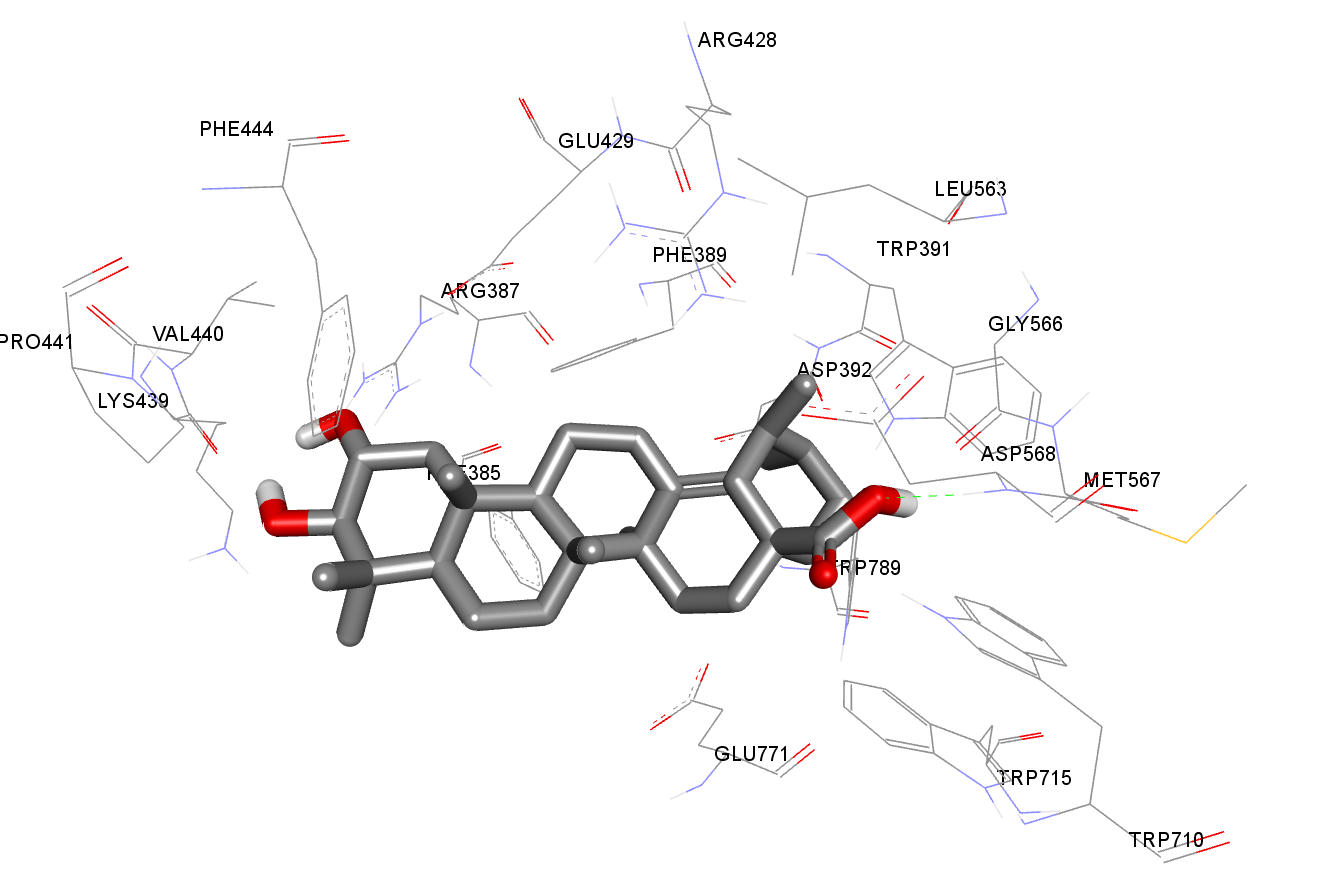


3D interaction image for 4J5T with Corosolic acid

Fig. S11. Visualization for docking of 4J5T with Corosolic acid

Table S12. Docking parameters for 4J5T with Cianidanol

| Cianidanol Efficiency | -0.41 |
| --- | --- |
| Inhibition constant | 414.53uM |
| Vdw_hb_desolv_energy | -9.7 |
| Electrostatic energy | -0.8 |
| Total internal energy | -0.29 |
| Torsional energy | 1.79 |
| Unbound energy | -0.29 |


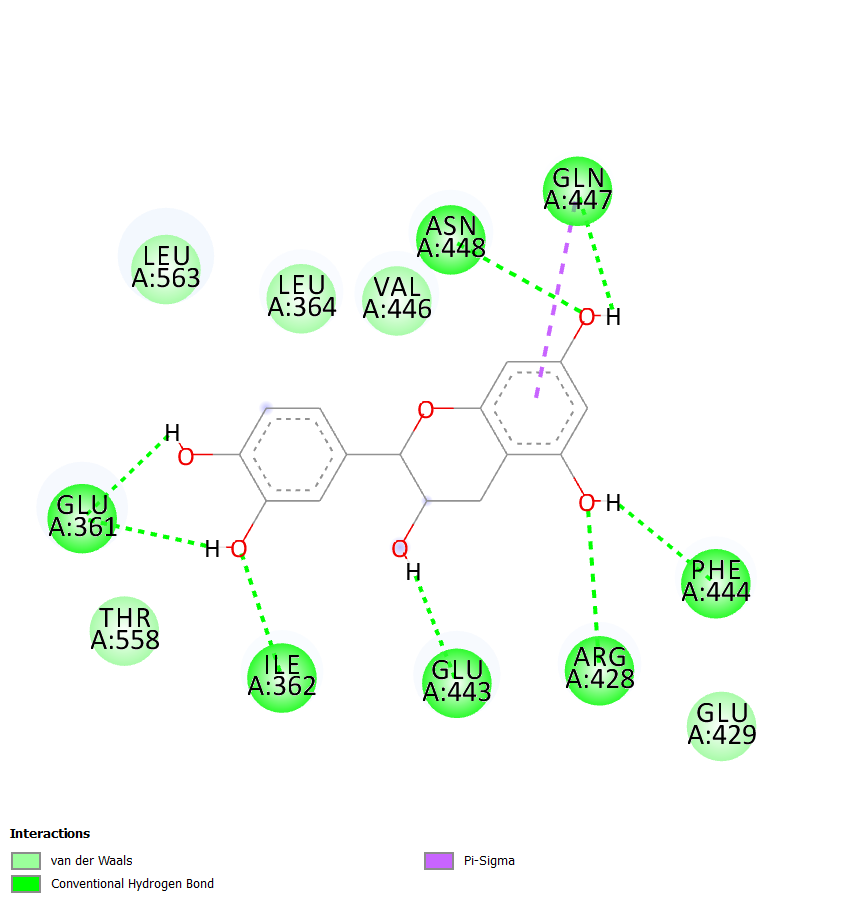


2D interaction image for 4J5T with Cianidanol


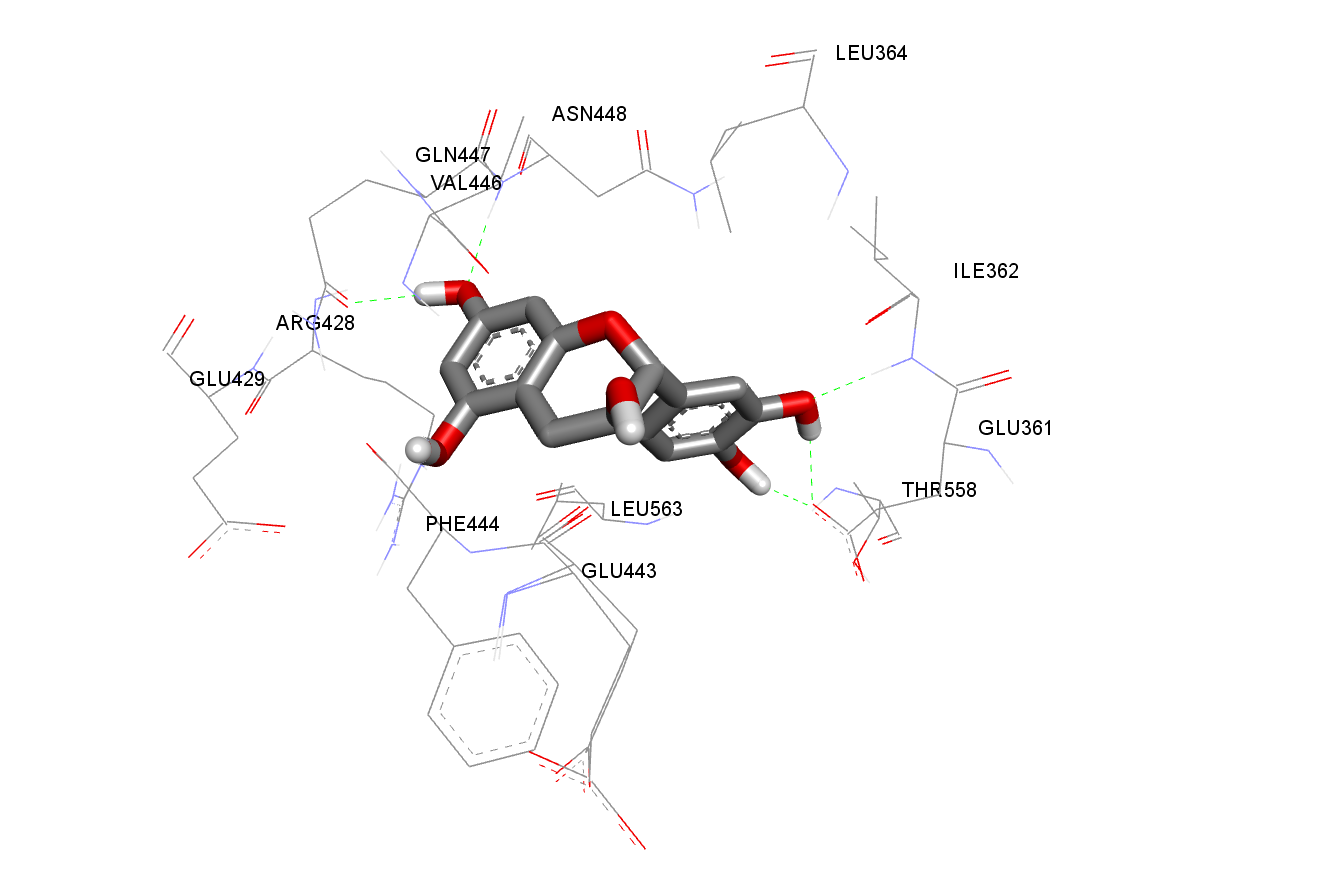


3D interaction image for 4J5T with Cianidanol

Fig. S12. Visualization for docking of 4J5T with Cianidanol

Table S13. Docking parameters for 4J5T with β-sitosterol

| β- Sitosterol Efficiency | -0.3 |
| --- | --- |
| Inhibition constant | 282.53nM |
| Vdw_hb_desolv_energy | -10.82 |
| Electrostatic energy | -0.21 |
| Total internal energy | -0.67 |
| Torsional energy | 2.09 |
| Unbound energy | -0.67 |


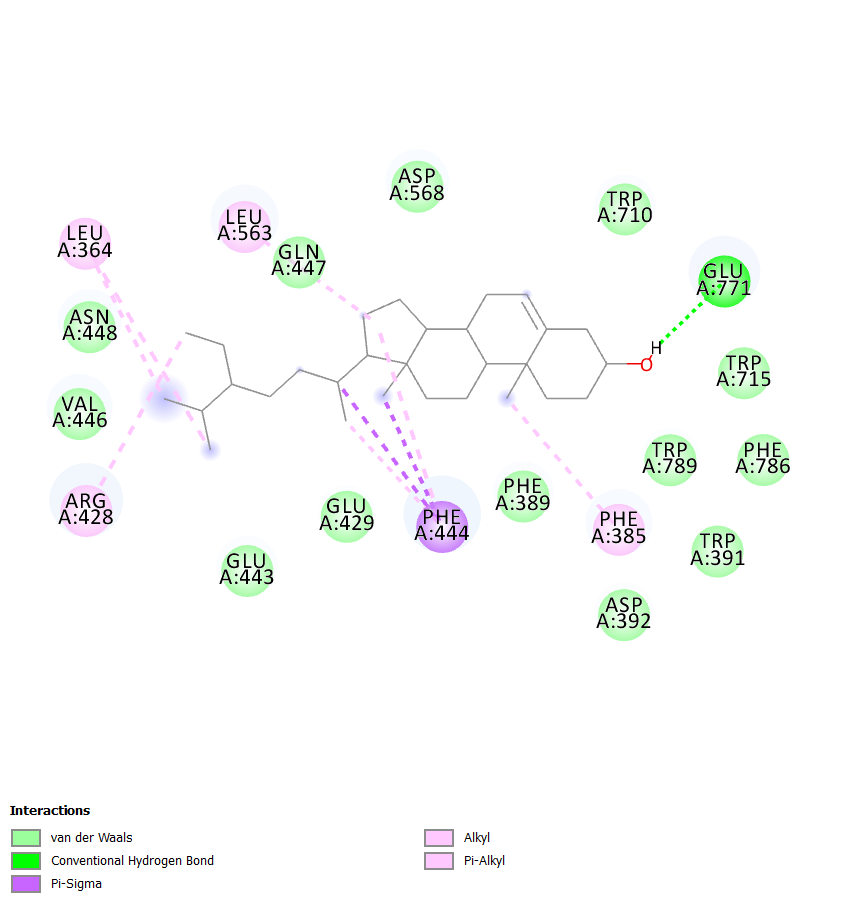


2D interaction image for 4J5T with β-sitosterol


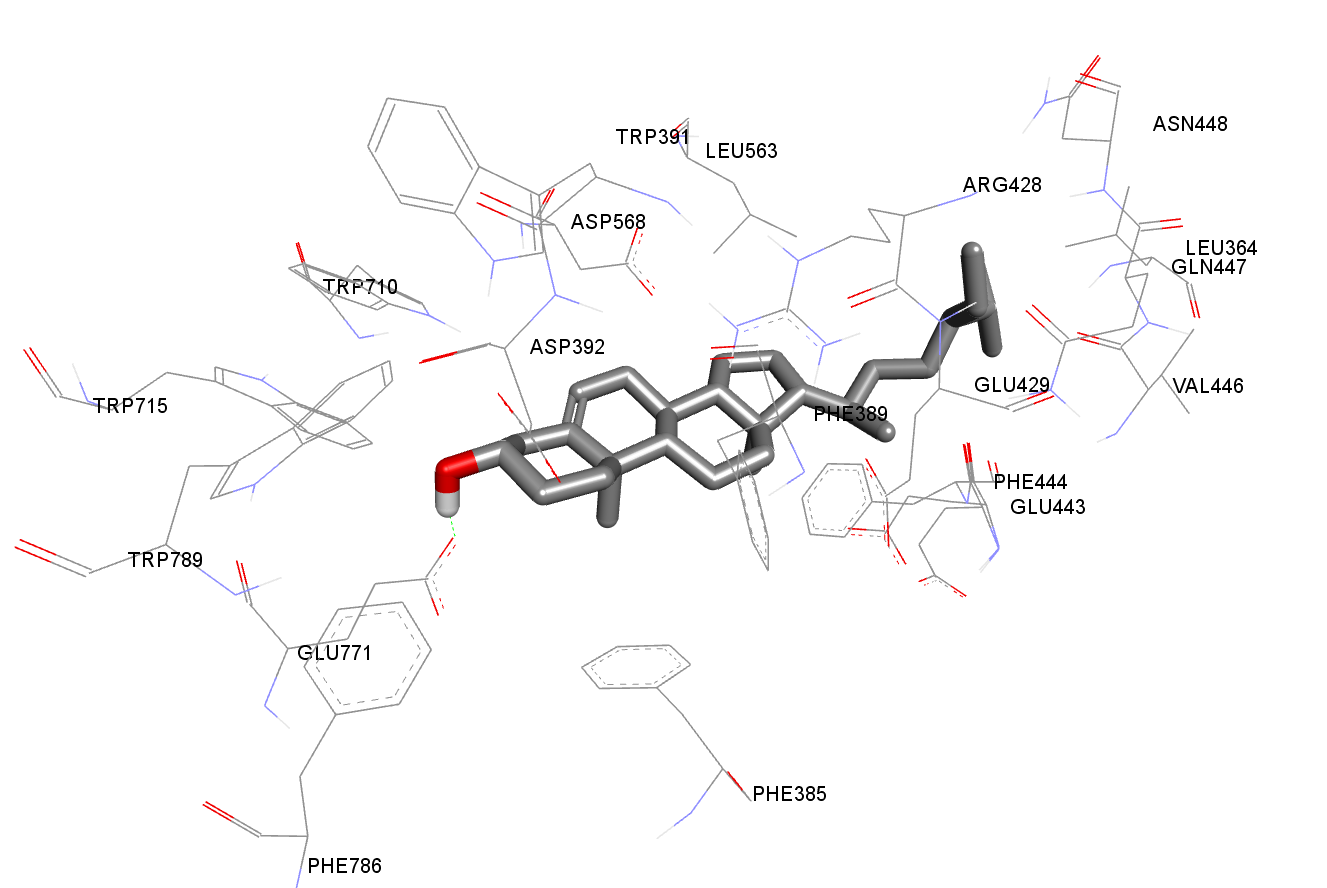


3D interaction image for 4J5T with β-sitosterol

Fig. S13. Visualization for docking of 4J5T with β-sitosterol

Table S14. Docking parameters for 4J5T with ‘reference- Acarbose’

| ‘reference’ Efficiency | -0.17 |
| --- | --- |
| Inhibition constant | 3.57Um |
| Vdw_hb_desolv_energy | -12.92 |
| Electrostatic energy | -1.08 |
| Total internal energy | -4.2 |
| Torsional energy | 6.56 |
| Unbound energy | -4.2 |


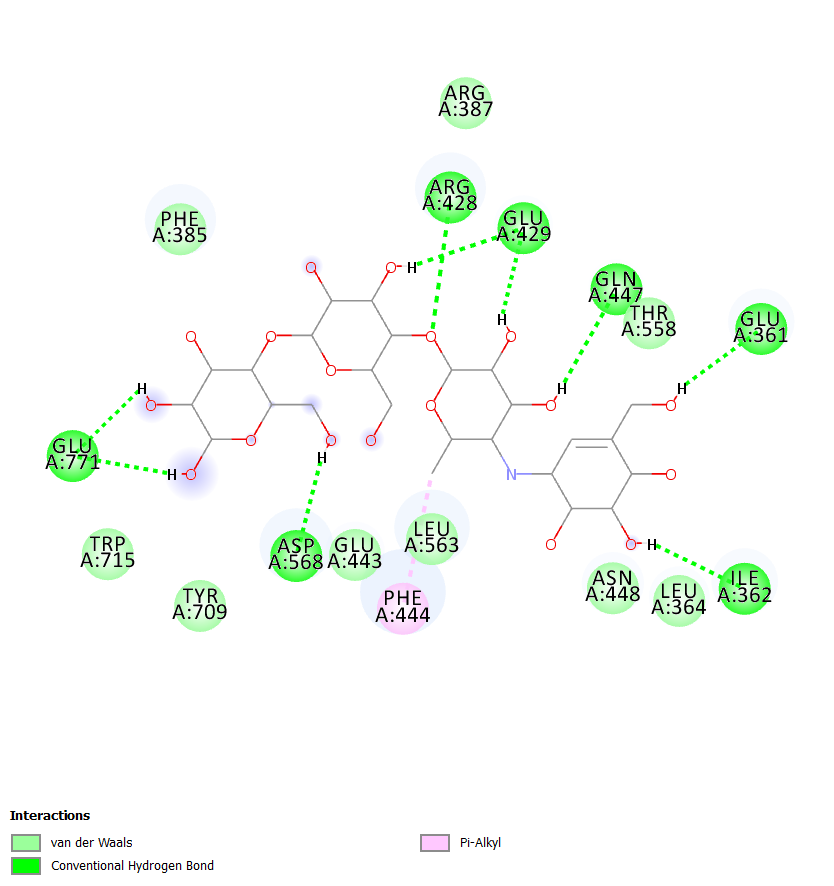


2D interaction image for 4J5T with ‘reference- Acarbose’


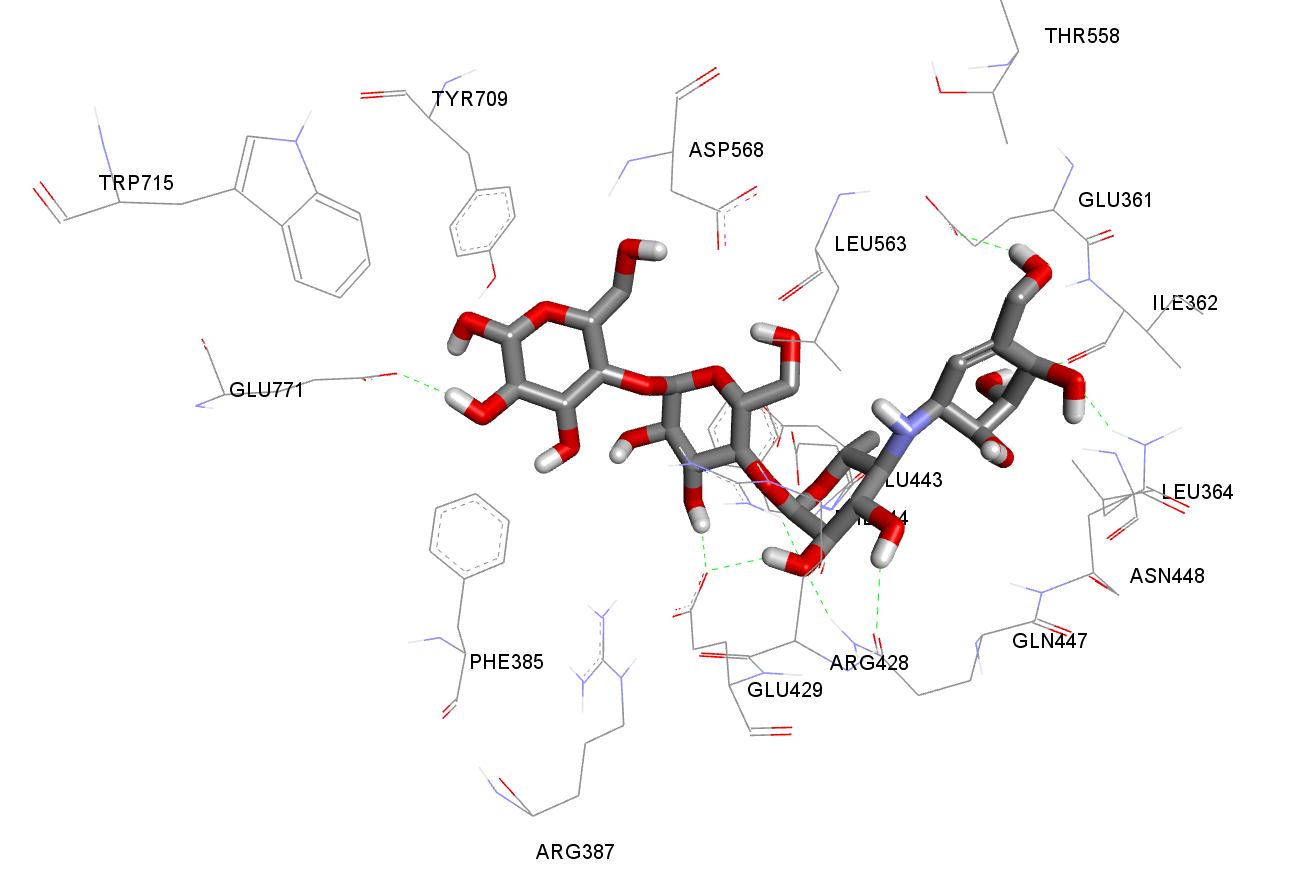


3D interaction image for 4J5T with ‘reference- Acarbose’

Fig. S14. Visualization for docking of 4J5T with ‘reference- Acarbose’
